# TGFβ attenuates cartilage extracellular matrix degradation via enhancing FBXO6-mediated MMP14 ubiquitination

**DOI:** 10.1136/annrheumdis-2019-216911

**Published:** 2020-05-14

**Authors:** Gangliang Wang, Shuai Chen, Ziang Xie, Shuying Shen, Wenbin Xu, Wenxiang Chen, Xiang Li, Yizheng Wu, Liangping Li, Bin Liu, Xianjun Ding, An Qin, Shunwu Fan

**Affiliations:** 1 Department of Orthopaedics, Sir Run Run Shaw Hospital, School of Medicine, Zhejiang University, Hangzhou, Zhejiang, China; 2 Key Laboratory of Musculoskeletal System Degeneration and Regeneration Translational Research of Zhejiang Province, Hangzhou, Zhejiang, China; 3 Key Laboratory of Protein Modification and Tumor, Hubei Polytechnic University School of Medicine, Huangshi, Hubei, China; 4 Department of Orthopedics, Shanghai Key Laboratory of Orthopedic Implants, Shanghai Ninth People's Hospital, Shanghai Jiaotong University School of Medicine, Shanghai, China

**Keywords:** osteoarthritis, chondrocytes, cytokines

## Abstract

**Objectives:**

FBXO6, a component of the ubiquitin E3 ligases, has been shown to bind high mannose N-linked glycoproteins and act as ubiquitin ligase subunits. Most proteins in the secretory pathway, such as matrix metalloproteinases, are modified with N-glycans and play important roles in the development of osteoarthritis (OA). However, whether FBXO6 exerts regulatory effects on the pathogenesis of OA remains undefined.

**Methods:**

The expression of FBXO6 was examined in the cartilage of human and multiple mouse OA models. The role of FBXO6 in cartilage degeneration was analysed with *global FBXO6* ^*-/-*^ mice, transgenic *Col2a1-CreER^T2^;FBXO6^f/f^* mice. The FBXO6 interacting partner MMP14 and its regulatory transcriptional factor SMAD2/3 were identified and validated in different pathological models as well as *SMAD2* ^-/-^ mice.

**Results:**

The expression of FBXO6 decreased in the cartilage from human OA samples, anterior cruciate ligament transaction (ACLT) -induced OA samples, spontaneous OA STR/ort samples and aged mice samples. Global knockout or conditional knockout of FBXO6 in cartilage promoted experimental OA process. The molecular mechanism study revealed that FBXO6 decreased MMP14 by ubiquitination and degradation, leading to inhibited proteolytic activation of MMP13. Interestingly, FBXO6 expression is regulated by transforming growth factor β (TGFβ)-SMAD2/3 signalling pathway. Therefore, the overexpression of FBXO6 protected mice from post-injury OA development.

**Conclusions:**

TGFβ-SMAD2/3 signalling pathway suppressed MMP13 activation by upregulating of FBXO6 transcription and consequently promoting MMP14 proteasomal degradation. Inducement of FBXO6 expression in OA cartilage might provide a promising OA therapeutic strategy.

**Key messages**

**What is already known about this subject?**

- Ubiquitination and the proteasome play a role in osteoarthritis (OA) development.
- OA, chondrocytes, FBXO6, MMP14, TGFβ.
- FBXO6, a component of the ubiquitin E3 ligases, is shown to bind high mannose N-linked glycoproteins.

**What does this study add?**

- Our study indicated that FBXO6 suppresses MMP13 activation by regulating MMP14 ubiquitination and degradation.
- Our study demonstrated that TGFβ-SMAD2/3 signalling pathway induces FBXO6 gene transcription and therefore protects cartilage by enhancing FBXO6-mediated MMP14 ubiquitination.

**How might this impact on clinical practice or future developments?**

- TGFβ-FBOX6-MMP14 axis is a potential target for the treatment of OA.

## Introduction

Osteoarthritis (OA) is characterised by progressive degradation of articular cartilage, subchondral bone thickening and osteophyte formation. OA results in substantial morbidity and disability in the elderly, and imposes a great economic burden on society.^1 2^ Despite high prevalence and societal impact, OA’s aetiology and pathogenesis remain elusive.

Studies have provided evidence that transforming growth factor β (TGFβ) can stimulate type II collagen and proteoglycan synthesis^3^ and therefore is critical for cartilage anabolic processes. TGFβ-SMAD2/3 pathway can protect chondrocyte from hypertrophy and degeneration during OA development.^4^ TGFβ has been shown to prevent the loss of proteoglycan in cartilage during experimental OA.^5 6^ Lack of TGFβ causes a reduction in extracellular matrix(ECM) deposition. Consistently, transgenic mice overexpressing the dominant-negative type II TGFβ receptor (*dnTgfbr2*) in skeletal tissue or the conditional deletion of *Tgfbr2* specifically in chondrocytes of adult mice displays progressive skeletal degeneration that strongly resembles human OA.^7 8^

In contrast, catabolic factors such as matrix metalloproteinases (MMPs) are mediated by inflammatory pathways such as mitogen-activated protein kinase (MAPK) pathways or nuclear transcription factor-κB (NF-κB) signalling pathway, which are triggered by inflammatory factor such as interleukin (IL)-1β and tumour necrosis factor (TNF)-α.^9 10^ Most MMPs are secreted into ECM as inactive pro-proteins and activated when pro-peptide cleaved by extracellular proteinases. Membrane-type I matrix metalloproteinase (MT1-MMP or MMP14) is expressed on chondrocyte cell membrane and has been revealed to activate pro-MMP13 and pro-MMP2 by cleaving N-terminal domain.^11 12^ Among them, MMP13 is the principal protease responsible for type II collagen degradation. Besides, MMP14 also has the capacity to degrade ECM components directly.^13^

In the secretory pathway, most proteins are modified with N-glycans in the endoplasmic reticulum(ER),^14^ such as membrane proteins like MMP14 and secretory proteins like other MMPs. F-box protein family, a component of the Skp1-cullin-Fbox ubiquitin E3 ligases, plays important roles in the ubiquitin-proteasome protein-degradation pathway. There are five F-box proteins—FBXO2, FBXO6, FBXO44, FBXO17 and FBXO27—predicted to bind glycoprotein substrates through their conserved C-terminal domain, known as the FBA domain.^15 16^

In our preliminary findings, FBXO6 is upregulated on TGFβ stimulation and is downregulated in human OA cartilage samples. Also in our previous analysis of high performance liquid chromatography/mass spectrometry,^17^ MMP14 was found to specifically bind to FBXO6. These preliminary findings suggest that FBXO6 might be an important player in connecting TGFβ-mediated anabolic process and MMP-mediated catabolic process during OA progression. Thus, this study aims to unveil the role of FBXO6 in OA development.

## Methods

Detailed experimental procedures are described in the online supplementary 1 methods and results.

10.1136/annrheumdis-2019-216911.supp1 Supplementary data

## Results

### Decreased expression of FBXO6 in chondrocytes during OA development

**Figure 1:**
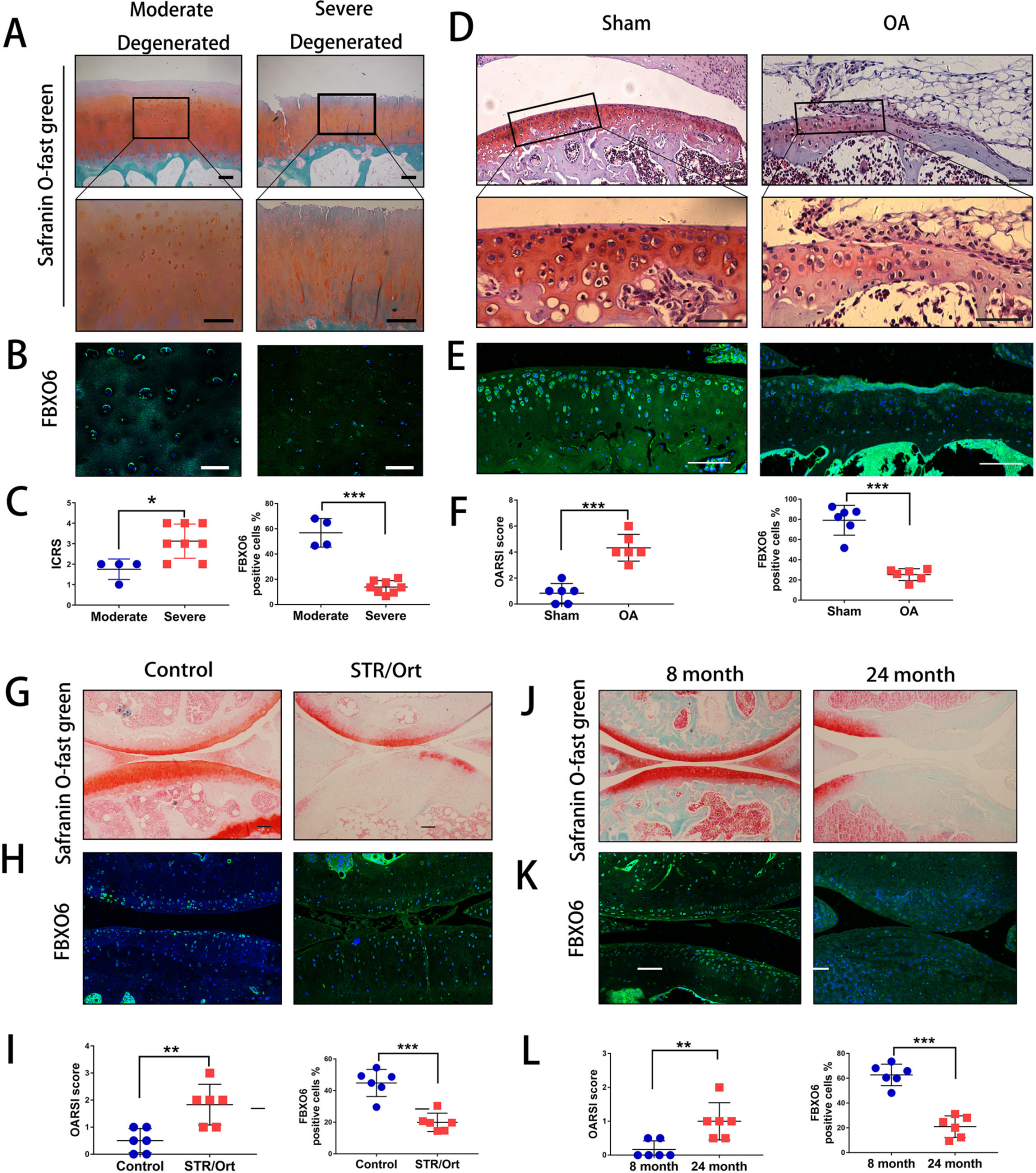
Decreased expression of FBXO6 in chondrocytes during osteoarthritis (AO) development. (A) Safranin O-Fast Green staining in human moderate and severe degenerated cartilage. Insets indicate the regions shown in the enlarged images (down). (B) Immunostaining for FBXO6 in moderate and severe degenerated cartilage. (C) ICRS (International Cartilage Repair Society) scores and quantiﬁcation of FBXO6-positive cells in moderate and severe degenerated cartilage. (D) Safranin O-Fast Green staining in mouse sham and OA tibial cartilage. Insets indicate the regions shown in the enlarged images (down). (E) Immunostaining for FBXO6 in knee joint of sham and OA mouse. (F) Osteoarthritis Research Society International (OARSI) histological scores and quantiﬁcation of FBXO6-positive cells in knee joint of sham and OA mouse. (G) Safranin O-Fast Green staining in control and SRT/Ort mouse joints. (H) Immunostaining for FBXO6 in control and SRT/Ort mouse joints. (I) OARSI histological scores and quantiﬁcation of FBXO6-positive cells in knee joint of control and SRT/Ort mouse. (J) Safranin O-Fast Green staining in 8-month and 24-month mouse joints. (K) Immunostaining for FBXO6 in 8-month and 24-month mouse joints. (L) OARSI histological scores and quantiﬁcation of FBXO6-positive cells in 8-month and 24-month mouse joints. (Scale bars: 100 µm. Data are expressed as mean±SD. **p<0.01; ***p<0.001.)

Initially, we harvested cartilage samples from patients with OA. By Safranin O-Fast Green and H&E staining, we found that superficial region of moderately degenerated articular cartilage appeared to be smooth with only slight surface erosions. In contrast, the superficial layer of severely degenerated OA cartilage exhibited cracks and fissures, with abundant fibrillation or cartilage erosion extending down to subchondral bone (figure 1A and online supplementary figure S1A). ICRS (International Cartilage Repair Society) scores were used to confirm the difference of moderate and severe degenerated cartilage (figure 1C). We next examined the endogenous expression of five F-box proteins predicted to bind glycoprotein substrates by quantitative PCR. The results showed that FBXO6 was significantly reduced in severe OA cartilage, while no difference was observed for FBXO2, 17, 27 and 44 between moderate and severe OA cartilage (online supplementary figure S1B). Consistent with this result, fluorescence immunostaining also showed that the population of FBXO6-positive chondrocytes was signiﬁcantly lower in severely degenerated OA cartilage (figure 1B and C). Western blot analysis further confirmed the decreased expression of FBXO6 protein with concomitant decrease in the expression of collagen type II (Col2) in severely degenerated OA cartilages (online supplementary figure S1C).

10.1136/annrheumdis-2019-216911.supp2 Supplementary data

10.1136/annrheumdis-2019-216911.supp3 Supplementary data

We next investigated whether FBXO6 expression in cartilage was consistently altered in different OA mouse models. In the ACLT-induced OA mouse model, OA was confirmed by Safranin O-Fast Green staining and OARSI score (figure 1D). Cartilage from ACLT-induced OA groups exhibited a dramatic reduction in FBXO6 expression compared with sham control (figure 1E). Similar observations were observed in the spontaneous OA in STR/ort mice and aged mice models. Loss of Safranin-O and cartilage erosion with small vertical clefts in the superficial layer were the main morphological changes in STR/ort OA models (figure 1G) and aged OA models (figure 1J), with the population of FBXO6-positive chondrocytes in both OA models significantly reduced when compared with their respective control counterparts (figure 1H, I for STR/ort OA models and figure 1K, L for aged OA models).

### Global and conditional knockout of FBXO6 in chondrocytes accelerated post-injury-induced cartilage degeneration

**Figure 2:**
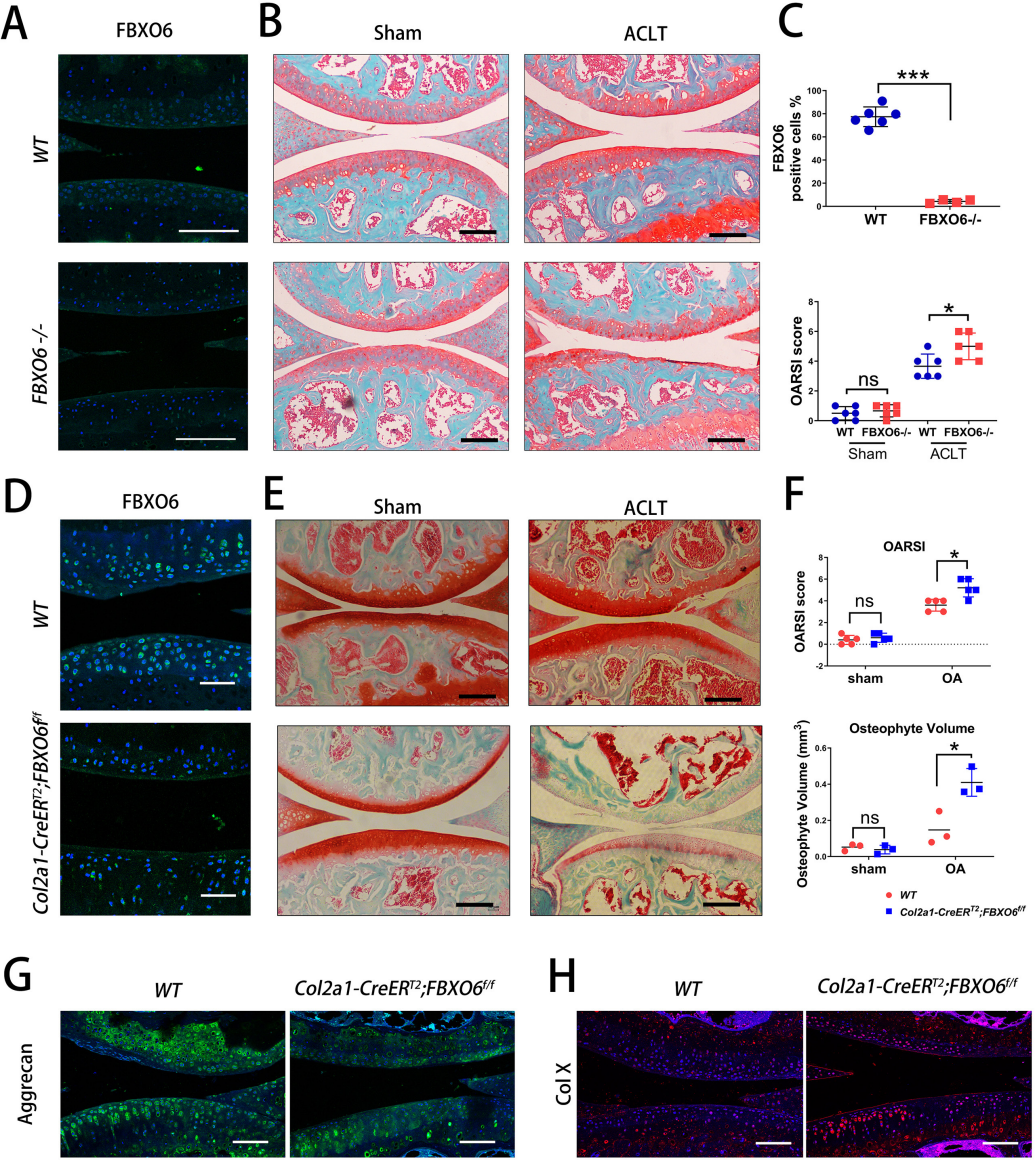
Global knockout and conditional knockout of FBXO6 in chondrocytes accelerated post-injury-induced cartilage degeneration. (A) Immunoﬂuorescence of FBXO6 in global *FBXO6* ^-/-^ mice and wide-type (WT) mice. (B) Safranin O-Fast Green staining of the knee joint at 8 weeks after sham and surgical induction of two genotypes of mice. (C) FBXO6-positive cell quantiﬁcation and OARSI histological scores were compared between global *FBXO6* ^-/-^ mice and WT mice. (D) Immunoﬂuorescence for detection of FBXO6 in *Col2a1-CreER^T2^;FBXO6^f/f^* and WT mice 8 weeks after tamoxifen injection. (E) Safranin O-Fast Green staining of the knee joint at 8 weeks after sham and surgical induction of two genotypes of mice. (F) OARSI (Osteoarthritis Research Society International) histological scores (upper) and osteophyte volume qualification 8 weeks after sham or ACLT surgery (lower) were compared between two genotypes of mice. (G) Immunoﬂuorescence for Aggrecan and Col-X in tibial cartilage at 8 weeks after sham or ACLT surgery. (Scale bars: 100 µm. Data are expressed as mean±SD. *<0.05; ***p<0.001.)

To determine the role of FBXO6 in the pathogenesis of OA, we generated global *FBXO6* ^-/-^ mice and *Col2a1-CreER^T2^:FBXO6^f/f^* inducible conditional knockout mice as described in the Methods section. Knockout efﬁciency was confirmed by ﬂuorescence immunohistochemical staining for FBXO6 in the knee joint cartilage of global knockout mice (figure 2A).

We found that more severe post-injury-induced cartilage degeneration was observed in global knockout mice when compared with WT counterparts at 8 weeks after ACLT surgery, while no morphological differences were observed in the cartilage WT and knockout mice that received sham operation (figure 2B). The effect on subchondral bone was also investigated. Similarly, no ratio of trabecular bone area to total tissue area (B.Ar/T.Ar) or subchondral bone plate (SBP) difference was observed in subchondral bone of WT and global *FBXO6* ^-/-^ mice in sham operation group. Both B.Ar/T.Ar and SBP in ACLT-induced OA group tend to increase in global knockout than in WT mice, though no significant differences were found (online supplementary figure S2A).

10.1136/annrheumdis-2019-216911.supp4 Supplementary data

To further investigate the endogenous *FBXO6* gene in bone and cartilage development, we compared the body weight and length (body and tail) between *global FBXO6^-/-^* and WT mice at 4 or 20 weeks, and found no size or weight difference between the two groups (online supplementary figure S2B). Also, newborn mouse pups of *global FBXO6^-/-^* mice and WT mice were collected, and whole skeletal Alizarin Red and Alcian Blue staining was performed. We found that compared with WT mice, *global FBXO6* ^*-/-*^ mice developed normally with no defects in bone, cartilage or limb (online supplementary figure S2C, D).

Similarly, knockout efﬁciency after tamoxifen administration was examined by staining for FBXO6 (figure 2D) and more severe post-injury-induced cartilage degeneration was found in conditional *FBXO6* ^-/-^ mice than its WT counterparts 8 weeks after ACLT surgery, and similarly, no morphological differences were found in the sham operation group (figure 2E). By micro-CT, no osteophyte formation differences were observed in the cartilage of WT and conditional knockout mice in sham operation group, while increased osteophyte formation was observed in conditional knockout mice in ACLT-induced OA group (online supplementary figure S3A and figure 2F). However, by reconstructed two-dimensional coronal and sagittal images, no significant difference was observed regarding the SBP and BV/TV in conditional *FBXO6* ^-/-^ and WT mice (online supplementary figure S3B, C). Furthermore, the expressions of cartilage ECM component aggrecan and cartilage degeneration marker type X collagen (Col X) were decreased and increased, respectively, further demonstrating the progression of OA in conditional knockout mice (figure 2G).

10.1136/annrheumdis-2019-216911.supp5 Supplementary data

Together, these data suggested loss of FBXO6 accelerated post-injury OA development.

### FBXO6 regulated MMP14 ubiquitination and degradation

**Figure 3:**
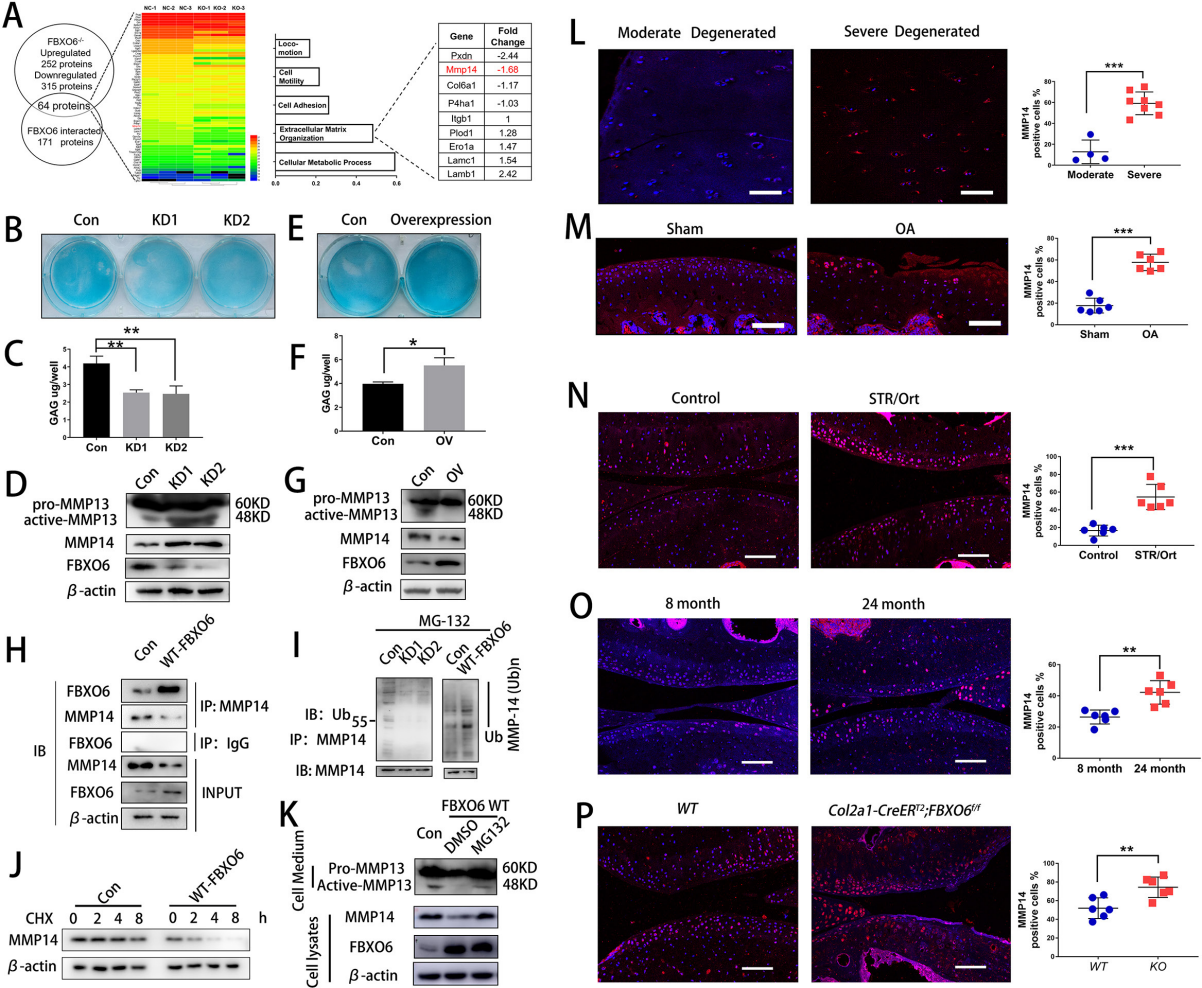
FBXO6 regulates MMP14 ubiquitination and degradation. (A) Comparison of protein expression profiling proteins immunoprecipitated with FBXO6 in HEK293T cell and significantly changed proteins in murine FBXO6 knockout chondrocytes, and possible biological process associated with the proteins using Gene Ontology (GO) analysis. (B) Alcian blue staining and (C) DMMB assays to examine the effects of FBXO6 knockdown on glycosaminoglycan expression in ATDC5 cells. (D) Western blotting detected the protein level of active-MMP13 in the cell medium and MMP14 after FBXO6 knockdown. (E) Alcian blue staining and (F) DMMB assays to examine the effects of FBXO6 overexpression on glycosaminoglycan expression in ATDC5 cells. (G) Western blotting detected the protein level of active-MMP13 in the cell medium and MMP14 after FBXO6 overexpression. (H) IP detected the binding of FBXO6 and MMP14. (I) Western blotting detected the ubiquitination level of MMP14 in FBXO6 overexpression and FBXO6 knockdown ATDC5 cells. (J) Western blot compared the degradation rate of MMP14 in control and FBXO6 overexpression of ATDC5 after being treated with CHX. (K) Western blotting detected FBXO6-induced MMP14 degradation after administration of proteasome inhibitor MG132. (L) Immunostaining and quantiﬁcation of MMP14-positive cells in moderate and severe degenerated cartilage (M), knee joint of sham and OA mouse (N), control and SRT/Ort mouse (O), 8-month and 24-month mice (P) and *Col2a1-CreER^T2^;FBXO6^f/f^* and wide-type (WT) mice. (Data are expressed as mean±SD. *p<0.05; **p<0.01; ***p<0.001.) CHX, cyclohexamide; IgG, immunoglobulin G; IP, immunoprecipitation; MMP, matrix metalloproteinase; OA, osteoarthritis; WT, wild type.

To identify the underlying mechanisms of FBXO6 on OA development, the differential expression of proteins in murine FBXO6 knockout chondrocytes was investigated using mass spectrometry. In all, 252 proteins showed upregulated expression and 315 proteins showed downregulated expression in murine FBXO6 knockout chondrocytes as compared with WT chondrocytes (online supplementary 2). The protein expression profiling was investigated via immunoprecipitation with FBXO6 antibody in HEK293T cell, and a total of 171 proteins were extracted to interact with FBXO6 (online supplementary 3). Finally, 64 proteins were selected from the overlap between Supplementary 2 and Supplementary 3 (figure 3A). By performing Gene Ontology (GO) analysis, we found that overlapping proteins were mainly associated with cellular metabolic process, extracellular matrix organisation, cell adhesion and so on (figure 3A and online supplementary 4). MMP14 was one of the most increased proteins in extracellular matrix organisation subgroup in murine FBXO6 knockout chondrocytes.

10.1136/annrheumdis-2019-216911.supp7 Supplementary data

10.1136/annrheumdis-2019-216911.supp8 Supplementary data

**Figure 4:**
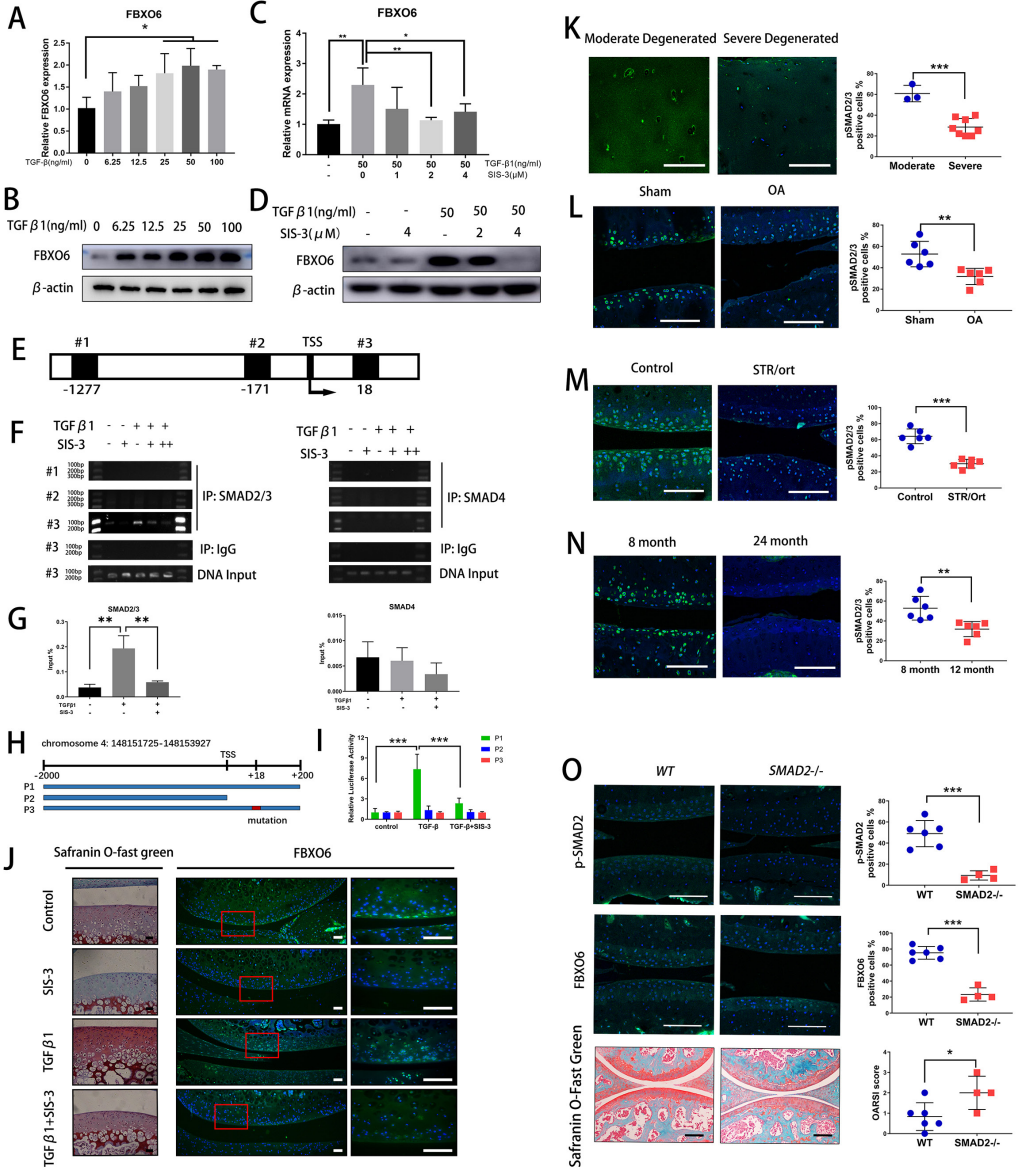
TGFβ-SMAD2/3 signalling pathway induced FBXO6 gene transcription. (A) Quantitative real-time PCR (qRT-PCR) was performed to validate the expression of FBXO6 after 0–100 ng/mL of TGF-β was added to ATDC5 cells. (B) Western blot analysis of the protein levels of FBXO6 after TGFβ was added to ATDC5 cells. (C) qRT-PCR was performed after SMAD3 phosphorylation inhibitor SIS-3 was added to ATDC5 cells. (D) Western blot analysis of the change of FBXO6 protein levels after SIS-3 was added to ATDC5 cells. (E) Schematic diagram of three putative FBXO6-binding sites on FBXO6 promoter. (F) Chromatin immunoprecipitation analysis was performed using a negative control immunoglobulin G (IgG) and anti-SMAD2/3 antibody or SMAD4 antibody in ATDC5 cells. (G) qPCR of the immunoprecipitated SMAD2/3 or SMAD4 bind DNA and input DNA after being stimulated with TGFβ and SIS-3. (H) Schematic diagram depicting the wild type, lack type and mutant type of luciferase reporter gene. (I) Analysis of luciferase activity of three reporter genes after being stimulated with TGFβ and SIS-3 in ATDC5 cells. (J) Safranin O-Fast Green staining and immunoﬂuorescence for FBXO6 after TGFβ and SIS-3 added in rat knee joint cultured ex vivo. Insets indicate the regions shown in the enlarged images (right). (Scale bars, 50 µm). (K) Immunostaining for pSMAD2/3 in moderate and severe degenerated cartilage. Immunostaining for pSMAD2/3 in knee joint of OA mouse (L), spontaneous osteoarthritis STR/Ort mouse (M) and aged mouse (N). (O) Immunoﬂuorescence of p-SMAD2 and FBXO6 and Safranin O-Fast Green staining in *global SMAD2* ^*-/-*^ mice and wide-type (WT) mice knee joint. p-SMAD2, FBXO6-positive cells and OARSI histological score quantiﬁcation were compared between two groups of mice. (Scale bars: 100 µm. Data are expressed as mean±SD. *p<0.05; **p<0.01; ***p<0.001.) IgG, immunoglobulin G; IP, immunoprecipitation; OA, osteoarthritis; WT, wild type.

In order to decipher the underlying mechanism for the effects of FBXO6 on MMP14 and cartilage homeostasis, ATDC5 cells were transduced with either FBXO6 overexpression or shRNA knockdown lentiviral particles. Knockdown of FBXO6 significantly reduced the glycosaminoglycan (GAG) content deposited in the ECM evidenced by Alcian blue staining (figure 3B) and extracted from ECM by dimethylmethylene blue (DMMB) assays (figure 3C). Overexpression of FBXO6 exerted opposite effects by increasing the GAG contents in the ECM (figure 3E). By immunoblot analyses, the inverse relationship was again observed between FBXO6 and MMP14 or MMP13. That is, FBXO6 knockdown results in upregulation of MMP14 and activated MMP13 (figure 3D), whereas FBXO6 overexpression downregulated the expression of MMP14 and inhibited the activation of pro-MMP13 (figure 4G), as MM14 activated pro-MMP13 in the medium directly (online supplementary figure S4A). Similar to the result in chondrogenic ATDC5 cell line, we have found that in FBXO6^-/-^ chondrocytes, MMP14 was upregulated (online supplementary figure S4B). By co-immunoprecipitation, we confirmed that FBXO6 could bind to MMP14 in both ATDC5 (figure 3H) and SW1353 chondrosarcoma cell line (online supplementary figure S4C). By immunofluorescence staining, we showed that overexpression of FBXO6 inhibited the expression of MMP14, whereas FBXO6 knockdown increased MMP14 expression (online supplementary figure S4D). Consistent with this, we also found that FBXO6 overexpression or knockdown significantly enhanced or abolished MMP14 ubiquitination, respectively (figure 3I). Interestingly, overexpression of FBXO6 induced the time-dependent degradation MMP14 when de novo protein synthesis was inhibited by cyclohexamide (figure 3J). Treatment of cells with proteasome inhibitor, MG132, attenuated FBXO6-induced MMP14 degradation (figure 3K). Furthermore, using tunicamycin to block N-link glycosylation prevented MMP14 degradation (online supplementary figure S4E). Taken together, these data provided evidence that FBXO6 regulated MMP14 ubiquitination and degradation, which, in turn, affects the proteolytic activation of pro-MMP13.

10.1136/annrheumdis-2019-216911.supp9 Supplementary data

The correlation between FBXO6 and MMP14 was also confirmed in different in vivo models. We examined the expression of MMP14 in human OA cartilage and various OA mouse models. In accordance with the decreased FBXO6 expression, fluorescence immunostaining showed that the population of MMP14-positive chondrocytes was signiﬁcantly elevated in human severe degenerated OA cartilage (figure 3L), ACLT-induced OA mice (figure 3M), spontaneous OA in STR/Ort mice (figure 3N), 24-month aged mice (figure 3O) and *Col2a1-CreER^T2^:FBXO6^f/f^* conditional knockout mice (figure 3P).

### TGFβ-SMAD2/3 signalling pathway induced FBXO6 gene transcription

We then asked why the expression of FBXO6 was downregulated during OA development. Interestingly, we noticed the gene and protein expression of FBXO6 were dose-dependently increased by TGFβ stimulation in chondrogenic ATDC5 cell line (figure 4A, B). ATDC5 cells were treated with SIS-3 which selectively inhibits TGFβ signalling by suppressing SMAD3 phosphorylation. As shown in figure 4C, D, the induced gene and protein expression of FBXO6 in response to TGFβ was abrogated by SIS-3, confirming the involvement of TGFβ-SMAD2/3 signalling in FBXO6 induction in vitro.

We next examined transcriptional regulation of FBXO6 by SMAD2/3 in chondrogenic ATDC5 cell line. Three putative SMAD2/3/4-binding elements were identified in the FBXO6 promoter: #1 at −1277 to −1263, #2 at −171 to −257 and #3 at +18 to +30 (figure 4E). By chromatin immunoprecipitation, we observed a dramatic increase in SMAD2/3 binding to the #3 putative SMAD2/3-binding element after TGFβ stimulation and this binding was abrogated with the presence of SIS-3. No binding was observed from DNA precipitated by the isotype-matched control IgG or with SMAD4 antibodies (figure 4F, G). To confirm the functional link between the SMAD2/3-binding element on the FBXO6 promoter, luciferase-based reporter assays were performed. Three FBXO6 promoter luciferase reporters were designed and synthesised: P1, WT −2 kB FBXO6 promoter containing the #3 SMAD2/3-binding element; P2, null FBXO6 promoter lacking the #3 SMAD2/3-binding element; and P3, mutant FBXO6 promoter with mutated #3 SMAD2/3-binding element (figure 4H). As expected, TGFβ stimulation increased transcriptional activity of the WT FBXO6 promoter reporter construct, but not that of the null or mutated FBXO6 promoter reporters. Again, SIS-3 treatment markedly inhibited the TGFβ-induced transcriptional activity of WT FBXO6 promoter reporter (figure 4I). Using an ex vivo explant culture of rat knee joint, we further confirmed that the elevated cartilage ECM deposition and FBXO6 expression in response to TGFβ were decreased after being treated with SIS-3 (figure 4J). Interestingly, we also found that pSMAD2/3 was also decreased in human severely degenerated OA cartilage (figure 4K). We further confirmed the downregulation of the SMAD2/3 pathway in OA chondrocytes in three mouse models of OA. The population of pSMAD2/3-positive chondrocytes were significantly lower in ACLT-induced OA mice (figure 4L), in the spontaneous OA STR/Ort mouse (figure 4M) and in the aged mouse (figure 4N) when compared with their respective control mice. *SMAD2* ^*-/-*^ mice were used to verify results presented above. Knockout efﬁciency was examined by ﬂuorescence immunohistochemical staining for p-SMAD2 in the knee joint cartilage of *SMAD2* ^*-/-*^ mice and a dramatic reduction in FBXO6 expression was observed compared with wide-type (WT) controls. Besides, the vertical clefts below the superficial layer of cartilage were found in *SMAD2* ^*-/-*^ mice, and OARSI scoring was confirmed for the degeneration of the cartilage in the *SMAD2* ^*-/-*^ mice (figure 4O). Collectively, these results provided evidence for a strong association between decreased FBXO6 expression and downregulation of TGFβ-SMAD2/3 pathway during OA progression.

### Overexpression of FBXO6 in cartilage alleviates OA in mice

**Figure 5:**
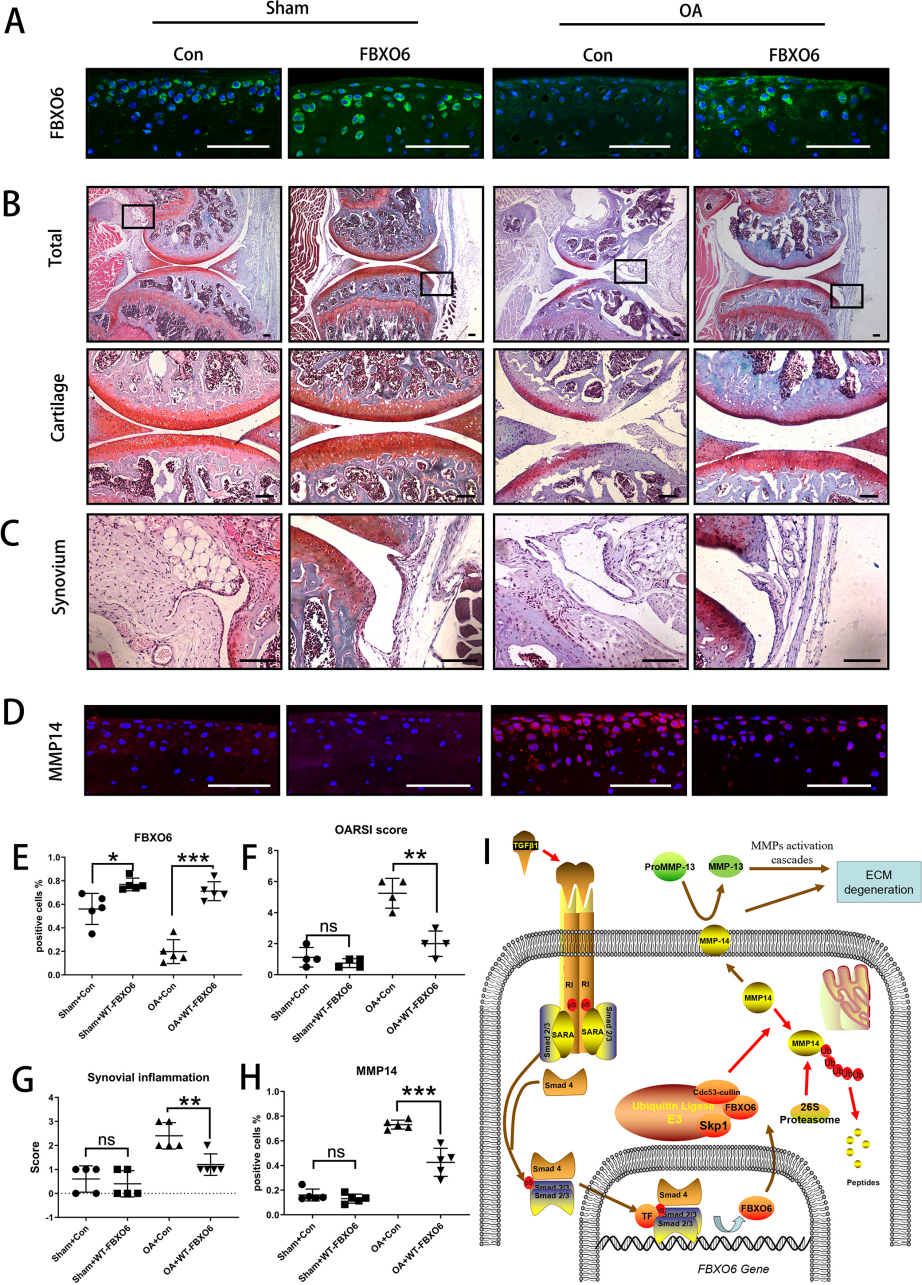
Overexpression of FBXO6 in cartilage induces alleviated osteoarthritis in mice. (A) Immunostaining of FBXO6 in sham and ACLT joint sections of mice intra-articular injected with LV-Con or LV-FBXO6, (B) Safranin O-Fast Green staining of sham and ACLT joint sections. Insets indicate the regions shown in the enlarged images (down). Synovium (C) and immunostaining of MMP14 (D) in sham and ACLT joint sections. (E) Quantiﬁcation of FBXO6-positive cells in sham and ACLT joint sections. (F) OARSI histological scores in sham and ACLT joint sections. (G) Synovial inﬂammation score in sham and ACLT joint sections. (H) Quantiﬁcation of MMP14-positive cells in sham and ACLT joint sections. (I) Schematic of role of TGFβ-FBOX6-MMP14-active MMP13 pathway in the cartilage. (Scale bars: 100 µm. Data are expressed as mean±SD. *p<0.05; **p<0.01; ***p<0.001.) ECM, extracellular matrix; MMP, matrix metalloproteinase; OA, osteoarthritis; OARSI, OsteoarthritisResearch Society International.

Finally, we investigated whether the overexpression of FBXO6 could protect against the progression of OA in vivo. Intra-articular injection of WT FBXO6 (LV-FBXO6) lentiviral particles into mouse knee joints after ACLT surgery. Efﬁciency of lentiviral infection in the knee joint was assessed by green ﬂuorescence protein (online supplementary figure S7A). Both sham-operated and ACLT-operated OA groups infected with FBXO6 lentiviral particles showed higher protein expression in articular chondrocytes than respective counterparts infected with control virus further attesting to the efficient delivery of FBXO6 to the cells in the cartilage (figure 5A). More importantly, in contrast to control-treated animals which demonstrated severe cartilage erosion and synovial inflammation, overexpression of FBXO6 into the knee joint effectively alleviated the cartilage destruction associated with ACLT surgery (figure 5B) as well as significantly reducing the severity of synovial inﬂammation (figure 5C). Besides, no B.Ar/T.Ar or SBP difference was observed in subchondral bone of LV-Con and LV-FBXO6 mice in sham operation group. However, SBP in ACLT-induced OA group was thinner in LV-FBXO6 than in LV-Con mice, although the difference was not significant (online supplementary figure S7B). Furthermore, the upregulation of MMP14 observed in the cartilage tissue from control OA mice was also inhibited in the following FBXO6 overexpression (figure 5D, H). Together, these results provided promising evidence that the targeted expression of FBXO6 in the cartilage tissue might represent an effective therapeutic option for the prevention of OA. Based on these results, we proposed that TGFβ-SMAD2/3-FBXO6 is important for the development of OA (figure 5I).

10.1136/annrheumdis-2019-216911.supp10 Supplementary data

## Discussion

The pathogenesis of OA is complex, and its specific pathological mechanism remains to be fully elucidated. In this study, we found that FBXO6 expression and SMAD2/3 signalling pathway were downregulated in severely degenerated human OA cartilage and in various murine models of OA. Using FBXO6 conditional knockout mice and global FBXO6^-/-^ mice, we demonstrated that the loss of FBXO6 accelerated post-injury-induced cartilage destruction without significantly influencing subchondral bone and osteophyte formation. By in vitro analysis, we showed that FBXO6 gene expression was under transcriptional control of TGFβ-SMAD2/3 signalling. Meanwhile, we found that the downregulation of FBXO6 was associated with the concomitant increase in the expression of MMP14 and activation of soluble MMP13. Furthermore, overexpression of FBXO6 resulted in MMP14 ubiquitylation targeting it for proteasomal degradation. Additionally, overexpression of FBXO6 was found to protect mice against ACLT-induced OA by downregulating MMP14. Based on these results, we proposed that TGFβ-SMAD2/3-FBXO6 is important for the maintenance of cartilage homeostasis.

TGFβ has different roles in various components of joint tissues, such as subchondral bone or synovial fibroblasts.^18–20^ Also, it plays different roles in chondrocytes according to its downstream signalling pathway.^3^ Two kinds of TGFβ type I receptors, or activin receptor-like kinases (ALKs), are involved in chondrocytes, ALK1 and ALK5. To our knowledge, ALK5 stimulates the phosphorylation of SMAD2 and SMAD3, while ALK1 mediates the activation of SMAD1, SMAD5 and SMAD8. However, two main intracellular SMAD pathways are found to antagonise each other: ALK1-SMAD1/5/8 signalling stimulates chondrocyte hypertrophy, whereas ALK5-SMAD2/3 signalling stimulates type II collagen and proteoglycan synthesis and blocks chondrocyte hypertrophy.^3^ For the cartilages of ageing and OA mouse models, ALK5 expression was decreased much more than ALK1, thus the ALK1/ALK5 ratio was increased.^3^ Thus, TGFβ-SMAD2/3 signalling pathway is generally thought to be beneficial for chondrocytes, although the residual TGFβ level in normal or degenerated cartilage remains disputable.^21–24^ Consistent with previous studies, we revealed that SMAD2/3 signalling pathway was decreased in cartilages of different OA models and degenerated human OA cartilages.

Apart from enhancing the anabolic process in the cartilage, TGFβ-SMAD2/3 signalling pathway also modulates other signalling pathway activated by inflammation in OA. As an immune suppressor, TGFβ1 has been shown to inhibit NF-κB-dependent inflammatory responses.^25^ Azumaet *et al* found that TGFβ1 downregulates NF-κB activity through the induction of IκB-α expression.^26^ While Cavin *et al* found that TGFβ1 could induce of stabilisation of IκB-α protein by inhibiting protein kinase CK2 expression, which regulates IκB-α protein turnover.^27^ Hence, TGFβ is regarded to reduce the MMPs translation induced by inflammatory cytokines in the cartilage. In our study, we found that TGFβ-SMAD2/3-FBOX6-MMP14 was a novel ‘crosstalk’ between TGFβ anabolic processes and MMP catabolic processes in human OA progress.

Recently, it is suggested that proteasome ubiquitination plays a role in arthritis diseases.^28^ Proteasome inhibitor MG132^29^ or Ub K48R mutation^30^ protected the cartilage from cytokine-mediated degradation and against DMM-induced OA in mice. FBXO6, one of the five F-box proteins predicted to bind glycoprotein substrates through FBA domain, is shown to bind high mannose N-linked glycoproteins and functions as ubiquitin ligase subunits.^17^ Most proteins in the secretory pathway, such as membrane proteins and secretory proteins and MMPs, are modified with N-glycans in the ER.^14^ MMP14 is found to contain two potential N-glycosylated sequences at Asn^229^ (catalytic domain) and Asn^311^ (linker domain),^31^ and is a potential degradation protein target of FBXO6. In this study, we found that FBXO6 expression in cartilage were reduced in both human and mouse OA models and proved that MMP14 bind to FBXO6 FBA domain through its N-linked glycoproteins. We found that MMP14 was increased in human OA cartilages and in various murine OA models in the opposite pattern of FBXO6 expression and is related to the severity of OA. Previous reports have suggested that MMP14 proteolytically activates MMP2 and MMP13.^11 12^ Consistent with these previous studies, we found that downregulation of FBXO6 which concomitantly increases MMP14 expression led to elevated proteolytic cleave of inactive pro-MMP13 to active MMP13. However, proteolytic removal of pro-peptides was done by many proteases apart from MMP14, such as plasmin, meprins, furins or other activated MMPs.^31^ Besides, MMP was also demonstrated to cleave within cells in nuclear, mitochondrial or cytoplasmic compartments.^32^ However, in this study, the intracellular activation of the MMP13 and other proteases attended extracellularly in the MMP activation process were not fully assessed. Collectively, we propose that MMP14 expression is negatively controlled by TGFβ-SMAD2/3-FBXO6 signalling axis.

The effects of FBXO6 on subchondral bone and osteophyte formation were also evaluated. Although more studies in OA have primarily focused on articular cartilage, researchers recently considered the subchondral bone and articular cartilage act as a functional unit in the joint.^33^ Subchondral bone sclerosis is a crucial factor for OA as subchondral bone provided mechanical support for overlying articular cartilage, as subchondral bone sclerosis leads to instability of mechanical force on cartilage and thus cause its erosion.^20^ Here we studied the effect of FBXO6 on bone. We found no difference in bone development of newborn *global FBXO6* ^*-/-*^ and WT mice. Moreover, both the conditional knockout and the global FBXO6 knockout mice of the OA model exhibited similar subchondral bone sclerosis and osteophyte formation compared with their WT littermates. We also generated *prx1-Cre: FBXO6* ^*f/f*^ mice and found no significant change in bone mass compared with WT littermates (unpublished data). Although TGFβ-ALK-SMAD signalling pathway is well recognised in both subchondral bone and articular cartilage,^34^ our data suggested its downstream protein FBXO6 may not be involved during subchondral sclerosis or osteophyte formation. We suppose FBXO6 mainly functions in chondrocytes during OA development. Besides, the effects of FBXO6 on osteoclast will also be investigated in our further studies.

In conclusion, we found that SMAD2/3-FBXO6 axis was downregulated, while MMP14 and active MMP13 were upregulated in OA. We provided several aspects of evidence for TGFβ-SMAD2/3 signalling pathway in the regulation of FBXO6 expression and FBXO6 interaction with MMP14, leading to its ubiquitylation and proteasomal degradation while reduced MMP14 inhibited the proteolytic activation of MMP13. Thus, inducing FBXO6 expression to reduce the catabolic effects of MMP14/active MMP13 in OA cartilage may provide a novel approach in the therapeutic treatment of OA.

10.1136/annrheumdis-2019-216911.supp6 Supplementary data

10.1136/annrheumdis-2019-216911.supp11 Supplementary data

10.1136/annrheumdis-2019-216911.supp12 Supplementary data

10.1136/annrheumdis-2019-216911.supp13 Supplementary data
