# Supplementary material for: TGFβ attenuates cartilage extracellular matrix degradation via enhancing FBXO6-mediated MMP14 ubiquitination
Source: Ann Rheum Dis. 2020 May 14;79(8):1111–20. doi: 10.1136/annrheumdis-2019-216911 (PMC7392491; doi:10.1136/annrheumdis-2019-216911)
Supplement: Supplementary data [file annrheumdis-2019-216911supp001.pdf]

Methods

1. Human cartilage acquisition

Cartilage samples were collected following informed written patient consent with approval from Sir Run Run Shaw Hospital Ethics Committee. Cartilages were excised from femoral heads collected from patients undergoing total hip or knee replacement surgery (detail listed in Table 1). Severe OA cartilage explants were harvested from 8 patients (6 females and 2 males with mean age of 75 years) undergoing total hip or knee replacements due to end-stage OA. Moderate OA cartilage explants were harvested from 4 patients (3 females and 1 male with mean age of 59 years) undergoing hip replacement due to femoral neck fracture. Cartilage samples were used for subsequent RNA and protein extraction, histological assessment, and immunostaining.

This research was done without patient involvement. Patients were not invited to comment on the study design and were not consulted to develop patient relevant outcomes or interpret the results. Patients were not invited to contribute to the writing or editing of this document for readability or accuracy.

Table 1.

Patient Nº.	Diagnosis	Gender	Age
1	Osteoarthritis (right knee)	Female	70
2	Osteoarthritis (right knee)	Female	81
3	Osteoarthritis (right femur)	Male	75
4	Osteoarthritis (left femur)	Female	75
5	Osteoarthritis (right knee)	Female	66
6	Fracture (left femur)	Male	54
7	Osteoarthritis (right femur)	Female	74
8	Fracture (right femur)	Female	53
9	Osteoarthritis (right femur)	Male	82
10	Fracture (left femur)	Female	62
11	Fracture (right femur)	Female	65
12	Osteoarthritis (right knee)	Female	77

2. RNA extraction and real-time quantitative polymerase chain reaction (qPCR)

For the extraction of total tissue RNA from human cartilage samples, 50mg of cartilage tissue were mechanically minced then snap frozen using liquid nitrogen and grounded into a fine powder using a mortar and pestle, and then total RNA extracted using TRIzol RNA extraction reagent (CWBIO, Beijing, China) in accordance with manufacturer's protocol. Complementary DNA (cDNA) was then reverse transcribed using 1.0µg of extracted RNA and HiFiScript cDNA Kit (CWBIO, China) following the guidelines of the manufacturer. Real-time qPCR was performed on the ABI Prism 7500 system (Applied Biosystems, Foster City, CA, USA) using the UltraSYBR qPCR Mixture (CWBIO, China) according to manufacturer's protocol and carried out using the following cycling conditions: 95°C for 10mins (activation); followed by 40 cycles at 95°C for 10secs, 60°C for 20secs, and 72°C for 20secs (amplification); and a final extension at 72°C for 60secs. Specific forward and

reverse primers based on human and mouse sequences are listed in Supplementary Table 1 and were purchased from TSINGKE Biological Technology (Shanghai, China). Each qPCR was performed 3 times with at least 3 different experimental replicates, and results were normalized to the gene expression 18S.

3. Human cartilage protein extraction and western blot analysis

Human cartilage tissues were first dissected into smaller pieces of approximately 0.5mm×0.5mm in size and then homogenized in 200μl RIPA lysis buffer supplemented with protease inhibitor cocktail (Sigma-Aldrich, St. Louis, MO, USA). The homogenates were then centrifuged at 12000 rpm and the resulting supernatants were collected. Proteins were resolved on 10% SDS-PAGE gels and separated proteins then transferred to PVDF membranes then blocked in 5% (w/v) skim milk powder in TBST (Tris-buffered saline with 0.1% Tween-20) for 1hr at room temperature and then incubated with anti-FBXO6 (ab153853; Abcam, Cambridge, UK), MMP13 (ab39012; Abcam), MMP14 (ab51074; Abcam), collagen II (ab34712; Abcam) or β-actin (sc-47778; Santa Cruz Biotechnology, Dallas, TX, USA) diluted 1:1000 in 1% (w/v) skim milk powder in TBST overnight at 4°C. After extensive washes in TBST, membranes were incubated with appropriate horseradish peroxidase (HRP)−conjugated secondary antibodies diluted 1:1000 in 1% (w/v) skim milk powder in TBST for 1hr at room temperature. Excess secondary antibodies were rinsed off with TBST, and chemiluminescence signals were visualized following exposure to electrochemical luminescence reagent (Millipore Sigma, Burlington, MA, USA) and imaged on a LAS-4000 Science Imaging System (Fujifilm, Tokyo, Japan).

4. Histological and immunohistochemical analyses of human cartilage

Human cartilage samples were fixed in 4% paraformaldehyde (PFA), decalcified in 0.5M EDTA for 5 days, and then embedded in paraffin and sectioned continuously (7μm thick). Representative sections were deparaffinized in xylene, hydrated with graded ethanol, and then stained with Hematoxylin & Eosin (H&E) or Safranin O-Fast Green. The International Cartilage Repair Society (ICRS) cartilage lesion classification system was used to assess the human OA samples as described previously [1]. The sections were mounted and imaged using a Nikon ECLIPSE 80i microscope (Nikon Instruments, Tokyo, Japan). For immunofluorescence, deparaffinized and rehydrated sections were soaked in solution of 0.05% trypsin in 0.1% $CaCl_2$ (pH 7.8) for 30mins at 37°C for antigen retrieval. After blocking with 1% bovine serum albumin (BSA) in phosphate-buffered saline (PBS) at room temperature for 1hr, sections were incubated with specific primary antibodies (diluted 1:500 in 5% BSA) against p-SMAD2/3 (D27F4; Cell Signaling Technology, Danvers, MA, USA), FBXO6 (ab153853; Abcam), or MMP14 (ab78738; Abcam) at 4°C overnight. Sections were then washed extensively with PBS and incubated with Alex Fluor 488-conjugated goat anti-rabbit or Alex Fluor 594-conjugated goat anti-mouse secondary antibodies (1:200 in 5% BSA) (Thermo Fisher Scientific) for 1hr at room temperature. Nuclei were counterstained with DAPI (Beyotime Institute of Biotechnology, Jiangsu, China) before imaging using an Olympus BX51 fluorescence microscope (Olympus Life Science, Tokyo, Japan).

5. *Col2a1-CreER^{T2}:FBXO6$^{f/f}$* conditional knockout (cKO) mice; Spontaneous OA STR/ort and aged mice; and ACLT-induced OA C57BL/6 mice

All experimental procedures involving mice were conducted with the approval of the Zhejiang University Ethics Committee. Homozgous *FBXO6* floxed mice *(FBXO6$^{f/f}$)* mice, global *FBXO6$^{-/-}$* and the transgenic mouse line expressing the Cre recombinase under the control of the type II collagen promoter and inducible by tamoxifen administration *(Col2a1-CreER^{T2})* were kindly provided by the laboratories of Ruijin Hospital, Shanghai Jiao Tong University School of Medicine. Both mice were of the C57BL/6 background. To generate *Col2a1-CreER^{T2}:FBXO6$^{f/f}$* mice (n = 10), *FBXO6$^{f/f}$* mice were bred with *Col2a1-CreER^{T2}* transgenic mice to obtain *Col2a1-CreER^{T2}:FBXO6$^{f/-}$* heterozygous mice, which were then mated with homozygous *FBXO6$^{f/f}$* mice. Age-matched WT littermates were used as controls (n = 10). Before surgical induction of OA, tamoxifen (TM) (dissolved in corn oil at concentration of 100μg/g body weight; Sigma-Aldrich) was intraperitoneally injected into 8-week-old *Col2a1-CreER^{T2}:FBXO6$^{f/f}$* mice daily for 5 consecutive days. Four weeks after TM administration, *Col2a1-CreER^{T2}:FBXO6$^{f/f}$* mice underwent anterior cruciate ligament transection (ACLT) surgery to the left knees and sham surgery on the right knees as described previously [2]. In brief, mice were placed under general anesthesia with 3% isoflurane, and hindlimbs were shaved and prepared for aseptic surgery. For sham operation, the knee joint was exposed following medial capsular incision and the fat pad was dissected and then closed with sutures. For ACLT surgery, after exposing the joint capsule, the ACL was transected with micro-scissors under a surgical microscope. After irrigation with saline to remove tissue debris, the skin incision was closed with sutures. Immediately after surgery, the animals were returned to their individual cages without joint immobilization. The mice were sacrificed 8 weeks after sham or ACLT surgery. ACLT surgery was also performed on normal 8-week-old C57BL/6 mice (n = 6) and global *FBXO6$^{-/-}$* mice (n = 6) to the left knee and sham operation to the right knee and sacrificed 8 weeks after surgery.

Knee joints isolated from 40-week-old male STR/Ort mice (kindly donated by Research Centre for Regenerative Medicine, Guangxi Medical University) were analyzed and divided into severe and moderate OA groups according to the OARSI score (n = 6 each). Moderate degenerated STR/ort knee joints were used as controls. Knee joints were also isolated from 8-month-old control mice and 24-month-old aged mice (n = 6 each); 30-week-old global *SMAD2$^{-/-}$* (kindly donated by laboratories of Shanghai institute of traumatology and orthopaedics, n = 4) and age matched mice (n=6).

Knee joints were embedded in paraffin, sectioned and stained with Safranin-O/Fast Green and immunofluorescence staining as described above. Primary antibodies used for immunofluorescence staining include FBXO6 (11830-1-AP; Proteintech, Rosemont, IL, USA), MMP14 (ab78738; Abcam), Aggrecan (ab3778; Abcam), p-SMAD2/3 (D27F4; Cell Signaling Technology) and Collagen II (ab34712; Abcam). Immunofluorescence intensity were quantified using ImageJ Software (1.48, NIH, Bethesda, MD, USA). Histological assessment of sagittal sections of the knee joints was conducted by two blinded observers who followed the Osteoarthritis Research Society International (OARSI) scoring system [3]. Measurements were also performed area on the proximal side of the growth plate as describe previously[4-6], in which B.Ar/T.Ar (T.Ar, total tissue area,

calculated from the total tissue area; and B.Ar, trabecular bone area, calculated from the total trabecular area) and thickness of the medial subchondral bone plate (region between the osteochondral junction and marrow space on the medial side of the tibial plateau, in μm) were measured using Bioquant Osteo software (BIOQUANT, Inc.).

6. Skeletal staining

Whole newborn mouse pups of global *FBXO6*$^{-/-}$ mice and WT mice were skinned, eviscerated, fixed with 95% ethanol for 4 days, and treated with acetone for 3 days. Cartilage was stained with Alcian blue, and skeletal staining performed using a solution containing Alizarin red, as described previously[4].

7. Micro-computed tomography (μCT)

Knee joints excised from *Col2a1-CreER*^{T2}*:FBXO6*$^{f/f}$ mice were scanned using the Skyscan 1072 high-resolution μCT scanner (Bruker, Aartselaar, Belgium) at X-ray voltage of 70kV, current of 80μA and pixel size of 9μm. Three dimensional reconstructions of the knee joints were carried using SkyScan NRecon software with cone beam volumetric algorithm and SkyScan CT Analyzer software (Bruker) was used to quantify osteophyte volume for each sample. Also, the epiphysis of the tibia was manually chosen as the region of interest for 3D analysis of the subchondral bone. For subchondral trabeculae, trabecular bone volume fraction (BV/TV), representing the ratio of trabecular bone volume (BV) to endocortical tissue volume (TV); and the thickness of the subchondral bone plates were calculated, as described previously[5 7 8].

8. Intra-articular delivery of FBXO6 overexpression lentivirus in experimental OA

Intra-articular injections of lentiviral particles were administered 7- and 14-days after ACLT OA induction in 8-week old male C57BL/6 mice as described above. Mice were immobilized in a 50-ml falcon tube (head facing to the bottom of the tube) and the left leg was exposed for lentiviral particles injection. 10μl of concentrated lentiviral particles expressing mouse FBXO6 (LV-FBXO6) or negative control (LV-Con) (GenePharma, Shanghai, China) was injected into the joints using an insulin injection needle. Eight weeks after injection of lentiviral particles, mice were sacrificed, knee joints excised and embedded in paraffin for sectioning. Safranin-O/Fast Green and immunofluorescence staining with anti-GFP (ab183734; Abcam) was carried out as described above. For the assessment of joint synovitis, Safranin-O/Fast Green stained knee joint sections were assessed for the thickness of the synovium. Histological changes were graded on an arbitrary scale from 0 to 3 (0 = no synovial thickening; 1 = lining of two cell layers; 2 = several extra cell layers; 3 = clear inflammation with cell infiltrate or exudate). Scoring was performed by two blinded observers as previously described.

9. Primary culture of global *FBXO6*$^{-/-}$ mice articular chondrocytes and chondrocytic ATDC5 cells

The chondrogenic ATDC5 cell line was purchased from Riken Cell Bank (Ibaraki, Japan) and maintained in DMEM supplemented with 10% FBS, and 100U/ml penicillin/streptomycin (complete

DMEM). Before following experimental treatment, ATDC5 cells were stimulated with ITS (10μg/ml insulin, 5.5μg/ml transferrin, and 6.7ng/ml sodium selenite; Invitrogen) for 2 weeks to induce chondrogenic differentiation as previously described [9,10,11].

Mouse articular chondrocytes from global *FBXO6*$^{-/-}$ mice articular were isolated from femoral heads, femoral condyles and tibial plateaus of mice, as described previously[12]. Cultured chondrocytes were maintained as a monolayer in DMEM containing 10% fetal bovine serum (FBS) and 1% penicillin-streptomycin as previously described.

10. Nano-high performance liquid chromatography tandem mass spectrometer (Nano-HPLC-MS/MS) analysis

Chondrocytes were isolated from femoral condyles and tibial plateaus of *FBXO6*$^{f/f}$ mice, as described previously[4]. Chondrocytes were maintained as a monolayer in DMEM supplemented with 10% FBS and antibiotics, and chondrocytes were transducted 48 hours with Lentiviral-Cre (GenePharma, Shanghai, China) or negative scrambled shRNA control (LV-NC) for FBXO6 knockout.

Cells were collected (n=3 per group) and lysis buffer (2% SDS, 7M urea, 1×Protease Inhibitor Cocktail (Roche Ltd. Basel, Switzerland)) was added. The lysis was performed by sonication on ice for 3 min and kept on ice for 30 min. After centrifugation at 15000 rpm for 15 min at 4°C, the supernatant was collected and re-dissolved in 500 mM TEAB (triethylammonium bicarbonate), then transfer 100μg protein per condition into a new tube and adjust to a final volume of 100μL with 8M urea. Add 11μL of 1M DTT and incubate sample at 37°C for 1 hour, then transferred into 10K ultrafiltration tube (Millipore). To remove urea, samples were centrifuged by adding 100 mM TEAB for three times. 120μL of 55mM iodoacetamide was added to the sample and incubate for 20 minutes protected from light at room temperature. Then proteins were digested with sequence-grade modified trypsin (Promega, Madison, WI) and lyophilized. The peptides were re-dissolved in 30μL solvent A (A: 0.1% formic acid in water) and analyzed by on-line nanospray LC-MS/MS on an Orbitrap Fusion coupled to an EASY-nano-LC 1000 system (Thermo Fisher Scientific, MA, USA). 3μL peptide sample was loaded (trap column: Thermo Fisher Scientific Acclaim PepMap C18, 100μm×2cm; analytical column: Acclaim PepMap C18, 75μm×15cm) and separated with a linear gradient, from 2% B (B: 0.1% formic acid in ACN) to 25% B in 120 min. The column flow rate was maintained at 300 nL/min with the column temperature of 40°C. The electrospray voltage of 2kV versus the inlet of the mass spectrometer was used. The mass spectrometer was run under data dependent acquisition mode, and automatically switched between MS and MS/MS mode. The parameters was: (1) MS: scan range (m/z)=350–1600; resolution=60,000; AGC target=200,000; maximum injection time=50 ms; include charge states=2-6; dynamic exclusion time=24 s; (2) HCD-MS/MS: detector type=Orbitrap, resolution=15,000; isolation window=2; AGC target= 200,000; maximum injection time=70 ms; collision energy=35. Tandem mass spectra were processed by PEAKS Studio version 8.5 (Bioinformatics Solutions Inc., Waterloo, Canada). PEAKS DB was set up to search the UniProt-human database (ver.201712, 72029 entries) assuming trypsin as the digestion enzyme. PEAKS DB were searched with a fragment ion mass tolerance of 0.05 Da and a

parent ion tolerance of 7 ppm. Carbamidomethylation (C) was specified as the fixed modification. Oxidation (M), Deamidation (NQ), Acetylation (Protein N-term), were specified as the variable modifications. Peptides were filter by 1% FDR and 1 unique. ANOVA was used for peptide and protein abundance calculation. Normalization was performed on averaging the abundance of all peptides. Medians were used for averaging. Different expressed proteins were filtered if their fold change were over 1.5 and contained at least 2 unique peptides with significance over 13 ($p<0.05$).

11. Lentiviral transduction and immunoprecipitation

Lentiviral particles expressing WT mouse FBXO6 (LV-FBXO6), mouse FBXO6 knockdown shRNAs (LV−FBXO6-KD) and negative scrambled shRNA control (LV-NC) were purchased from GenePharma (Shanghai, China) with viral titer for each at 5×10^8 TU/ml. The gene sequences cloned into LV3-GFP/Puro vector were designed as follows: WT FBXO6, Forward: 5′-GCACAAGCTTTC CTGACAACA-3′, Reverse: 5′-GAATGATGCCAAATGGCAAGA-3′; and negative scrambled shRNA control: 5′-TTCTCCGAACGTGTCACGT-3′. Oligonucleotide sequences of FBXO6 shRNA-1 (KD1), Forward: 5′- GATCCGAATGATGCCAAATGGCAAGATTCAAGAGATCTTGC CATTTGGCATCATTCTTTTTTG-3′; Reverse: 5′-AATTCAAAAAAGAATGATGCCAAATGGCA AGATCTCTTGAATCTTGCCATTTGGCATCATTCG-3′; and FBXO6 shRNA-2 (KD2), Forward: 5′- GATCCGGCCTGACATTGTGGTTAAGGTTCAAGAGACCTTAACCACAATGTCAGGCCTT TTTTG-3′; Reverse: 5′-AATTCAAAAAAGGCCTGACATTGTGGTTAAGGTCTCTTGAACCTTA ACCACAATGTCAGGCCG-3′. ATDC5 were transduced with lentiviral particles at multiplicity of infection (MOI) of 800 when cells were 30-50% confluent. Culture media were changed 12hrs after viral transduction with cell viability greater than 95%. After 3 days culture all transduced cells were passaged for downstream experimentations. For the time-dependent effects of cycloheximide (CHX; Sigma-Aldrich) treatment, ATDC5 cells transduced with negative scrambled control or WT FBXO6 overexpression were treated without or with CHX (10μg/μl) for 2, 4, 6 and 8hrs and then harvested for immunoblotting analysis. For examination of protein ubiquitination, transduced ATDC5 cells were treated without or with proteasome inhibitor, MG-132 (10μg/ml, Cell Signaling Technology) for 12hrs prior to protein extraction for immunoprecipitation and immunoblotting analyses. For the analysis of N-linked glycosylation, transduced ATDC5 cells were treated without or with 500ng/ml tunicamycin-a specific inhibitor of N-linked glycosylation (#HY13585; MedChemExpress, Monmouth, NJ, USA) for 1 day prior to protein extraction for immunoblotting analysis.

For immunoblotting analysis, total cellular protein from cultured ATDC5 or global *FBXO6*$^{-/-}$ mice articular primary chondrocytes were isolated as described above using RIPA lysis buffer. Conditioned media were also collected for the analysis of secreted MMP13 protein following trichloroacetic acid (TCA)/acetone precipitation. Proteins were quantified, resolved on SDS-PAGE gel and then transferred onto PVDF membranes for immunoblot analysis as described above. Primary antibodies used includes β-actin (sc-47778; Santa Cruz), FBXO6 (11830-1-AP; Proteintech), MMP14 (ab78738; Abcam), and MMP13 (ab39012; Abcam). For immunoprecipitation assays, transduced and treated ATDC5 cells were lysed with RIPA Lysis Buffer supplemented with complete EDTA-free protease inhibitor cocktail (Sigma-Aldrich) for 30mins on ice. Lysates were cleared by

centrifugation at 13000rpm for 15mins and the supernatants were collected. Supernatants containing proteins were then incubated with 1μg of anti-MMP14 (ab78738; Abcam) antibody or 1μg of normal mouse IgG (DA1E, Cell Signaling Technology) as control overnight at 4°C. Proteins lysates were subsequently incubated with 30μl of pre-washed protein A-agarose beads (KWBIO, China) with gentle rotation for 3hrs at 4°C. The immunoprecipitates were then analyzed by immunoblotting as described above.

12. Co-localization of FBXO6 and MMP14 by fluorescence immunohistochemistry

Transduced ATDC5 cells seeded in 12-well plates at density of 2×10^5 cells/well were fixed in 4% PFA for 30mins and permeabilized with 0.2% Triton X-100 in PBS for 10mins. Cells were then incubated with FBXO6 (11830-1-AP; Proteintech) and MMP14 (ab78738; Abcam) antibodies overnight at 4°C and then incubated (1 h, room temperature) with Alex Fluor 488-conjugated goat anti-rabbit and Alex Fluor 594-conjugated goat anti-mouse secondary antibodies (1:200) at room temperature for 1hr in the dark. Three representative images from each section were captured and used to calculate the mean fluorescence intensity of MMP14 and FBXO6 in each group by ImageJ software (NIH).

13. Alcian blue staining and proteoglycan content analysis

Transduced ATDC5 cells were fixed in 4% PFA for 30min and then stained with Alcian blue (LEAGENE, Beijing, China) in accordance with manufacturer's protocol. Total amounts of glycosaminoglycans (GAGs) extracted from the ECM were measured using the 1,9-dimethylmethylene blue (DMMB; Sigma-Aldrich) colorimetric assay as described previously [13] .Papain digestion was carried out in digestion buffer (125mg/ml papain, 5mM cysteine HCl, and 5mM disodium EDTA in PBS) at 65°C for 12hrs and then diluted in DMMB dye. The absorbance reading for each sample was immediately read at wavelength of 525nm using a microplate spectrophotometer (Multiskan Sky, Thermo Fisher Scientific). Proteoglycan content in each sample were calculated based on a standard curve generated using serial dilutions of chondroitin sulfate sodium salt (Sigma-Aldrich) and the results are expressed as proteoglycan/DNA (μg/μg) values.

14. Small interfering RNA (siRNA)-mediated knockdown of MMP14 and secreted MMP13 concentration detection

Small interfering RNA (siRNA) oligonucleotides against mouse MMP14 and non-targeting scrambled control siRNA were purchased from GenePharma. ATDC5 cells plated in 6-well plate at density of 50% transfected with 100nM si-MMP14 using Lipofectamine 3000 (Thermo Fisher Scientific) in Opti-MEM (Thermo Fisher Scientific) as outlined in manufacturer's protocol. After 24hrs of transfection, the cell culture media were replaced with fresh DMEM incubated for further for 48hrs. At the end of the experimental period, cell culture conditioned media were collected and silencing of MMP14 in were assessed by immunoblot analysis as previously described. Secreted MMP13 in the conditioned media were detected following TCA/acetone precipitation as described above.

15. Effect of TGFβ-SMAD2/3 on FBXO6 expression

ATDC5 cells were cultured with increasing concentrations of human recombinant TGFβ (from 6.25ng/ml to 100ng/ml) (Sigma-Aldrich) for 24hrs followed by RNA extraction for qPCR analysis as described above. To examine the effect of Smad3 inhibitor on TGFβ induced expression of FBXO6, ATDC5 cells were treated with either 50ng/ml TGFβ or 4μM SIS-3 (Selleckchem, Houston, TX, USA) alone, or co-treated with TGFβ and SIS-3 (2 or 4μM) for 2 days followed by RNA and protein extraction for qPCR and immunoblot analyses respectively as described above.

Knee-joint cartilage explants harvested from 3-week-old Sprague-Dawley rats were initially cultured in Dulbecco's Modified Eagle's Medium (DMEM) supplemented with 10% (v/v) FBS, 100U/ml penicillin/streptomycin and 2mM L-glutamine (Invitrogen, Carlsbad, CA, USA) at 37°C with 5% CO_2. After 2 days culture, the explants were washed in serum-free media and placed in a 6-well plates with fresh serum-free media (control group), or media containing 50ng/ml human recombinant TGFβ (Sigma-Aldrich), 4μM SIS-3 (Selleckchem), or TGFβ + SIS-3 as treatment groups and cultured for 5 days. Cartilage explants were then embedded in paraffin, sectioned stained with Safranin-O/Fast Green or processed for immunofluorescence staining with anti-FBXO6 antibodies as described above.

16. Transfections and Luciferase Reporter Analysis

ATDC5 cells were transfected with FBXO6 promoter luciferase reporter (pGL3-FBXO6-promoter-luc; Vigene Bioscience, Rockville, MD, USA) containing SMAD2/3 binding motifs (AGGTGACAGAGGA) using Lipofectamine 3000 (Thermo Fisher Scientific) as per manufacturer’s instructions. FBXO6 promoter luciferase reporter without SMAD2/3 binding motifs or with mutated binding motifs (AGACAACGCGGGA) were used as controls. Luciferase activity was measured by using a dual-luciferase assay kit (Beyotime Biotech, China) according to the manufacturer’s protocol. Data were normalized for Renilla luciferase expression. results from 3 independent experiments, each with duplicate wells, were averaged.

17. Electrophoretic Mobility Shift Assay (EMSA)

An EMSA commercial kit (Beyotime Biotech, China) was used to detect the DNA binding activity of SMAD2/3 on FBXO6 promoter according to the manufacturer’s instructions. The WT FBXO6 oligonucleotide probe (5′-TCCTCTGTCACCT-3′) and mutant probe (5′-TCCCGCGTTGTCT-3′) were synthesized by TSINGKE Biological Technology and end-labeled with biotin. Nuclear extracts from mouse primary chondrocytes were incubated with the biotin-labelled probes in 20μl of EMSA reaction buffer (2μg of poly (dI-dC), 20mM HEPES [pH 7.9], 1mM $MgCl_2$, 40mM KCl, 0.1mM EDTA, 1mM DTT and 12% glycerol) and binding reactions proceeded for 20mins. In competition experiments, excess of unlabeled oligonucleotide probe was added to the EMSA reaction mixture 10mins before the labelled probe. In supershift experiments, SMAD2/3 antibodies (1μg) were added after 20mins binding reaction and incubation continued for a further 30mins. Protein-DNA complexes were resolved on 5% polyacrylamide gels containing 0.5 × TBE gels running buffer at 150V for 1.5hrs at room temperature and then detected with phosphoimager (Bio-Rad Laboratories,

Hercules, CA, USA).

18. Chromatin immunoprecipitation (ChIP) assays

Cellular lysates were extracted from cultured ATDC5 cells and ChIP assays were performed using the SimpleChIP Enzymatic Chromatin Agarose Beads IP Kit (#9002; Cell Signaling Technology) in accordance with manufacturer's protocol. In brief, ATDC5 cells treated without or without SIS-3 for 24hrs then stimulated with TGFβ were fixed with 1% formaldehyde for 10 min at 37°C to crosslink proteins to DNA. Cells were then washed with ice-cold PBS and lysed with ice-cold PBS containing protease inhibitor cocktail (Sigma-Aldrich). The following lysis samples were sonicated to shear DNA and cell debris removed by centrifugation. Chromatin was then fragmented by partial digestion with Micrococcal Nuclease to obtain chromatin fragments of 1 to 5 nucleosomes. Fragmented chromatins were then subjected to overnight immunoprecipitation at 4°C with specific antibodies against SMAD2/3 (#8685, Cell Signaling Technology), Smad4 (#46535, Cell Signaling Technology) or control IgG (#2729, Cell Signaling Technology). Immuoprecipitates were then incubated with ChIP-Grade Protein G Agarose Beads for 2hrs at 4°C with rotation. After several low and high salt washes, reversal of protein-DNA cross-links were carried out and the resulting DNAs were purified using DNA purification spin columns. Purified DNAs were subjected to PCR amplification and agarose gel electrophoresis or quantitative real-time PCR as described above using the following primers sets detailed in Table 2 below:

Table 2. Predicted SMAD2/3/4 binding sites on FBXO6 promoter

**Primers for:**	**Forward (5′ → 3′)**	**Reverse (5′ → 3′)**
Predicted binding site 1	CACCTTCAGCACACGGGTT	AACGTAATTCTCTGTTGCCACC
Predicted binding site 2	GATGTGGACCATGGTGGCAACAG	GCGATCCAGAACCTGATGGGA
Predicted binding site 3	TCAGCTCTCCAGGGCCTT	AGAGCTCCGAGCTCACCATC

19. Statistical analysis

Data are presented as means ± SEM. The ‘n’ values for each figure correspond to the number of independent experiments or mice used. Data quantified based on an ordinal grading system, such as OARSI grade and analyzed using non-parametric statistical methods. Real-time qPCR data are expressed as relative fold changes. Statistical significance was calculated using the two-tailed unpaired Student’s *t*-test or by ANOVA for multiple comparisons. A difference between experimental groups was considered significant when the p-value was less than 0.05. SPSS Statistics software version 19.0 (IBM, Armonk, NY, USA) was used for the statistical analyses.

Result:

TGFβ-SMAD2/3 signaling pathway induced FBXO6 gene transcription

Finally, we showed direct binding of SMAD2/3 to the FBXO6 promoter by electrophoretic mobility shift assays (EMSA). Nuclear extracts from control, TGFβ and TGFβ + SIS-3 treated cells were incubated with biotin-labeled DNA probe containing the putative SMAD2/3 binding element alone, in combination with excess unlabeled DNA probe (cold competitor), or with mutant DNA probe

(Supplementary Figure S5A) and the ran on a gel. Retarded complexes could be seen following TGFβ treatment which was abolished following treatment with SMAD3 inhibitor SIS-3 or in the presence of excess unlabeled DNA probes. No retarded bands were observed with the mutant DNA probe. Incubation with SMAD2/3 antibody resulted in a super-shifted complex further confirming the binding of SMAD2/3 to the WT FBXO6 DNA probe (Supplementary Figure S5B).

FBXO6 regulated MMP14 ubiquitination and degradation

Cell viability and transduction efficiency was assessed. As shown in Supplementary Figure S6A, cells transduced with lentiviral particles did not show any adverse or cytotoxic effects with proliferation rates comparted with untransduced cells as assessed by CCK-8 cell proliferation assay. Transduction efficiency was assessed by qPCR and the two FBXO6 shRNA lentiviral constructs (KD1 and KD2) showed more than 50% reduction in FBXO6 expression whereas the FBXO6 overexpression lentiviral showed doubled the expression of FBXO6 (Supplementary Figure S6B). The gene expression levels of other proteinase such as MMP3, MMP13, ADMATS4, and ADAMTS5; ECM proteins such as Col-2a1 and aggrecan; and chondrogenic transcription factor Sox9 were not affected by FBXO6 knockdown or overexpression (Supplementary Figure S6C), excluding that FBXO6 could not modulate anabolic and catabolic gene expression directly.

References

1. Kumar R, Grønhaug KM, Afseth NK, et al. Optical investigation of osteoarthritic human cartilage (ICRS grade) by confocal Raman spectroscopy: a pilot study. Anal Bioanal Chem. 2015. 407(26): 8067-77.
2. Jeon OH, Kim C, Laberge RM, et al. Local clearance of senescent cells attenuates the development of post-traumatic osteoarthritis and creates a pro-regenerative environment. Nat Med. 2017. 23(6): 775-781.
3. Glasson SS, Chambers MG, Van Den Berg WB, Little CB. The OARSI histopathology initiative - recommendations for histological assessments of osteoarthritis in the mouse. Osteoarthritis Cartilage. 2010. 18 Suppl 3: S17-23.
4. Rhee J, Park SH, Kim SK, et al. Inhibition of BATF/JUN transcriptional activity protects against osteoarthritic cartilage destruction. Ann Rheum Dis. 2017. 76(2): 427-434.
5. Zhen G, Wen C, Jia X*, et al.* Inhibition of TGF-β signaling in mesenchymal stem cells of subchondral bone attenuates osteoarthritis. *Nat Med* 2013;**19**:704-12.
6. Kim JH, Jeon J, Shin M*, et al.* Regulation of the catabolic cascade in osteoarthritis by the zinc-ZIP8-MTF1 axis. *Cell* 2014;**156**:730-43.
7. Jia H, Ma X, Wei Y*, et al.* Loading-Induced Reduction in Sclerostin as a Mechanism of Subchondral Bone Plate Sclerosis in Mouse Knee Joints During Late-Stage Osteoarthritis. *Arthritis Rheumatol* 2018;**70**:230-41.
8. Li J, Xue J, Jing Y*, et al.* SOST Deficiency Aggravates Osteoarthritis in Mice by Promoting Sclerosis of Subchondral Bone. *Biomed Res Int* 2019;**2019**:7623562.
9. Miyatake K, Tsuji K, Yamaga M, et al. Human YKL39 (chitinase 3-like protein 2), an osteoarthritis-associated gene, enhances proliferation and type II collagen expression in ATDC5 cells[J]. Biochem Biophys Res

Commun, 2013,431(1):52-57. DOI: 10.1016/j.bbrc.2012.12.094.

10. Otero M, Gomez Reino JJ, Gualillo O. Synergistic induction of nitric oxide synthase type II: in vitro effect of leptin and interferon-gamma in human chondrocytes and ATDC5 chondrogenic cells. Arthritis Rheum. 2003. 48(2): 404-9.
11. Yoshimura K, Miyamoto Y, Yasuhara R, et al. Monocarboxylate transporter-1 is required for cell death in mouse chondrocytic ATDC5 cells exposed to interleukin-1beta via late phase activation of nuclear factor kappaB and expression of phagocyte-type NADPH oxidase. J Biol Chem. 2011. 286(17): 14744-52.
12. Dai J, Yu D, Wang Y*, et al.* Kdm6b regulates cartilage development and homeostasis through anabolic metabolism. *Ann Rheum Dis* 2017.
13. Chen K, Lin X, Zhang Q, et al. Decellularized periosteum as a potential biologic scaffold for bone tissue engineering. Acta Biomater. 2015. 19: 46-55.
