# Supplementary material for: TGFβ attenuates cartilage extracellular matrix degradation via enhancing FBXO6-mediated MMP14 ubiquitination
Source: Ann Rheum Dis. 2020 May 14;79(8):1111–20. doi: 10.1136/annrheumdis-2019-216911 (PMC7392491; doi:10.1136/annrheumdis-2019-216911)
Supplement: Supplementary data [file annrheumdis-2019-216911supp002.pdf]

Supplementary Figure S1
FBXO6 expression level was parallelly decreased with TGFβ-SMAD2/3 pathway in human OA cartilage.
(A) H&E staining for human moderate and severe degenerated cartilage. (B) Gene expression levels of F-box proteins 2, 6, 17, 27 and 44 in moderate and severe degenerated cartilage from patients from total hip or knee replacement surgery. (C) Western blot analysis of the protein expression levels of Col2 and FBXO6 in moderate and severe degenerated cartilage.
(Data are expressed as mean ± SD. * $p<0.05$.).

Supplementary Figure S2
FBXO6 showed little effects on subchondral bone sclerosis directly
(A) Comparison of B.Ar/T.Ar and SBP between WT and global *FBXO6*$^{-/-}$ mice in sham operation and ACLT-induced OA group. (B) The body weight and length (body and tail) between *global FBXO6*$^{-/-}$ and WT mice at 4 or 20 weeks were compared. (C) Skeletal, forelimbs (humerus and ulna) and hindlimbs (femur and tibia) staining of newborn WT and *global FBXO6*$^{-/-}$ mouse pups with Alcian blue and Alizarin red. (D) Bone lengths of forelimbs and hindlimbs from newborn WT and *global FBXO6*$^{-/-}$ mouse pups measured.
(Data are expressed as mean ± SD. * $p<0.05$).

Supplementary Figure S3
Micro-CT of knee joints in *Col2a1-CreER*^{T2}*; FBXO6*$^{f/f}$ and wide type mice
(A) Micro-CT represented excessive osteophyte formation of mouse injured joints of *Col2a1-CreER*^{T2}*; FBXO6*$^{f/f}$ and wide type mice at 8 weeks post-injury. (B) Reconstructed 2D coronal and sagittal images of knee joints in *Col2a1-CreER*^{T2}*; FBXO6*$^{f/f}$ and wide type mice. (C) Comparison of SBP and BV/TV between *Col2a1-CreER*^{T2}*; FBXO6*$^{f/f}$ and wide type mice.
(Data are expressed as mean ± SD. * $p<0.05$; ** $p<0.01$.).

Supplementary Figure S4
FBXO6 regulated MMP14 ubiquitination and degradation
(A) Western blotting detected the change of active-MMP13 in cell medium after MMP14 was silenced. (B) MMP14 was upregulated in FBXO6$^{-/-}$ chondrocytes. (C) Co-IP to confirm the bind between FBXO6 and MMP14 in SW-1353 cell line. (D) Immunofluorescence for detection of co-localization of FBXO6 and MMP14 (1000×). (E) Western blotting detected FBXO6-induced MMP14 degradation level after the administration of MG132.

Supplementary Figure S5
TGFβ-SMAD2/3 signaling pathway induced FBXO6 gene transcription.
(A) Schematic diagram of the mutant probes for EMSA. (B) EMSA using nuclear extracts from ATDC5 cells is shown.

Supplementary Figure S6
ATCD5 cells infected with different FBXO6 lentivirus.
(A) CCK-8 assay to evaluate cell viability following ATCD5 cells infected with scramble vehicle lentivirus, 2 FBXO6 knockdown lentivirus and FBXO6 overexpression lentivirus and cultured for 1-8 days. (B) lentivirus transfection efficiency of 5 different lentivirus was assessed by qRT-PCR. (C) expression of MMP3, MMP13, ADMATS4, ADAMTS5, Col-2a1, Aggrecan and Sox9 were assessed by qRT-PCR in 4 different lentivirus transfected cells
(Data are expressed as mean ± SD. *** $p<0.001$.).

Supplementary Figure S7
GFP fluorescence after intra-articular injection of LV-GFP

(A) Immunofluorescence for GFP fluorescence in joint sections of mice after intra-articular injection of LV-GFP. (B) Comparison of B.Ar/T.Ar and SBP between LV-Con and LV-FBXO6 mice in sham operation and ACLT-induced OA group.
(Scale bars, 100 μm. Data are expressed as mean ± SD. * $p<0.05$).
