# Supplementary figures and images for: TGFβ attenuates cartilage extracellular matrix degradation via enhancing FBXO6-mediated MMP14 ubiquitination
Source: Ann Rheum Dis. 2020 May 14;79(8):1111–20. doi: 10.1136/annrheumdis-2019-216911 (PMC7392491; doi:10.1136/annrheumdis-2019-216911)

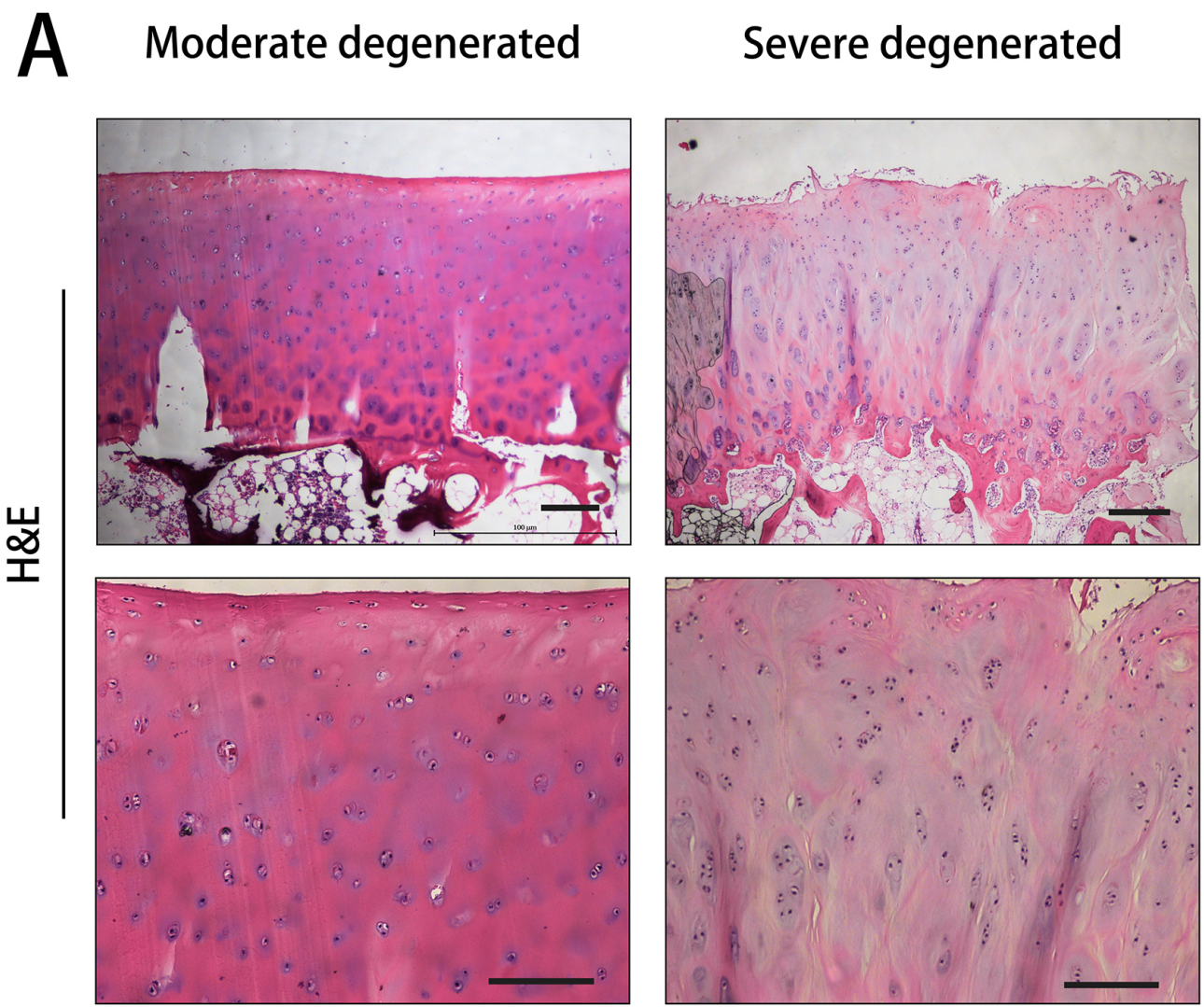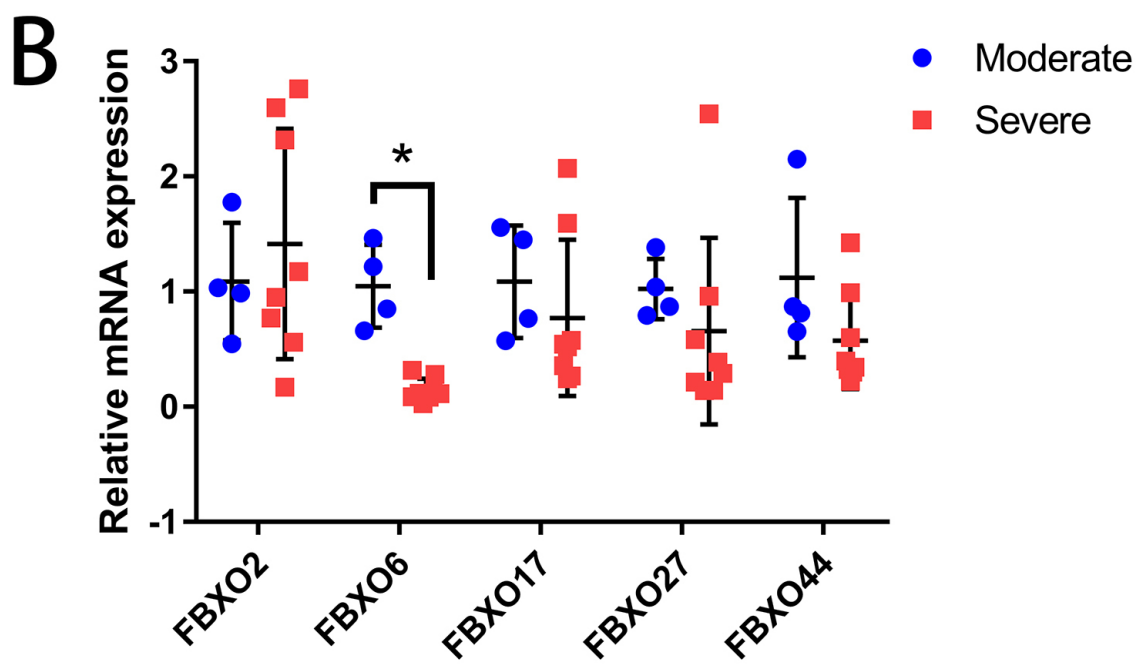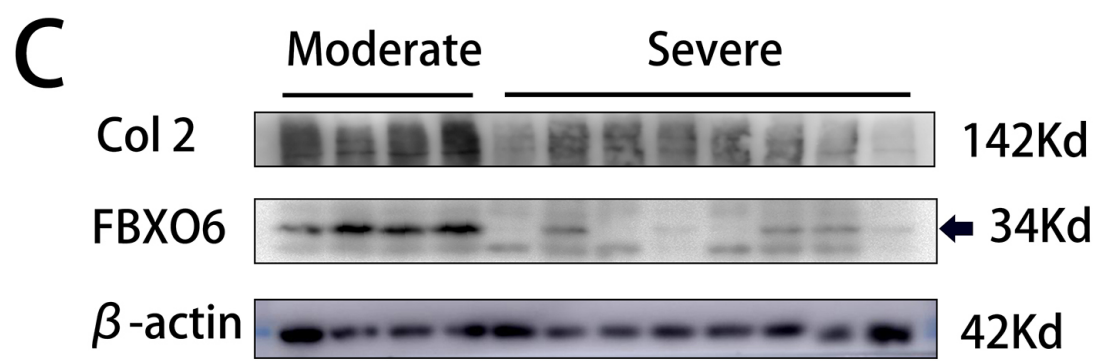

Supplement: Supplementary data [file annrheumdis-2019-216911supp003.pdf]

A

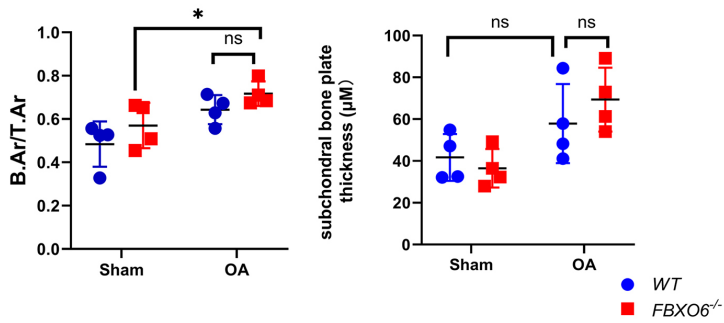

B

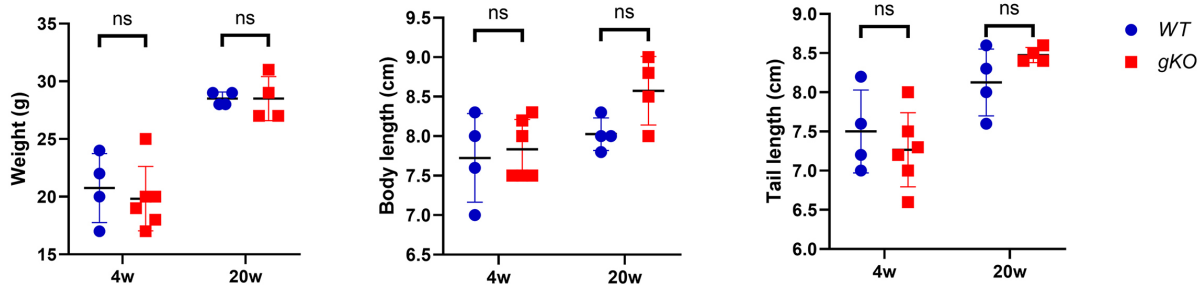

C

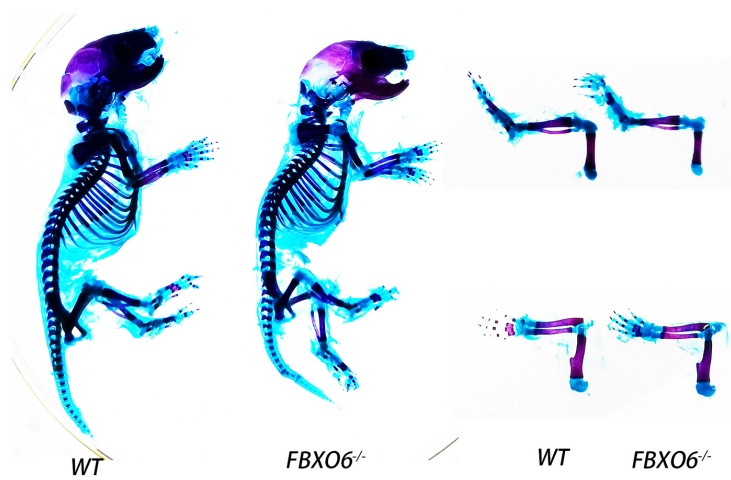

D

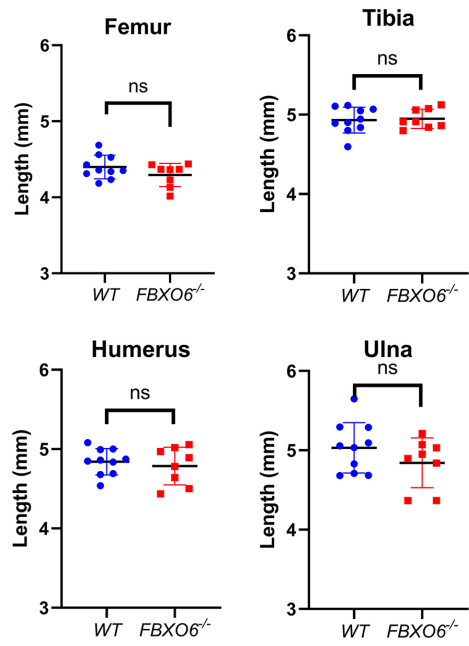

Supplement: Supplementary data [file annrheumdis-2019-216911supp004.pdf]

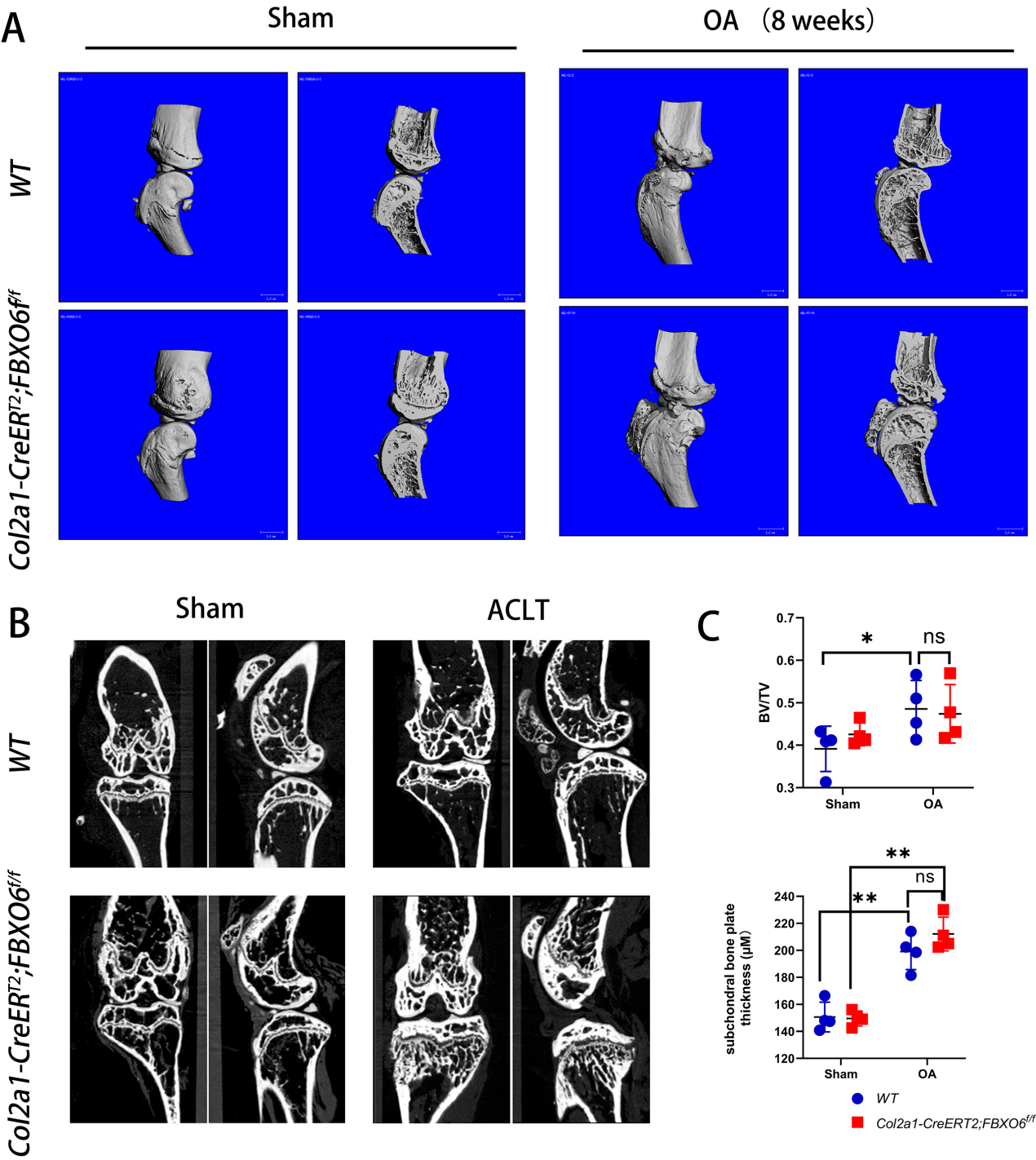

Supplement: Supplementary data [file annrheumdis-2019-216911supp005.pdf]

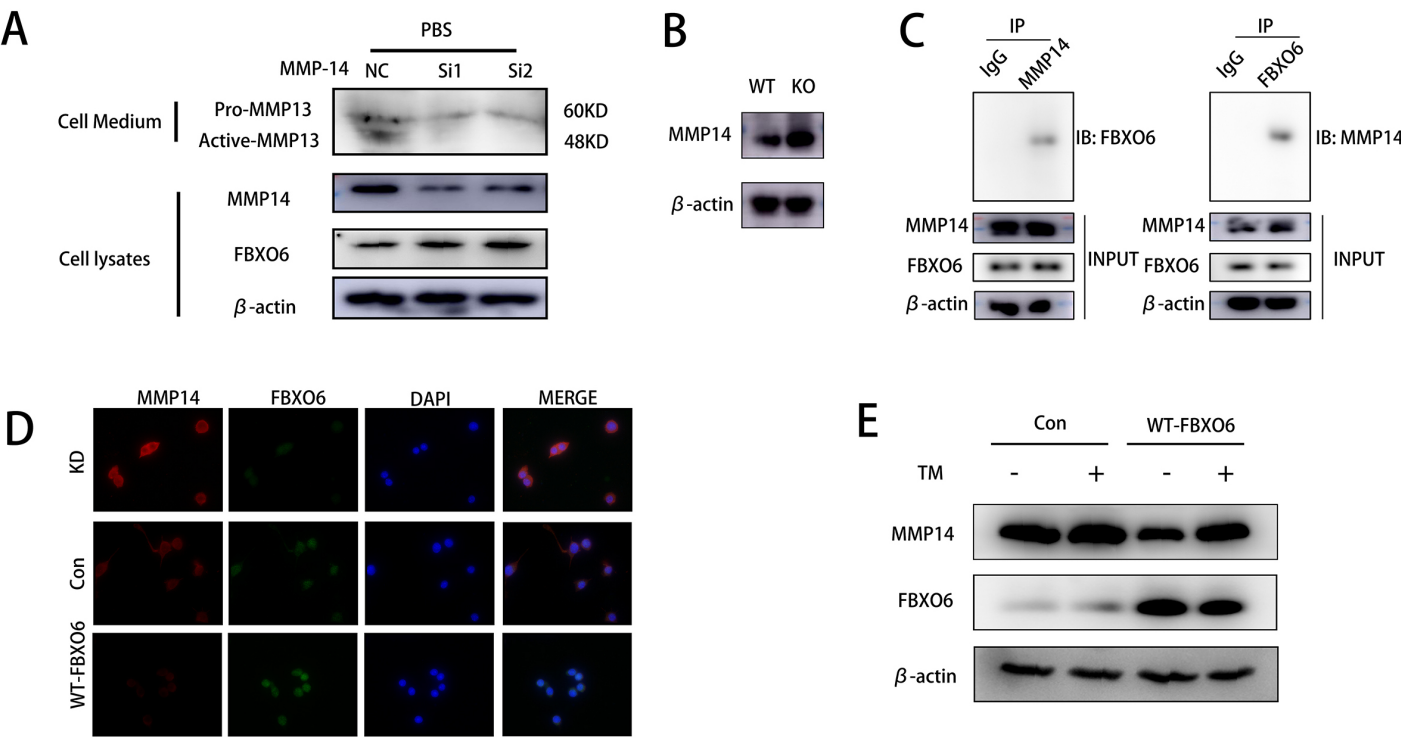

Supplement: Supplementary data [file annrheumdis-2019-216911supp009.pdf]

A

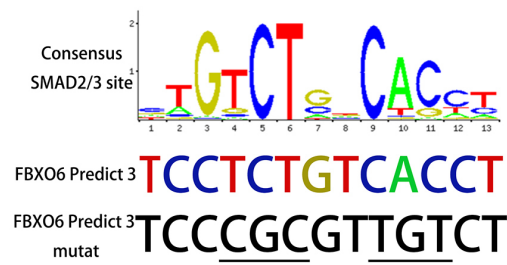

B

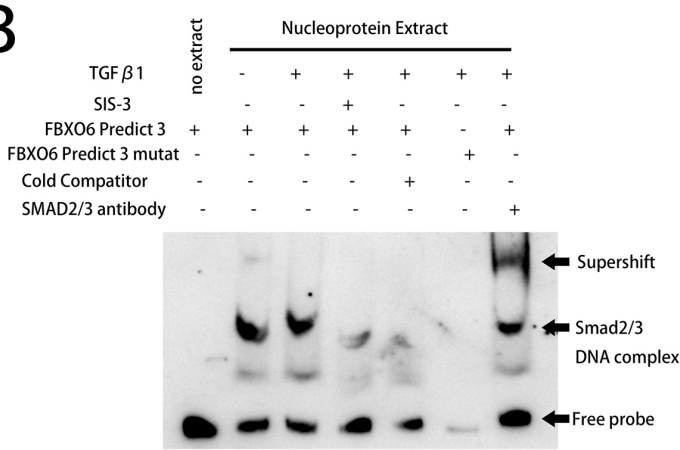

Supplement: Supplementary data [file annrheumdis-2019-216911supp010.pdf]

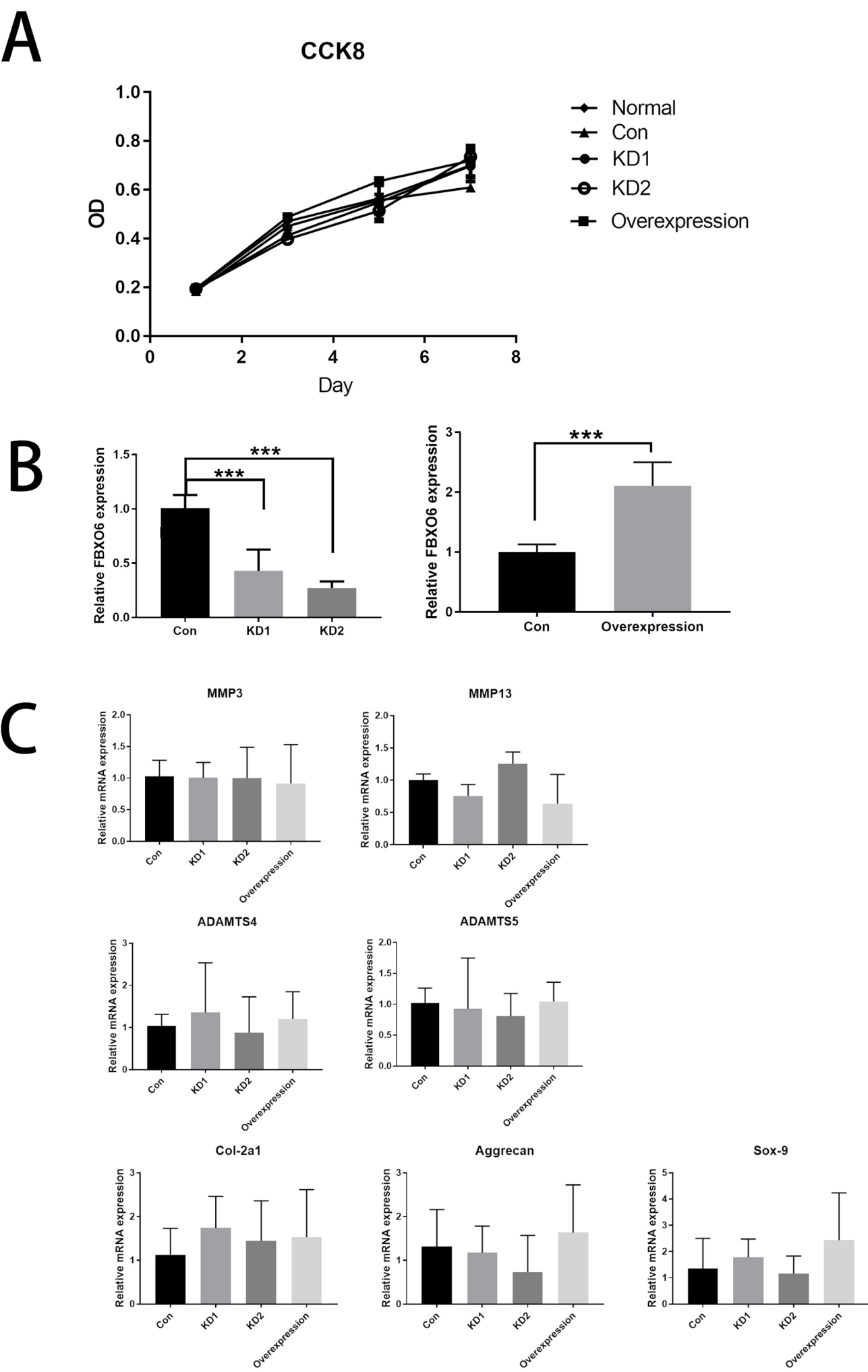

Supplement: Supplementary data [file annrheumdis-2019-216911supp011.pdf]

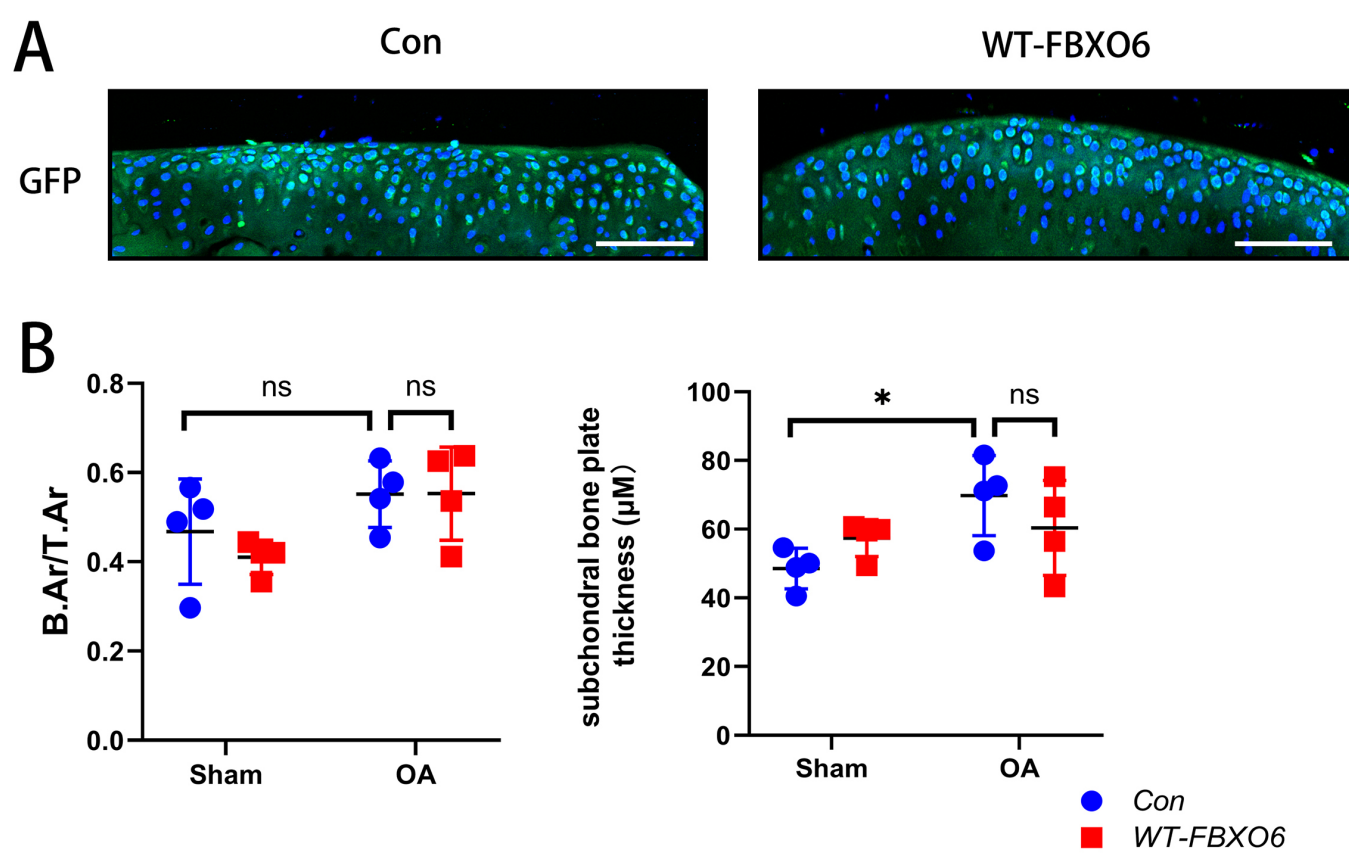

Supplement: Supplementary data [file annrheumdis-2019-216911supp012.pdf]
