# Supplementary material for: TGFβ attenuates cartilage extracellular matrix degradation via enhancing FBXO6-mediated MMP14 ubiquitination
Source: Ann Rheum Dis. 2020 May 14;79(8):1111–20. doi: 10.1136/annrheumdis-2019-216911 (PMC7392491; doi:10.1136/annrheumdis-2019-216911)
Supplement: Supplementary data [file annrheumdis-2019-216911supp007.pdf]

ID	Gene Name
gene name	
main functions	
assosiation with oc\ob\cartilage\disc(np af ep notochord cells)	
IPI00420067	ADAM metallopeptidase domain 15
IPI00288894	ADAM metallopeptidase domain 17
IPI00307592	ATP-binding cassette, sub-family A (ABC1), member 2
IPI00291373	ATP-binding cassette, sub-family D (ALD), member 1
IPI00002372	ATP-binding cassette, sub-family D (ALD), member 3
IPI00784119	ATPase, H+ transporting, lysosomal accessory protein 1
IPI00021391	C-type lectin domain family 2, member B
IPI00788676	CD109 molecule
IPI00384548	CD63 molecule
IPI00736241	CUB domain containing protein 1
IPI00844115	DnaJ (Hsp40) homolog, subfamily C, member 10
IPI00005683	ER degradation enhancer, mannosidase alpha-like 1
IPI00892978	ER degradation enhancer, mannosidase alpha-like 3
IPI00007940	ER lipid raft associated 1
IPI00026942	ER lipid raft associated 2
IPI00383663	ERGIC and golgi 3
IPI00872773	ERO1-like (S. cerevisiae)
IPI00334818	FK506 binding protein 10, 65 kDa
IPI00943593	Fraser syndrome 1
IPI00879033	G protein-coupled receptor 110
IPI00445699	KDEL (Lys-Asp-Glu-Leu) containing 2
IPI00645487	KIAA0090
IPI00885086	KIAA0746 protein
IPI00456649	KIAA1161
IPI00008787	N-acetylglucosaminidase, alpha-
IPI00940046	N-acylsphingosine amidohydrolase (acid ceramidase) 1
IPI00005600	N-deacetylase/N-sulfotransferase (heparan glucosaminyl) 2
IPI00019988	N-sulfoglucosamine sulfohydrolase
IPI00794861	NEL-like 2 (chicken)

IPI00413732	NODAL modulator 3; NODAL modulator 1; NODAL modulator 2
IPI00646796, IPI00005107	Niemann-Pick disease, type C1
IPI00297492	STT3, subunit of the oligosaccharyltransferase complex, homolog A
IPI00152377	STT3, subunit of the oligosaccharyltransferase complex, homolog B
IPI00642739	TIMP metallopeptidase inhibitor 1
IPI00294455	UDP glycosyltransferase 8
IPI00031983	UDP-GlcNAc:betaGal beta-1,3-N-acetylglucosaminyltransferase 3
IPI00026991	UDP-N-acetyl-alpha-D-galactosamine:polypeptide N-acetylgalactosaminyltransferase 6
IPI00002511	activating transcription factor 6
IPI00374563	agrin
IPI00745872	albumin
IPI00296524	arylsulfatase family, member K
IPI00021552	beta-1,3-N-acetylgalactosaminyltransferase 1 (globoside blood group)
IPI00744286	beta-1,3-N-acetylgalactosaminyltransferase 2
IPI00025073	bone morphogenetic protein 3。。。
IPI00946206	butyrylcholinesterase
IPI00658202	cadherin 2, type 1, N-cadherin (neuronal)
IPI00008403	carbohydrate (N-acetylglucosamine 6-O) sulfotransferase 7
IPI00748502	cartilage associated protein
IPI00640525	cathepsin A
IPI00022810	cathepsin C
IPI00011229	cathepsin D
IPI00642813	cerebral endothelial cell adhesion molecule
IPI00465319	chondroitin polymerizing factor
IPI00945406	chondroitin sulfate glucuronyltransferase
IPI00432337	chromosome 2 open reading frame 30
IPI00171385	chromosome 3 open reading frame 39
IPI00400826	clusterin
IPI00291136	collagen, type VI, alpha 1
IPI00001245	contactin associated protein-like 3; contactin associated protein-like 3B
IPI00794466	crystallin, alpha B
IPI00386115	cyclin M3
IPI00218928	desmocollin 3

IPI00025753	desmoglein 1
IPI00216370	ectonucleoside triphosphate diphosphohydrolase 2
IPI00184311	ectonucleotide pyrophosphatase/phosphodiesterase 1
IPI00165949	endoplasmic reticulum aminopeptidase 1
IPI00747807	endoplasmic reticulum aminopeptidase 2
IPI00216758	endothelin converting enzyme 1
IPI00657648	ependymin related protein 1 (zebrafish)
IPI00012440	fucosidase, alpha-L- 2, plasma
IPI00025869	galactosidase, alpha
IPI00796919	galactosidase, beta 1
IPI00012102	glucosamine (N-acetyl)-6-sulfatase
IPI00883772	glucosidase, alpha; acid
IPI00383581	glucosidase, alpha; neutral AB
IPI00759616	glucosidase, beta; acid (includes glucosylceramidase)
IPI00219516	glucuronidase, beta
IPI00100656	glycoprotein, synaptic 2
IPI00168262	glycosyltransferase 25 domain containing 1
IPI00296713	granulin
IPI00027230	heat shock protein 90kDa beta (Grp94), member 1
IPI00943326	heparan sulfate proteoglycan 2
IPI00607861	hexose-6-phosphate dehydrogenase (glucose 1-dehydrogenase)
IPI00023601	hyaluronan and proteoglycan link protein 1
IPI00000877	hypoxia up-regulated 1
IPI00902482	iduronidase, alpha-L-
IPI00903213	insulin-like growth factor 2 receptor
IPI00006547	integrin alpha FG-GAP repeat containing 1
IPI00845516	integrin alpha FG-GAP repeat containing 3
IPI00290043	integrin, alpha 3 (antigen CD49C, alpha 3 subunit of VLA-3 receptor)
IPI00306604	integrin, alpha 5 (fibronectin receptor, alpha polypeptide)
IPI00216225	integrin, alpha 6
IPI00555991	integrin, alpha V (vitronectin receptor, alpha polypeptide, antigen CD51)
IPI00217563	fibronectin receptor, beta polypeptide, antigen CD29 includes MDF2, MSK12
IPI00029741	integrin, beta 5
IPI00642425	intercellular adhesion molecule 1
IPI00554788	keratin 18; keratin 18 pseudogene 26; keratin 18 pseudogene 19

IPI00554648	keratin 8 pseudogene 9; similar to keratin 8; keratin 8
IPI00743994	laminin, alpha 3
IPI00783665	laminin, alpha 5
IPI00853454	laminin, beta 1
IPI00299404	laminin, beta 3
IPI00298281	laminin, gamma 1 (formerly LAMB2)
IPI00023673	lectin, galactoside-binding, soluble, 3 binding protein
IPI00293303	legumain
IPI00045839	leucine proline-enriched proteoglycan (leprecan) 1
IPI00478892	leucine-rich repeats and immunoglobulin-like domains 2
IPI00221241	leucyl/cystinyl aminopeptidase
IPI00908663	low density lipoprotein receptor
IPI00414231	low density lipoprotein receptor-related protein 10
IPI00020557	low density lipoprotein-related protein 1
IPI00908532	lysosomal-associated membrane protein 1
IPI00216172	lysosomal-associated membrane protein 2
IPI00294839	lysyl oxidase-like 2
IPI00219706	macrophage stimulating 1 receptor (c-met-related tyrosine kinase)
IPI00644131	mannosidase, alpha, class 2B, member 1
IPI00298793	mannosidase, beta A, lysosomal
IPI00896498	matrix metallopeptidase 14 (membrane-inserted)
IPI00294528	met proto-oncogene (hepatocyte growth factor receptor)
IPI00918002	mucin 5B, oligomeric mucus/gel-forming
IPI00018311	neuroplastin
IPI00470649	nicalin homolog (zebrafish)
IPI00375688	nicastrin
IPI00291755	nucleoporin 210kDa
IPI00737429	odz, odd Oz/ten-m homolog 4 (Drosophila)
IPI00329760	osteosarcoma amplified 9, endoplasmic reticulum associated protein
IPI00290945	paraoxonase 2
IPI00016112	peroxidasin homolog (Drosophila)
IPI00465308	phosphatidylinositol glycan anchor biosynthesis, class S
IPI00401043	phosphatidylinositol glycan anchor biosynthesis, class T
IPI00301459	phospholipase A2, group XV
IPI00328243	phospholipase D family, member 3

IPI00479511	plasminogen activator, tissue
IPI00219426	poliovirus receptor
IPI00384280	prenylcysteine oxidase 1
IPI00184180	prenylcysteine oxidase 1 like
IPI00943008	procollagen-lysine 1, 2-oxoglutarate 5-dioxygenase 1
IPI00472165	procollagen-lysine, 2-oxoglutarate 5-dioxygenase 2
IPI00030255	procollagen-lysine, 2-oxoglutarate 5-dioxygenase 3
IPI00009923	prolyl 4-hydroxylase, alpha polypeptide I
IPI00399307	prolylcarboxypeptidase (angiotensinase C)
IPI00012503	prosaposin
IPI00218176	protein O-fucosyltransferase 1
IPI00329648	protein-O-mannosyltransferase 2
IPI00465016	quiescin Q6 sulfhydryl oxidase 1
IPI00793589, IPI00025874	ribophorin I
IPI00383680	ribophorin II
IPI00395914	scavenger receptor class A, member 5 (putative)
IPI00291007	scavenger receptor class B, member 1
IPI00002790	sel-1 suppressor of lin-12-like (C. elegans)
IPI00644196	serpin peptidase inhibitor, clade B (ovalbumin), member 5
IPI00914848	serpin peptidase inhibitor, clade E , member 2
IPI00759728	sialic acid acetylesterase
IPI00019472	solute carrier family 1 (neutral amino acid transporter), member 5
IPI00554481	solute carrier family 3, member 2
IPI00022608	sortilin-related receptor, L(DLR class) A repeats-containing
IPI00005614, IPI00328230	spectrin, beta, non-erythrocytic 1
IPI00297252	sulfatase 2
IPI00739876	tectonic family member 3
IPI00220213	tenascin C
IPI00064193	thioredoxin-related transmembrane protein 3
IPI00007798	thyrotropin-releasing hormone degrading enzyme
IPI00168878	torsin A interacting protein 2
IPI00941838	torsin family 1, member B (torsin B)

IPI00022462, IPI00924935	transferrin receptor (p90, CD71)
IPI00000075	transforming growth factor, beta 1
IPI00220156	transforming growth factor, beta 2
IPI00470924	transmembrane and tetratricopeptide repeat containing 3
IPI00028055	transmembrane emp24-like trafficking protein 10 (yeast)
IPI00031064	transmembrane protein 126A
IPI00916157	transmembrane protein 131
IPI00013683	tubulin, beta 3; melanocortin 1 receptor
