# Supplementary material for: TGFβ attenuates cartilage extracellular matrix degradation via enhancing FBXO6-mediated MMP14 ubiquitination
Source: Ann Rheum Dis. 2020 May 14;79(8):1111–20. doi: 10.1136/annrheumdis-2019-216911 (PMC7392491; doi:10.1136/annrheumdis-2019-216911)
Supplement: Supplementary data [file annrheumdis-2019-216911supp008.pdf]

Gene.Nam	Gene ontology (biological process)	Gene ontology (cellular component)	Gene ontology (molecular function)
Lgals3bp	cell adhesion [GO:0007155]	extracellular matrix [GO:0031012]; extracellular region [GO:0005576]; extracellular space [GO:0005615]; membrane [GO:0016020]; proteinaceous extracellular matrix [GO:0005578]	scavenger receptor activity [GO:0005044]
Pxdn	extracellular matrix organization [GO:0030198]; hydrogen peroxide catabolic process [GO:0042744]; oxidation-reduction process [GO:0055114]; response to oxidative stress [GO:0006979]	endoplasmic reticulum [GO:0005783]; extracellular matrix [GO:0031012]; extracellular space [GO:0005615]; proteinaceous extracellular matrix [GO:0005578]	extracellular matrix structural constituent [GO:0005201]; heme binding [GO:0020037]; metal ion binding [GO:0046872]; peroxidase activity
Chst14	carbohydrate biosynthetic process [GO:0016051]; dermatan sulfate proteoglycan metabolic process [GO:0050655]	Golgi membrane [GO:0000139]; integral component of membrane [GO:0016021]	N-acetylgalactosamine 4-O-sulfotransferase activity [GO:0001537]
Pvr	cell-cell adhesion [GO:0098609]; cell migration [GO:0016477]; cell recognition [GO:0008037]; heterophilic cell-cell adhesion via plasma membrane cell adhesion molecules [GO:0007157]; homophilic cell adhesion via plasma membrane adhesion molecules [GO:0007156]; positive regulation of natural killer cell mediated cytotoxicity directed against tumor cell target [GO:0002860]; susceptibility to natural killer cell mediated cytotoxicity [GO:0042271]; susceptibility to T cell mediated cytotoxicity [GO:0060370]	cell-cell adherens junction [GO:0005913]; cell surface [GO:0009986]; integral component of plasma membrane [GO:0005887]; plasma membrane [GO:0005886]	cell adhesion molecule binding [GO:0050839]; dynein light chain binding [GO:0045503]; protein homodimerization activity [GO:0042803]; receptor activity [GO:0004872]; receptor binding [GO:0005102]
Enpp1	immune response [GO:0006955]; metabolic process [GO:0008152]	integral component of membrane [GO:0016021]	hydrolase activity [GO:0016787]; metal ion binding [GO:0046872]; nucleic acid binding [GO:0003676]; polysaccharide binding [GO:0030247]; scavenger receptor activity

Mmp14	[illegible] [GO:0043615]; bone development [GO:0060348]; branching morphogenesis of an epithelial tube [GO:0048754]; cell migration [GO:0016477]; chondrocyte proliferation [GO:0035988]; collagen catabolic process [GO:0030574]; craniofacial suture morphogenesis [GO:0097094]; embryonic cranial skeleton morphogenesis [GO:0048701]; endochondral ossification [GO:0001958]; endodermal cell differentiation [GO:0035987]; endothelial cell proliferation [GO:0001935]; head development [GO:0060322]; lung development [GO:0030324]; male gonad development [GO:0008584]; negative regulation of focal adhesion assembly [GO:0051895]; negative regulation of Notch signaling pathway [GO:0045746]; ossification [GO:0001503]; ovarian follicle development [GO:0001541]; positive regulation of B cell differentiation [GO:0045579]; positive regulation of cell growth [GO:0030307]; positive regulation of cell migration [GO:0030335]; positive regulation of macrophage migration [GO:1905523]; positive regulation of myotube differentiation [GO:0010831]; proteolysis [GO:0006508]; response to estrogen [GO:0043627]; response to hormone [GO:0009725]; response to hypoxia [GO:0001666]; response to mechanical stimulus [GO:0009612]; response to odorant [GO:1990834]; response to organic cyclic	cytoplasmic vesicle [GO:0031410]; cytosol [GO:0005829]; extracellular matrix [GO:0031012]; extracellular space [GO:0005615]; integral component of membrane [GO:0016021]; intermediate filament cytoskeleton [GO:0045111]; macropinosome [GO:0044354]; melanosome [GO:0042470]; nucleus [GO:0005634]; plasma membrane [GO:0005886]	integrin binding [GO:0005178]; metalloaminopeptidase activity [GO:0070006]; metalloendopeptidase activity [GO:0004222]; peptidase activator activity [GO:0016504]; zinc ion binding [GO:0008270]
Gprc5a		integral component of membrane [GO:0016021]; nucleolus [GO:0005730]; plasma membrane [GO:0005886]; vesicle [GO:0031982]	cadherin binding [GO:0045296]; G-protein coupled receptor activity [GO:0004930]
Pcyox1	prenylated protein catabolic process [GO:0030327]; prenylcysteine catabolic process [GO:0030328]	extracellular region [GO:0005576]; vacuolar membrane [GO:0005774]	prenylcysteine oxidase activity [GO:0001735]

Ece1	bradykinin catabolic process [GO:0010815]; calcitonin catabolic process [GO:0010816]; ear development [GO:0043583]; embryonic digit morphogenesis [GO:0042733]; endothelin maturation [GO:0034959]; heart development [GO:0007507]; hormone catabolic process [GO:0042447]; peptide hormone processing [GO:0016486]; pharyngeal system development [GO:0060037]; positive regulation of receptor recycling [GO:0001921]; protein processing [GO:0016485]; substance P catabolic process	cell surface [GO:0009986]; early endosome [GO:0005769]; endosome [GO:0005768]; external side of plasma membrane [GO:0009897]; Golgi apparatus [GO:0005794]; integral component of membrane [GO:0016021]; perinuclear region of cytoplasm [GO:0048471]; plasma membrane [GO:0005886]; Weibel-Palade body [GO:0033093]	endopeptidase activity [GO:0004175]; metalloendopeptidase activity [GO:0004222]; protein homodimerization activity [GO:0042803]; zinc ion binding [GO:0008270]

Hdac2	[illegible] cardiac muscle hypertrophy [GO:0003300]; cellular response to dopamine [GO:1903351]; cellular response to heat [GO:0034605]; cellular response to hydrogen peroxide [GO:0070301]; cellular response to retinoic acid [GO:0071300]; cellular response to transforming growth factor beta stimulus [GO:0071560]; maintenance of chromatin silencing [GO:0006344]; negative regulation of dendritic spine development [GO:0061000]; negative regulation of DNA binding [GO:0043392]; negative regulation of DNA binding transcription factor activity [GO:0043433]; negative regulation of neuron projection development [GO:0010977]; negative regulation of peptidyl-lysine acetylation [GO:2000757]; negative regulation of transcription by RNA polymerase II [GO:0000122]; positive regulation of cell proliferation [GO:0008284]; positive regulation of epithelial to mesenchymal transition [GO:0010718]; positive regulation of interleukin-1 production [GO:0032732]; positive regulation of oligodendrocyte differentiation [GO:0048714]; positive regulation of proteolysis [GO:0045862]; positive regulation of receptor biosynthetic process [GO:0010870]; positive regulation of transcription by RNA polymerase II [GO:0045944]; positive regulation of tumor necrosis factor production [GO:0032760]; positive regulation	cytoplasm [GO:0005737]; ESC/E(Z) complex [GO:0035098]; NuRD complex [GO:0016581]; Sin3 complex [GO:0016580]	heat shock protein binding [GO:0031072]; histone deacetylase binding [GO:0042826]; metal ion binding [GO:0046872]; NAD-dependent histone deacetylase activity (H3-K14 specific) [GO:0032041]; NF-kappaB binding [GO:0051059]; promoter-specific chromatin binding [GO:1990841]; RNA polymerase II repressing transcription factor binding [GO:0001103]; sequence-specific DNA binding [GO:0043565]

Icam1	adhesion of symbiont to host [GO:0044406]; cell adhesion [GO:0007155]; cell adhesion mediated by integrin [GO:0033627]; cellular response to glucose stimulus [GO:0071333]; cellular response to leukemia inhibitory factor [GO:1990830]; establishment of endothelial barrier [GO:0061028]; leukocyte cell-cell adhesion [GO:0007159]; leukocyte migration [GO:0050900]; membrane to membrane docking [GO:0022614]; negative regulation of endothelial cell apoptotic process [GO:2000352]; negative regulation of extrinsic apoptotic signaling pathway via death domain receptors [GO:1902042]; positive regulation of cellular extravasation [GO:0002693]; positive regulation of ERK1 and ERK2 cascade [GO:0070374]; receptor-mediated virion attachment to host cell [GO:0046813]; regulation of cell adhesion [GO:0030155]; regulation of ruffle assembly [GO:1900027]; T cell activation via T cell receptor contact with antigen bound to MHC molecule on	external side of plasma membrane [GO:0009897]; extracellular exosome [GO:0070062]; extracellular matrix [GO:0031012]; extracellular space [GO:0005615]; immunological synapse [GO:0001772]; integral component of plasma membrane [GO:0005887]	integrin binding [GO:0005178]
Gla	oligosaccharide metabolic process [GO:0009311]	extracellular region [GO:0005576]; Golgi apparatus [GO:0005794]; lysosome [GO:0005764]	alpha-galactosidase activity [GO:0004557]; galactoside binding [GO:0016936]; protein homodimerization activity [GO:0042803]; receptor binding [GO:0005102]
Gns	glycosaminoglycan metabolic process [GO:0030203]	lysosome [GO:0005764]	metal ion binding [GO:0046872]; N-acetylglucosamine-6-sulfatase activity [GO:0008449]; sulfuric ester hydrolase activity [GO:0008484]

Grn	blastocyst hatching [GO:0001835]; embryo implantation [GO:0007566]; positive regulation of epithelial cell proliferation [GO:0050679]	endoplasmic reticulum [GO:0005783]; endosome [GO:0005768]; extracellular space [GO:0005615]; lysosome [GO:0005764]; mitochondrion [GO:0005739]	cytokine activity [GO:0005125]
Tfrc	[illegible] response to drug [GO:0035690]; cellular response to extracellular stimulus [GO:0031668]; cellular response to iron ion [GO:0071281]; cellular response to leukemia inhibitory factor [GO:1990830]; iron ion import [GO:0097286]; osteoclast differentiation [GO:0030316]; positive regulation of B cell proliferation [GO:0030890]; positive regulation of bone resorption [GO:0045780]; positive regulation of isotype switching [GO:0045830]; positive regulation of T cell proliferation [GO:0042102]; receptor internalization [GO:0031623]; transferrin transport [GO:0033572]	[illegible] surface [GO:0009986]; clathrin-coated pit [GO:0005905]; cytoplasmic vesicle [GO:0031410]; early endosome [GO:0005769]; endosome [GO:0005768]; endosome membrane [GO:0010008]; external side of plasma membrane [GO:0009897]; extracellular exosome [GO:0070062]; extracellular region [GO:0005576]; HFE-transferrin receptor complex [GO:1990712]; integral component of plasma membrane [GO:0005887]; melanosome [GO:0042470]; membrane [GO:0016020]; perinuclear region of cytoplasm [GO:0048471]; plasma membrane [GO:0005886]; recycling	double-stranded RNA binding [GO:0003725]; identical protein binding [GO:0042802]; iron ion transmembrane transporter activity [GO:0005381]; protein homodimerization activity [GO:0042803]; transferrin receptor activity [GO:0004998]; transferrin transmembrane transporter activity [GO:0033570]
Gnpnat1	cellular response to leukemia inhibitory factor [GO:1990830]; glucosamine metabolic process [GO:0006041]; liver development [GO:0001889]; N-acetylglucosamine metabolic process [GO:0006044]; UDP-N-acetylglucosamine biosynthetic process [GO:0006048]	endoplasmic reticulum-Golgi intermediate compartment [GO:0005793]; endosome membrane [GO:0010008]; Golgi apparatus [GO:0005794]; Golgi membrane [GO:0000139]; late endosome [GO:0005770]	glucosamine 6-phosphate N-acetyltransferase activity [GO:0004343]; identical protein binding [GO:0042802]; monosaccharide binding [GO:0048029]
Ctsd	autophagosome assembly [GO:0000045]; protein catabolic process [GO:0030163]; proteolysis [GO:0006508]	extracellular region [GO:0005576]; extracellular space [GO:0005615]; lysosome [GO:0005764]; melanosome [GO:0042470]; membrane raft [GO:0045121]; mitochondrion [GO:0005739]	aspartic-type endopeptidase activity [GO:0004190]; hydrolase activity [GO:0016787]; peptidase activity [GO:0008233]

Cryab	aging [GO:0007568]; apoptotic process involved in morphogenesis [GO:0060561]; camera-type eye development [GO:0043010]; cellular response to gamma radiation [GO:0071480]; lens development in camera-type eye [GO:0002088]; microtubule polymerization or depolymerization [GO:0031109]; muscle organ development [GO:0007517]; negative regulation of amyloid fibril formation [GO:1905907]; negative regulation of apoptotic process [GO:0043066]; negative regulation of cell growth [GO:0030308]; negative regulation of cysteine-type endopeptidase activity involved in apoptotic process [GO:0043154]; negative regulation of gene expression [GO:0010629]; negative regulation of intracellular transport [GO:0032387]; negative regulation of protein homooligomerization [GO:0032463]; negative regulation of reactive oxygen species metabolic process [GO:2000378]; protein folding [GO:0006457]; protein homooligomerization [GO:0051260]; protein stabilization [GO:0050821]; regulation of cell death [GO:0010941]; response to estradiol [GO:0032355]; response to hydrogen peroxide [GO:0042542]; response to hypoxia [GO:0001666]; stress-activated	actin filament bundle [GO:0032432]; axon [GO:0030424]; cardiac myofibril [GO:0097512]; cell surface [GO:0009986]; contractile fiber [GO:0043292]; cytoplasm [GO:0005737]; cytosol [GO:0005829]; dendritic spine [GO:0043197]; Golgi apparatus [GO:0005794]; M band [GO:0031430]; microtubule cytoskeleton [GO:0015630]; mitochondrion [GO:0005739]; myelin sheath [GO:0043209]; nucleus [GO:0005634]; perikaryon [GO:0043204]; plasma membrane [GO:0005886]; postsynaptic density [GO:0014069]; synaptic membrane [GO:0097060]; Z disc [GO:0030018]	amyloid-beta binding [GO:0001540]; identical protein binding [GO:0042802]; metal ion binding [GO:0046872]; microtubule binding [GO:0008017]; protein complex binding [GO:0032403]; protein homodimerization activity [GO:0042803]; structural constituent of eye lens [GO:0005212]; unfolded protein binding [GO:0051082]

Nptn	[illegible] homophilic cell adhesion via plasma membrane adhesion molecules [GO:0007156]; long-term synaptic potentiation [GO:0060291]; modulation of chemical synaptic transmission [GO:0050804]; positive regulation of cytosolic calcium ion concentration [GO:0007204]; positive regulation of fibroblast growth factor receptor signaling pathway [GO:0045743]; positive regulation of long-term neuronal synaptic plasticity [GO:0048170]; positive regulation of neuron projection development [GO:0010976]; positive regulation of protein phosphorylation [GO:0001934]; regulation of receptor localization to synapse [GO:1902683];	dendrite [GO:0030425]; integral component of membrane [GO:0016021]; presynaptic membrane [GO:0042734]	cell adhesion molecule binding [GO:0050839]; type 1 fibroblast growth factor receptor binding [GO:0005105]
Plod1	epidermis development [GO:0008544]; hydroxylysine biosynthetic process [GO:0046947]; peptidyl-lysine hydroxylation [GO:0017185]; response to hypoxia [GO:0001666]	catalytic complex [GO:1902494]; rough endoplasmic reticulum membrane [GO:0030867]	iron ion binding [GO:0005506]; L-ascorbic acid binding [GO:0031418]; procollagen-lysine 5-dioxygenase activity [GO:0008475]
Lnpep	cell-cell signaling [GO:0007267]; peptide catabolic process [GO:0043171]; protein catabolic process [GO:0030163]; regulation of blood pressure [GO:0008217]; SMAD protein signal transduction [GO:0060395]	cytoplasmic vesicle membrane [GO:0030659]; endomembrane system [GO:0012505]; integral component of membrane [GO:0016021]; intracellular [GO:0005622]; membrane [GO:0016020]; perinuclear region of cytoplasm [GO:0048471]; plasma membrane [GO:0005886]	aminopeptidase activity [GO:0004177]; metalloaminopeptidase activity [GO:0070006]; peptide binding [GO:0042277]; zinc ion binding [GO:0008270]

Itga5	cell-cell adhesion mediated by integrin [GO:0033631]; cell-substrate adhesion [GO:0031589]; cell-substrate junction assembly [GO:0007044]; endodermal cell differentiation [GO:0035987]; heterophilic cell-cell adhesion via plasma membrane cell adhesion molecules [GO:0007157]; heterotypic cell-cell adhesion [GO:0034113]; integrin-mediated signaling pathway [GO:0007229]; leukocyte cell-cell adhesion [GO:0007159]; memory [GO:0007613]; negative regulation of anoikis [GO:2000811]; positive regulation of cell migration [GO:0030335]; positive regulation of cell-substrate adhesion [GO:0010811]; positive regulation of peptidyl-tyrosine phosphorylation [GO:0050731]; positive regulation of sprouting angiogenesis [GO:1903672]; regulation of angiogenesis [GO:0045765]; wound healing,	cell-cell junction [GO:0005911]; cell surface [GO:0009986]; cytoplasmic vesicle [GO:0031410]; endoplasmic reticulum [GO:0005783]; external side of plasma membrane [GO:0009897]; focal adhesion [GO:0005925]; Golgi apparatus [GO:0005794]; integrin complex [GO:0008305]; plasma membrane [GO:0005886]; ruffle membrane [GO:0032587]; synapse [GO:0045202]	epidermal growth factor receptor binding [GO:0005154]; integrin binding [GO:0005178]; metal ion binding [GO:0046872]

Ero1a	animal organ senescence [GO:0010260]; brown fat cell differentiation [GO:0050873]; cell redox homeostasis [GO:0045454]; cellular protein modification process [GO:0006464]; cellular response to hypoxia [GO:0071456]; chaperone cofactor-dependent protein refolding [GO:0051085]; endoplasmic reticulum unfolded protein response [GO:0030968]; extracellular matrix organization [GO:0030198]; intrinsic apoptotic signaling pathway in response to endoplasmic reticulum stress [GO:0070059]; oxidation-reduction process [GO:0055114]; protein folding [GO:0006457]; protein folding in endoplasmic reticulum [GO:0034975]; protein maturation by protein folding [GO:0022417]; release of sequestered calcium ion into cytosol [GO:0051209]; response to endoplasmic reticulum stress [GO:0034976]	dendrite [GO:0030425]; endoplasmic reticulum [GO:0005783]; endoplasmic reticulum membrane [GO:0005789]; integral component of endoplasmic reticulum membrane [GO:0030176]	oxidoreductase activity [GO:0016491]; oxidoreductase activity, acting on a sulfur group of donors, disulfide as acceptor [GO:0016671]; protein disulfide isomerase activity [GO:0003756]; protein disulfide oxidoreductase activity [GO:0015035]
Itgb5	cell-matrix adhesion [GO:0007160]; epithelial cell-cell adhesion [GO:0090136]; integrin-mediated signaling pathway [GO:0007229]; stress fiber assembly [GO:0043149]; transforming growth factor beta receptor signaling pathway [GO:0007179]	cell leading edge [GO:0031252]; cell surface [GO:0009986]; focal adhesion [GO:0005925]; integrin alphav-beta5 complex [GO:0034684]; plasma membrane [GO:0005886]; receptor complex [GO:0043235]	integrin binding [GO:0005178]; receptor activity [GO:0004872]
Lrpap1	negative regulation of amyloid-beta clearance [GO:1900222]; negative regulation of cell death [GO:0060548]; negative regulation of protein binding [GO:0032091]; negative regulation of receptor internalization [GO:0002091]; negative regulation of very-low-density lipoprotein particle clearance [GO:0010916]; regulation of receptor-mediated endocytosis [GO:0048259]; transcytosis [GO:0045056]	cell surface [GO:0009986]; cis-Golgi network [GO:0005801]; endoplasmic reticulum [GO:0005783]; endoplasmic reticulum-Golgi intermediate compartment [GO:0005793]; endosome [GO:0005768]; endosome lumen [GO:0031904]; extracellular region [GO:0005576]; Golgi apparatus [GO:0005794]; Golgi lumen [GO:0005796]; plasma membrane [GO:0005886]; rough endoplasmic reticulum lumen [GO:0048237]	heparin binding [GO:0008201]; lipase binding [GO:0035473]; low-density lipoprotein particle receptor binding [GO:0050750]; receptor antagonist activity [GO:0048019]; very-low-density lipoprotein particle receptor binding [GO:0070326]
Psap	sphingolipid metabolic process [GO:0006665]	lysosome [GO:0005764]	

Lamc1	cell migration [GO:0016477]; extracellular matrix disassembly [GO:0022617]; hemidesmosome assembly [GO:0031581]; protein complex assembly [GO:0006461]; substrate adhesion-dependent cell spreading [GO:0034446]	laminin-1 complex [GO:0005606]	extracellular matrix structural constituent [GO:0005201]
Glt8d1	polysaccharide biosynthetic process [GO:0000271]	Golgi apparatus [GO:0005794]; integral component of membrane [GO:0016021]	transferase activity, transferring glycosyl groups [GO:0016757]
Itga3	cell adhesion [GO:0007155]; dendritic spine maintenance [GO:0097062]; exploration behavior [GO:0035640]; heart development [GO:0007507]; integrin-mediated signaling pathway [GO:0007229]; lung development [GO:0030324]; maternal process involved in female pregnancy [GO:0060135]; memory [GO:0007613]; mesodermal cell differentiation [GO:0048333]; negative regulation of cell projection organization [GO:0031345]; negative regulation of Rho protein signal transduction [GO:0035024]; nephron development [GO:0072006]; neuron migration [GO:0001764]; positive regulation of cell-substrate adhesion [GO:0010811]; positive regulation of epithelial cell migration [GO:0010634]; positive regulation of gene expression [GO:0010628]; positive regulation of neuron projection development [GO:0010976]; positive regulation of protein localization to plasma membrane [GO:1903078]; regulation of BMP signaling pathway [GO:0030510]; regulation of transforming growth factor beta receptor signaling pathway [GO:0017015]; regulation of Wnt signaling pathway [GO:0030111]; renal filtration [GO:0097205]; response to drug [GO:0042493];	basolateral plasma membrane [GO:0016323]; cell junction [GO:0030054]; cell periphery [GO:0071944]; cell surface [GO:0009986]; excitatory synapse [GO:0060076]; external side of plasma membrane [GO:0009897]; filopodium membrane [GO:0031527]; growth cone [GO:0030426]; integrin alpha3-beta1 complex [GO:0034667]; invadopodium membrane [GO:0071438]; perinuclear region of cytoplasm [GO:0048471]; plasma membrane [GO:0005886]; receptor complex [GO:0043235]; synapse [GO:0045202]; synaptic membrane [GO:0097060]	collagen binding [GO:0005518]; fibronectin binding [GO:0001968]; integrin binding [GO:0005178]; laminin binding [GO:0043236]; protease binding [GO:0002020]; protein domain specific binding [GO:0019904]; protein heterodimerization activity [GO:0046982]

Fuca2	fucose metabolic process [GO:0006004]; glycoside catabolic process [GO:0016139]; regulation of entry of bacterium into host cell [GO:2000535]; response to bacterium [GO:0009617]	extracellular space [GO:0005615]	alpha-L-fucosidase activity [GO:0004560]
Hyou1	cellular response to hypoxia [GO:0071456]; ER to Golgi vesicle-mediated transport [GO:0006888]; negative regulation of apoptotic process [GO:0043066]; negative regulation of endoplasmic reticulum stress-induced neuron intrinsic apoptotic signaling pathway [GO:1903382]; negative regulation of hypoxia-induced intrinsic apoptotic signaling pathway [GO:1903298]; response to endoplasmic reticulum stress [GO:0034976]; response to ischemia [GO:0002931]	endoplasmic reticulum chaperone complex [GO:0034663]; endoplasmic reticulum lumen [GO:0005788]; extracellular region [GO:0005576]	ATP binding [GO:0005524]
Hs2st1	heparan sulfate proteoglycan biosynthetic process, polysaccharide chain biosynthetic process [GO:0015014]; heparin metabolic process [GO:0030202]; ureteric bud formation [GO:0060676]	Golgi membrane [GO:0000139]; integral component of membrane [GO:0016021]	heparan sulfate 2-O-sulfotransferase activity [GO:0004394]

Tgfb2	[illegible] ascending aorta morphogenesis [GO:0035910]; atrial septum morphogenesis [GO:0060413]; atrial septum primum morphogenesis [GO:0003289]; atrioventricular valve morphogenesis [GO:0003181]; axon guidance [GO:0007411]; blood vessel development [GO:0001568]; blood vessel remodeling [GO:0001974]; BMP signaling pathway [GO:0030509]; cardiac epithelial to mesenchymal transition [GO:0060317]; cardiac left ventricle morphogenesis [GO:0003214]; cardiac muscle cell proliferation [GO:0060038]; cardiac right ventricle morphogenesis [GO:0003215]; cardioblast differentiation [GO:0010002]; cartilage condensation [GO:0001502]; cell-cell junction organization [GO:0045216]; cell cycle arrest [GO:0007050]; cell death [GO:0008219]; cell growth [GO:0016049]; cell migration [GO:0016477]; cell morphogenesis [GO:0000902]; collagen fibril organization [GO:0030199]; cranial skeletal system development [GO:1904888]; dopamine biosynthetic process [GO:0042416]; embryonic digestive tract development [GO:0048566]; embryonic limb morphogenesis [GO:0030326]; endocardial cushion fusion [GO:0003274]; endocardial cushion morphogenesis [GO:0003203]; epithelial to mesenchymal transition [GO:0001837]; extracellular matrix organization [GO:0030198]; extrinsic	axon [GO:0030424]; endosome [GO:0005768]; extracellular matrix [GO:0031012]; extracellular region [GO:0005576]; extracellular space [GO:0005615]; neuronal cell body [GO:0043025]	amyloid-beta binding [GO:0001540]; cytokine activity [GO:0005125]; growth factor activity [GO:0008083]; protein homodimerization activity [GO:0042803]; receptor binding [GO:0005102]; transforming growth factor beta receptor binding [GO:0005160]; type III transforming growth factor beta receptor binding [GO:0034714]; type II transforming growth factor beta receptor binding [GO:0005114]
Col6a1	cell adhesion [GO:0007155]; cellular response to amino acid stimulus [GO:0071230]; endodermal cell differentiation [GO:0035987]; protein heterotrimerization [GO:0070208]	collagen trimer [GO:0005581]; extracellular matrix [GO:0031012]; extracellular region [GO:0005576]; extracellular space [GO:0005615]; proteinaceous extracellular matrix [GO:0005578]; protein complex [GO:0043234]; sarcolemma [GO:0042383]	platelet-derived growth factor binding [GO:0048407]

Galnt2	immunoglobulin biosynthetic process [GO:0002378]; O-glycan processing [GO:0016266]; protein O-linked glycosylation [GO:0006493]; protein O-linked glycosylation via serine [GO:0018242]; protein O-linked glycosylation via threonine [GO:0018243]	extracellular space [GO:0005615]; Golgi apparatus [GO:0005794]; Golgi cisterna membrane [GO:0032580]; Golgi lumen [GO:0005796]; Golgi stack [GO:0005795]; integral component of membrane [GO:0016021]; perinuclear region of cytoplasm [GO:0048471]	carbohydrate binding [GO:0030246]; manganese ion binding [GO:0030145]; polypeptide N-acetylgalactosaminyltransferase activity [GO:0004653]
Lamb1	cell migration [GO:0016477]; embryo implantation [GO:0007566]; learning or memory [GO:0007611]; negative regulation of cell adhesion [GO:0007162]; neuronal-glial interaction involved in cerebral cortex radial glia guided migration [GO:0021812]; neuron projection development [GO:0031175]; odontogenesis [GO:0042476]; positive regulation of cell migration [GO:0030335]; substrate adhesion-dependent cell spreading [GO:0034446]	basal lamina [GO:0005605]; basement membrane [GO:0005604]; extracellular matrix [GO:0031012]; extracellular region [GO:0005576]; extracellular space [GO:0005615]; laminin-10 complex [GO:0043259]; laminin-1 complex [GO:0005606]; laminin-2 complex [GO:0005607]; laminin-8 complex [GO:0043257]; laminin complex [GO:0043256]; nucleus [GO:0005634]; perinuclear region of cytoplasm [GO:0048471]; proteinaceous extracellular matrix [GO:0005578]	enzyme binding [GO:0019899]; extracellular matrix structural constituent [GO:0005201]; glycosphingolipid binding [GO:0043208]; integrin binding [GO:0005178]
Ahsa2	response to stress [GO:0006950]		ATPase activator activity [GO:0001671]; chaperone binding [GO:0051087]
Crtap	chaperone-mediated protein folding [GO:0061077]; negative regulation of post-translational protein modification [GO:1901874]; peptidyl-proline hydroxylation to 3-hydroxy-L-proline [GO:0018400]; protein stabilization [GO:0050821]; spermatogenesis [GO:0007283]	endoplasmic reticulum [GO:0005783]; extracellular space [GO:0005615]; macromolecular complex [GO:0032991]; proteinaceous extracellular matrix [GO:0005578]	protein complex binding [GO:0032403]
Naglu	cerebellar Purkinje cell layer development [GO:0021680]; inner ear receptor cell development [GO:0060119]; locomotor rhythm [GO:0045475]; lysosome organization [GO:0007040]; middle ear morphogenesis [GO:0042474]; retinal rod cell development [GO:0046548]	extracellular exosome [GO:0070062]	
Tmem126a	optic nerve development [GO:0021554]	integral component of membrane [GO:0016021]; mitochondrial inner membrane [GO:0005743]; mitochondrion [GO:0005739]	

Ganab	carbohydrate metabolic process [GO:0005975]; N-glycan processing [GO:0006491]	endoplasmic reticulum [GO:0005783]; extracellular matrix [GO:0031012]; glucosidase II complex [GO:0017177]; Golgi apparatus [GO:0005794]; melanosome [GO:0042470]	carbohydrate binding [GO:0030246]; glucan 1,3-alpha-glucosidase activity [GO:0033919]; glucosidase activity [GO:0015926]
B4gat1	axon guidance [GO:0007411]; protein glycosylation [GO:0006486]; protein O-linked mannosylation [GO:0035269]	Golgi apparatus [GO:0005794]; Golgi membrane [GO:0000139]; integral component of membrane [GO:0016021]	glucuronosyltransferase activity [GO:0015020]; metal ion binding [GO:0046872]
Glb1	cellular carbohydrate metabolic process [GO:0044262]; galactose catabolic process [GO:0019388]; response to cortisone [GO:0051413]; response to Thyroglobulin triiodothyronine [GO:1904016]	cytoplasm [GO:0005737]; extracellular space [GO:0005615]; Golgi apparatus [GO:0005794]; intracellular membrane-bounded organelle [GO:0043231]; lysosome [GO:0005764]; vacuole [GO:0005773]	beta-galactosidase activity [GO:0004565]; galactoside binding [GO:0016936]; hydrolase activity [GO:0016787]
Ergic3	ER to Golgi vesicle-mediated transport [GO:0006888]	endoplasmic reticulum-Golgi intermediate compartment [GO:0005793]; endoplasmic reticulum-Golgi intermediate compartment membrane [GO:0033116]; endoplasmic reticulum membrane [GO:0005789]; Golgi apparatus [GO:0005794]; integral component of membrane	
Ostc	protein glycosylation [GO:0006486]	integral component of membrane [GO:0016021]; oligosaccharyltransferase complex [GO:0008250]	
Abcd3	fatty acid beta-oxidation [GO:0006635]; fatty acid biosynthetic process [GO:0006633]; peroxisomal long-chain fatty acid import [GO:0015910]; peroxisome organization [GO:0007031]; response to drug [GO:0042493]; response to organic cyclic compound [GO:0014070]; very long-chain fatty acid catabolic process [GO:0042760]	integral component of membrane [GO:0016021]; intracellular membrane-bounded organelle [GO:0043231]; mitochondrial inner membrane [GO:0005743]; mitochondrion [GO:0005739]; peroxisomal matrix [GO:0005782]; peroxisomal membrane [GO:0005778]; peroxisome [GO:0005777]	ATPase activity [GO:0016887]; ATPase activity, coupled to transmembrane movement of substances [GO:0042626]; ATP binding [GO:0005524]; long-chain fatty acid transporter activity [GO:0005324]; protein homodimerization activity [GO:0042803]; protein self-association [GO:0043621]

Pla2g15	ceramide metabolic process [GO:0006672]; fatty acid metabolic process [GO:0006631]; glycerophospholipid metabolic process [GO:0006650]; phosphatidylcholine catabolic process [GO:0034638]; phosphatidylethanolamine catabolic process [GO:0046338]	extracellular space [GO:0005615]; intracellular membrane-bounded organelle [GO:0043231]; lysosome [GO:0005764]; membrane [GO:0016020]; mitochondrion [GO:0005739]; nucleoplasm [GO:0005654]	calcium-independent phospholipase A2 activity [GO:0047499]; O-acyltransferase activity [GO:0008374]
Gusb	carbohydrate metabolic process [GO:0005975]	endoplasmic reticulum [GO:0005783]; extracellular space [GO:0005615]; intracellular membrane-bounded organelle [GO:0043231]; lysosome [GO:0005764]	beta-glucuronidase activity [GO:0004566]; hydrolase activity [GO:0016787]; protein domain specific binding [GO:0019904]; receptor binding [GO:0005102]
Pofut1	angiogenesis [GO:0001525]; fucose metabolic process [GO:0006004]; heart development [GO:0007507]; nervous system development [GO:0007399]; Notch signaling pathway [GO:0007219]; protein O-linked fucosylation [GO:0036066]; protein O-linked glycosylation [GO:0006493]; regulation of Notch signaling pathway [GO:0008593]; somitogenesis	endoplasmic reticulum [GO:0005783]; membrane [GO:0016020]	fucosyltransferase activity [GO:0008417]; peptide-O-fucosyltransferase activity [GO:0046922]
Galnt1	protein O-linked glycosylation [GO:0006493]; protein O-linked glycosylation via serine [GO:0018242]; protein O-linked glycosylation via threonine [GO:0018243]	extracellular region [GO:0005576]; Golgi cisterna membrane [GO:0032580]; integral component of membrane [GO:0016021]; perinuclear region of cytoplasm [GO:0048471]	carbohydrate binding [GO:0030246]; manganese ion binding [GO:0030145]; polypeptide N-acetylgalactosaminyltransferase activity [GO:0004653]

Sptbn1	actin filament capping [GO:[illegible]]; common partner SMAD protein phosphorylation [GO:0007182]; Golgi to plasma membrane protein transport [GO:0043001]; membrane assembly [GO:0071709]; mitotic cytokinesis [GO:0000281]; plasma membrane organization [GO:0007009]; positive regulation of interleukin-2 secretion [GO:1900042]; positive regulation of protein localization to plasma membrane [GO:1903078]; protein localization to plasma membrane [GO:0072659]; regulation of protein localization to plasma membrane [GO:1903076]; SMAD protein	axolemma [GO:0030673]; cortical cytoskeleton [GO:0030863]; cuticular plate [GO:0032437]; cytoplasm [GO:0005737]; endomembrane system [GO:0012505]; M band [GO:0031430]; membrane [GO:0016020]; nucleolus [GO:0005730]; nucleus [GO:0005634]; plasma membrane [GO:0005886]; postsynaptic density [GO:0014069]; protein complex [GO:0043234]; spectrin [GO:0008091]	actin binding [GO:0003779]; ankyrin binding [GO:0030506]; cadherin binding [GO:0045296]; calmodulin binding [GO:0005516]; GTPase binding [GO:0051020]; phospholipid binding [GO:0005543]; protein complex binding [GO:0032403]; structural constituent of cytoskeleton [GO:0005200]
Epdr1	cell-matrix adhesion [GO:0007160]	extracellular space [GO:0005615]; lysosome [GO:0005764]	calcium ion binding [GO:0005509]

Itga6	amelogenesis [GO:0097186]; brown fat cell differentiation [GO:0050873]; cell adhesion mediated by integrin [GO:0033627]; cell-cell adhesion [GO:0098609]; cell-matrix adhesion [GO:0007160]; cell-substrate adhesion [GO:0031589]; cellular response to extracellular stimulus [GO:0031668]; cellular response to organic cyclic compound [GO:0071407]; digestive tract development [GO:0048565]; ectodermal cell differentiation [GO:0010668]; filopodium assembly [GO:0046847]; integrin-mediated signaling pathway [GO:0007229]; leukocyte migration [GO:0050900]; nail development [GO:0035878]; negative regulation of extrinsic apoptotic signaling pathway [GO:2001237]; odontogenesis of dentin-containing tooth [GO:0042475]; positive regulation of apoptotic process [GO:0043065]; positive regulation of cell-cell adhesion [GO:0022409]; positive regulation of cell migration [GO:0030335]; positive regulation of cell-substrate adhesion [GO:0010811]; positive regulation of GTPase activity [GO:0043547]; positive regulation of phosphorylation [GO:0042327]; positive regulation of transcription by RNA polymerase II [GO:0045944]; renal system	basal part of cell [GO:0045178]; basal plasma membrane [GO:0009925]; basement membrane [GO:0005604]; basolateral plasma membrane [GO:0016323]; cell-cell adherens junction [GO:0005913]; cell surface [GO:0009986]; external side of plasma membrane [GO:0009897]; filopodium [GO:0030175]; hemidesmosome [GO:0030056]; integrin alpha6-beta4 complex [GO:0034676]; integrin complex [GO:0008305]; plasma membrane [GO:0005886]	cadherin binding [GO:0045296]; insulin-like growth factor I binding [GO:0031994]; integrin binding [GO:0005178]; laminin binding [GO:0043236]; metal ion binding [GO:0046872]; neuregulin binding [GO:0038132]
Pld3	lipid catabolic process [GO:0016042]	endoplasmic reticulum membrane [GO:0005789]; integral component of membrane [GO:0016021]	N-acylphosphatidylethanolamine-specific phospholipase D activity [GO:0070290]; phospholipase D

P4ha1	collagen fibril organization [GO:0030199]; peptidyl-proline hydroxylation to 4-hydroxy-L-proline [GO:0018401]	endoplasmic reticulum [GO:0005783]; endoplasmic reticulum lumen [GO:0005788]; intracellular membrane-bounded organelle [GO:0043231]; mitochondrion [GO:0005739]; procollagen-proline 4-dioxygenase complex [GO:0016222]	identical protein binding [GO:0042802]; iron ion binding [GO:0005506]; L-ascorbic acid binding [GO:0031418]; oxidoreductase activity, acting on single donors with incorporation of molecular oxygen, incorporation of two atoms of oxygen [GO:0016702]; procollagen-proline 4-dioxygenase activity [GO:0004656]
Plod2	hydroxylysine biosynthetic process [GO:0046947]; peptidyl-lysine hydroxylation [GO:0017185]; response to hypoxia [GO:0001666]	endoplasmic reticulum [GO:0005783]	iron ion binding [GO:0005506]; L-ascorbic acid binding [GO:0031418]; procollagen-lysine 5-dioxygenase activity [GO:0008475]
Ncstn	amyloid-beta formation [GO:0034205]; amyloid-beta metabolic process [GO:0050435]; amyloid precursor protein metabolic process [GO:0042982]; epithelial cell proliferation [GO:0050673]; membrane protein ectodomain proteolysis [GO:0006509]; myeloid cell homeostasis [GO:0002262]; Notch receptor processing [GO:0007220]; Notch signaling pathway [GO:0007219]; positive regulation of catalytic activity [GO:0043085]; protein processing	cytoplasmic vesicle membrane [GO:0030659]; endoplasmic reticulum [GO:0005783]; gamma-secretase complex [GO:0070765]; Golgi apparatus [GO:0005794]; integral component of membrane [GO:0016021]; integral component of plasma membrane [GO:0005887]; lysosomal membrane [GO:0005765]; melanosome [GO:0042470]; membrane [GO:0016020]; plasma membrane [GO:0005886]	endopeptidase activity [GO:0004175]

Lgmn	negative regulation of ERBB signaling pathway [GO:1901185]; negative regulation of multicellular organism growth [GO:0040015]; negative regulation of neuron apoptotic process [GO:0043524]; proteolysis [GO:0006508]; proteolysis involved in cellular protein catabolic process [GO:0051603]; receptor catabolic process [GO:0032801]; renal system process [GO:0003014]; response to acidic pH [GO:0010447]; vacuolar protein processing [GO:0006624]	apical part of cell [GO:0045177]; late endosome [GO:0005770]; lysosome [GO:0005764]	cysteine-type endopeptidase activity [GO:0004197]; peptidase activity [GO:0008233]
Tubb3	microtubule-based process [GO:0007017]	microtubule [GO:0005874]	GTPase activity [GO:0003924]; GTP binding [GO:0005525]; structural constituent of cytoskeleton [GO:0005200]
P4ha1	collagen fibril organization [GO:0030199]; peptidyl-proline hydroxylation to 4-hydroxy-L-proline [GO:0018401]	procollagen-proline 4-dioxygenase complex [GO:0016222]	iron ion binding [GO:0005506]; L-ascorbic acid binding [GO:0031418]; oxidoreductase activity, acting on single donors with incorporation of molecular oxygen, incorporation of two atoms of oxygen [GO:0016702]; procollagen-proline 4-dioxygenase activity [GO:0004656]
Pcyox1	prenylated protein catabolic process [GO:0030327]; prenylcysteine catabolic process [GO:0030328]	lysosome [GO:0005764]; plasma membrane [GO:0005886]; vacuolar membrane [GO:0005774]; very-low-density lipoprotein particle [GO:0034361]	chloride-transporting ATPase activity [GO:0008555]; prenylcysteine oxidase activity [GO:0001735]

Tnc	[GO:0060447]; cell adhesion [GO:0007155]; cellular response to prostaglandin D stimulus [GO:0071799]; cellular response to retinoic acid [GO:0071300]; cellular response to vitamin D [GO:0071305]; mesenchymal-epithelial cell signaling involved in prostate gland development [GO:0060739]; negative regulation of cell adhesion [GO:0007162]; neuromuscular junction development [GO:0007528]; odontogenesis of dentin-containing tooth [GO:0042475]; peripheral nervous system axon regeneration [GO:0014012]; positive regulation of cell proliferation [GO:0008284]; positive regulation of gene expression [GO:0010628]; prostate gland epithelium morphogenesis [GO:0060740]; response to ethanol [GO:0045471]; response to fibroblast growth factor [GO:0071774]; response to mechanical stimulus [GO:0009612];	basement membrane [GO:0005604]; extracellular matrix [GO:0031012]; extracellular region [GO:0005576]; extracellular space [GO:0005615]; interstitial matrix [GO:0005614]	fibronectin binding [GO:0001968]; syndecan binding [GO:0045545]
Itgav	cell adhesion [GO:0007155]; integrin-mediated signaling pathway [GO:0007229]	integrin complex [GO:0008305]	
Nomo1		endoplasmic reticulum membrane [GO:0005789]; integral component of membrane [GO:0016021]	carbohydrate binding [GO:0030246]
