# Supplementary material for: TGFβ attenuates cartilage extracellular matrix degradation via enhancing FBXO6-mediated MMP14 ubiquitination
Source: Ann Rheum Dis. 2020 May 14;79(8):1111–20. doi: 10.1136/annrheumdis-2019-216911 (PMC7392491; doi:10.1136/annrheumdis-2019-216911)
Supplement: Supplementary data [file annrheumdis-2019-216911supp006.pdf]

Protein ID	UniProtKB.ID	control	ko	P Value	Fold Change Ratio	Fold Change
7821	BST2_MOUSE	3696667	4833.333	0.000413	764.8275862	764.83
4657	ISG15_MOUSE	17733333	81033.33	0.001401	218.8399835	218.84
7163	PRELP_MOUSE	2326667	31433.33	0.000159	74.01908802	74.02
3603	PSB8_MOUSE	3100000	56900	0.002101	54.48154657	54.48
2602	Q3TBA3_MOUSE	10880000	238666.7	0.005524	45.58659218	45.59
12762	CAH9_MOUSE	4753333	118800	0.008963	40.01122334	40.01
2585	ICAM1_MOUSE	28600000	716333.3	0.002142	39.92554677	39.93
7853	UB2L6_MOUSE	8503333	232333.3	0.011093	36.59971306	36.6
4502	OAS1A_MOUSE	5840000	163366.7	0.00187	35.74780657	35.75
3010	J7NUP1_MOUSE	19233333	619333.3	0.001213	31.05489774	31.05
413	IFIT1_MOUSE	56200000	1906667	5.24E-05	29.47552448	29.48
4010	SAM9L_MOUSE	3730000	157133.3	0.025518	23.73780229	23.74
12576	PTGES_MOUSE	6423333	301000	0.00078	21.33997785	21.34
5961	CO4B_MOUSE	2730000	133833.3	0.000277	20.3985056	20.4
1908	Q9QXJ2_MOUSE	13033333	794000	1.8E-05	16.4147775	16.41
6404	TAP2_MOUSE	5576667	382000	0.002617	14.59860384	14.6
2615	Q91Z40_MOUSE	4390000	329666.7	1.06E-05	13.31648129	13.32
1898	HA11_MOUSE	25000000	2040000	0.000104	12.25490196	12.25
3883	B1AQR8_MOUSE	15033333	1245667	3.14E-05	12.06850415	12.07
9136	NOS2_MOUSE	1396667	120266.7	0.001689	11.61308204	11.61
1543	E9QKL6_MOUSE	5963333	541000	4.25E-06	11.02279729	11.02
3042	TPSN_MOUSE	16833333	1603333	4.68E-06	10.4989605	10.5
3402	GBP2_MOUSE	22433333	2260667	0.006141	9.923326452	9.92
7747	A0A087WRP7_MOU	48700000	5093333	0.000356	9.561518325	9.56
4424	CASP8_MOUSE	14666667	1537667	1.36E-05	9.538261435	9.54
13067	IPRI_MOUSE	1490000	156666.7	0.003829	9.510638298	9.51
2768	IIGP1_MOUSE	11210000	1250667	0.024694	8.963219616	8.96
6988	SO2A1_MOUSE	4000000	457000	0.000371	8.75273523	8.75
1551	XDH_MOUSE	13500000	1580000	0.003614	8.544303797	8.54
5394	IFIH1_MOUSE	8093333	997666.7	0.000727	8.112261945	8.11
8146	PGH1_MOUSE	3163333	390433.3	0.008497	8.102108768	8.1
6717	VNN1_MOUSE	5216667	644666.7	2.23E-05	8.092037229	8.09
230	Q8C3V4_MOUSE	60666667	8083333	6E-05	7.505154639	7.51
2011	E2AK2_MOUSE	19866667	2676667	1.83E-05	7.422166874	7.42
6444	FBLN3_MOUSE	1836667	250333.3	0.000269	7.336884154	7.34
4922	DCXR_MOUSE	4066667	564333.3	0.000378	7.206142942	7.21
7058	CAH13_MOUSE	10276667	1456667	8.71E-05	7.054919908	7.05
1208	LG3BP_MOUSE	37300000	5423333	6.81E-05	6.877688998	6.88
1351	DDX58_MOUSE	25600000	3736667	0.000147	6.85102587	6.85
12511	A0A087WNS9_MOU	5333333	790333.3	0.011845	6.748207507	6.75
3071	CKAP2_MOUSE	4000000	600333.3	0.039806	6.662965019	6.66
7641	ERRFI_MOUSE	4090000	651000	0.012548	6.282642089	6.28
2578	HA1B_MOUSE	12500000	2000000	0.006436	6.25	6.25
1696	IFI5B_MOUSE	5290000	883333.3	0.008982	5.988679245	5.99
5322	LOXL3_MOUSE	3153333	557666.7	0.000467	5.654512851	5.65
1035	G3X9S2_MOUSE	28266667	5190000	0.000608	5.446371227	5.45
3505	SBP1_MOUSE	13533333	2540000	1.59E-05	5.32808399	5.33
4692	CCND1_MOUSE	10513333	1993333	0.03024	5.274247492	5.27
8403	FBLN4_MOUSE	2513333	485666.7	0.00378	5.175017159	5.18

6567	B2MG_MOUSE	30433333	6400000	0.002327	4.755208333	4.76
4378	D3Z450_MOUSE	68900000	14533333	0.001012	4.740825688	4.74
581	NEST_MOUSE	14700000	3170000	0.000174	4.637223975	4.64
6941	A2AS05_MOUSE	512000	110333.3	0.03437	4.640483384	4.64
7684	MT1_MOUSE	83033333	18033333	0.026146	4.604436229	4.6
3613	MK09_MOUSE	13333333	2913333	0.001955	4.576659039	4.58
2290	ALDOC_MOUSE	10800000	2446667	0.000327	4.414168937	4.41
5189	MLKL_MOUSE	5306667	1216000	0.000496	4.364035088	4.36
8834	LATS1_MOUSE	2950000	690666.7	0.000452	4.271235521	4.27
6327	NGAL_MOUSE	11600000	2810000	0.000513	4.128113879	4.13
5218	GUAD_MOUSE	4083333	996000	0.01626	4.099732262	4.1
8570	D3Z3L3_MOUSE	1144333	279000	0.002614	4.101553166	4.1
8242	F8VPL2_MOUSE	1856667	469000	9.27E-05	3.958777541	3.96
3176	DOCK1_MOUSE	21500000	5483333	0.021406	3.920972644	3.92
5924	TIMP1_MOUSE	5846667	1515000	0.008354	3.859185919	3.86
4749	AGAL_MOUSE	9903333	2596667	0.001811	3.813863928	3.81
1191	PLIN2_MOUSE	41100000	10843333	1.37E-05	3.790347372	3.79
9234	A0A1D5RLM8_MOU	874333.3	230766.7	0.030915	3.788819876	3.79
10811	A2AKH7_MOUSE	2630000	710666.7	0.00727	3.700750469	3.7
6855	BI2L1_MOUSE	2816667	762533.3	0.022846	3.693827592	3.69
2263	GLUCM_MOUSE	10230000	2790000	0.003291	3.666666667	3.67
5473	STEA4_MOUSE	6613333	1800000	7.57E-07	3.674074074	3.67
46	TBA4A_MOUSE	9566667	2633333	0.000103	3.632911392	3.63
4792	ECE1_MOUSE	4536667	1250000	0.001323	3.629333333	3.63
552	AMPD3_MOUSE	28633333	7913333	0.000938	3.618365628	3.62
10632	TNR23_MOUSE	600333.3	171000	0.000115	3.510721248	3.51
378	A0A171EBL2_MOU	18033333	5196667	0.000139	3.470173188	3.47
6434	NUA4L_MOUSE	22833333	6573333	4.23E-05	3.473630832	3.47
5455	TRBM_MOUSE	8426667	2440000	0.000478	3.453551913	3.45
3653	PAI1_MOUSE	4770000	1406667	0.001	3.390995261	3.39
2718	PML_MOUSE	8330000	2516667	0.035527	3.309933775	3.31
3822	TOR3A_MOUSE	10500000	3183333	0.01104	3.298429319	3.3
12618	E9Q2S9_MOUSE	2786667	873666.7	0.00151	3.189622282	3.19
8559	PPM1F_MOUSE	4710000	1496667	3.05E-05	3.146993318	3.15
5555	ZNFX1_MOUSE	1316667	421000	0.022589	3.127474268	3.13
3694	F6XWD4_MOUSE	6663333	2166667	0.01483	3.075384615	3.08
5089	GLRX1_MOUSE	74400000	24266667	0.001421	3.065934066	3.07
3957	GSDMD_MOUSE	3876667	1280000	0.011648	3.028645833	3.03
6736	CHSP1_MOUSE	34633333	11466667	0.020435	3.020348837	3.02
8228	A0A0U1RPJ4_MOU	1523333	520666.7	0.025188	2.925736236	2.93
3531	TOIP2_MOUSE	7240000	2500000	0.012769	2.896	2.9
2381	GSH1_MOUSE	10223333	3533333	0.005221	2.893396226	2.89
11663	SCLY_MOUSE	715000	247900	0.044656	2.884227511	2.88
4211	F162A_MOUSE	1.88E+08	65633333	0.000123	2.86896902	2.87
1321	ASSY_MOUSE	50600000	18166667	0.00042	2.785321101	2.79
1403	RIR2_MOUSE	24766667	8886667	0.00537	2.786946737	2.79
2323	ILEUA_MOUSE	17100000	6130000	0.000377	2.789559543	2.79
977	KANK2_MOUSE	21433333	7736667	0.00072	2.770357604	2.77
6347	IFM3_MOUSE	1.27E+08	46000000	0.001586	2.767391304	2.77
3678	CB39L_MOUSE	1886667	686666.7	0.010956	2.747572816	2.75

7770	CRIP1_MOUSE	38933333	14233333	0.000131	2.735362998	2.74
6403	AL4A1_MOUSE	3543333	1298667	0.04793	2.728439425	2.73
3349	TGM2_MOUSE	6000000	2203333	0.012971	2.723146747	2.72
7666	PRP4B_MOUSE	10700000	3936667	0.007433	2.718035563	2.72
3850	ANLN_MOUSE	5530000	2040000	0.000726	2.710784314	2.71
796	AAK1_MOUSE	12500000	4623333	0.045994	2.703677001	2.7
7904	FUCO2_MOUSE	2496667	935333.3	0.033802	2.669280114	2.67
3679	F2Z3U3_MOUSE	16566667	6326667	0.018201	2.618545838	2.62
4167	PTX3_MOUSE	6290000	2450000	0.004839	2.567346939	2.57
1987	HMOX1_MOUSE	1.18E+08	45966667	0.002638	2.559100798	2.56
8319	BID_MOUSE	7290000	2880000	0.012982	2.53125	2.53
331	RTN4_MOUSE	27700000	11200000	0.001198	2.473214286	2.47
915	SNX9_MOUSE	35200000	14333333	2.46E-05	2.455813953	2.46
4753	HPCL1_MOUSE	8930000	3666667	0.026232	2.435454545	2.44
4912	STOM_MOUSE	12033333	4933333	2.17E-05	2.439189189	2.44
3324	Q6P6I8_MOUSE	7010000	2880000	0.021052	2.434027778	2.43
4655	J3QP71_MOUSE	1323333	551333.3	0.013837	2.400241838	2.4
3943	MOT4_MOUSE	50666667	21100000	0.000761	2.401263823	2.4
4382	MINK1_MOUSE	373666.7	155900	0.026436	2.396835578	2.4
8523	FRMD8_MOUSE	1770000	737666.7	0.001623	2.39945775	2.4
8451	PHLA3_MOUSE	4080000	1713333	0.018766	2.381322957	2.38
6375	TR10B_MOUSE	2996667	1270000	0.000498	2.359580052	2.36
6057	ANTR1_MOUSE	2496667	1063667	0.048466	2.347226575	2.35
1287	TRI25_MOUSE	13500000	5776667	0.00352	2.336987882	2.34
2513	STXB1_MOUSE	12066667	5166667	0.000988	2.335483871	2.34
4457	FSTL1_MOUSE	13600000	5873333	0.003075	2.315550511	2.32
3136	ACSL4_MOUSE	6620000	2870000	0.000579	2.306620209	2.31
3774	MFGM_MOUSE	7006667	3026667	0.0084	2.314977974	2.31
9169	DGAT1_MOUSE	3516667	1520000	0.043471	2.313596491	2.31
4174	IBP7_MOUSE	4523333	1966667	0.000394	2.3	2.3
3206	E9PZD2_MOUSE	5083333	2223333	0.013104	2.286356822	2.29
8259	A0A1L1ST61_MOU!	19833333	8676667	0.029288	2.285824049	2.29
10331	SDC3_MOUSE	3263333	1423333	0.004299	2.292740047	2.29
9013	Q8BVA0_MOUSE	14333333	6330000	0.002648	2.264349658	2.26
3688	AMPN_MOUSE	3590000	1610333	0.034343	2.229352101	2.23
5223	ABCF3_MOUSE	5680000	2563333	2.89E-06	2.215864759	2.22
1051	KCRB_MOUSE	74133333	33600000	0.00082	2.206349206	2.21
5810	CATH_MOUSE	5470000	2483333	0.003634	2.202684564	2.2
8901	GLNA_MOUSE	2223333	1016000	0.017321	2.18832021	2.19
5212	D3Z125_MOUSE	25033333	11480000	0.042691	2.180603949	2.18
2090	F8WI14_MOUSE	15666667	7216667	1.37E-05	2.170900693	2.17
6817	SNP29_MOUSE	4850000	2236667	0.028614	2.168405365	2.17
8770	D3YU60_MOUSE	11133333	5123333	0.000387	2.173064411	2.17
3231	GLGB_MOUSE	8970000	4166667	0.010979	2.1528	2.15
5732	HSPB7_MOUSE	8100000	3766667	0.000161	2.150442478	2.15
8716	TYB10_MOUSE	34633333	16333333	0.021226	2.120408163	2.12
7712	A0A1L1STY4_MOU!	898666.7	424333.3	0.00309	2.117831893	2.12
1029	ANXA8_MOUSE	1.04E+08	49400000	6.51E-06	2.109311741	2.11
1862	TFR1_MOUSE	12900000	6153333	0.005217	2.096424702	2.1
4671	LOXL4_MOUSE	4336667	2073333	0.030613	2.091639871	2.09

1984	A0A0R4J008_MOUS	12800000	6140000	0.002056	2.084690554	2.08
8252	NOL11_MOUSE	1926667	928000	0.007039	2.076149425	2.08
752	GRN_MOUSE	1.85E+08	89500000	0.003815	2.067783985	2.07
6966	GTR1_MOUSE	26666667	12966667	0.006785	2.05655527	2.06
8225	A0A1B0GSI7_MOUS	3116667	1520000	0.00381	2.050438596	2.05
5545	AQP1_MOUSE	38000000	18700000	0.008161	2.032085561	2.03
7938	TCAF2_MOUSE	1613333	796333.3	0.033995	2.025952281	2.03
539	PYGL_MOUSE	33933333	16800000	0.022713	2.01984127	2.02
3180	SEH1_MOUSE	15633333	7796667	0.02205	2.005130398	2.01
8714	K2013_MOUSE	2566667	1279667	0.022347	2.005730659	2.01
3292	NDRG2_MOUSE	12566667	6276667	0.000182	2.00212427	2
3120	PSME2_MOUSE	47400000	23800000	0.016293	1.991596639	1.99
2874	EMB_MOUSE	27866667	14000000	0.000246	1.99047619	1.99
5796	CD44_MOUSE	38766667	19433333	0.002988	1.994854202	1.99
3182	KAD1_MOUSE	70400000	35766667	0.032606	1.968313141	1.97
3736	CD109_MOUSE	3016667	1530000	0.000201	1.97167756	1.97
4747	CRYAB_MOUSE	28533333	14566667	0.007002	1.958810069	1.96
6803		1783333	910333.3	0.000802	1.958989381	1.96
3780	GAPD1_MOUSE	4643333	2370000	0.027368	1.959212377	1.96
962	HXK2_MOUSE	14633333	7513333	0.017846	1.947648625	1.95
1563	FND3A_MOUSE	16566667	8486667	0.003223	1.952081697	1.95
3118	M3K20_MOUSE	9010000	4616667	0.021572	1.951624549	1.95
7093	CDN1C_MOUSE	7723333	3966667	0.000616	1.947058824	1.95
1747	MP2K1_MOUSE	46266667	23866667	0.000745	1.938547486	1.94
2182	G3UXZ5_MOUSE	37800000	19533333	0.007301	1.935153584	1.94
5969	PEAK1_MOUSE	5686667	2936667	0.032376	1.936435868	1.94
1067	GDN_MOUSE	41033333	21233333	0.00368	1.932496075	1.93
3455	NQO1_MOUSE	25033333	12966667	0.001342	1.93059126	1.93
6764	GSTT3_MOUSE	5573333	2886667	0.020162	1.930715935	1.93
600	AL1A7_MOUSE	19133333	9960000	0.000636	1.921017403	1.92
3204	GDIR2_MOUSE	50866667	26533333	0.015929	1.917085427	1.92
4337	NPC2_MOUSE	2.47E+08	1.29E+08	0.006266	1.907474227	1.91
5538	CXA1_MOUSE	13966667	7316667	7.67E-05	1.908883827	1.91
8368	XYLK_MOUSE	1473333	770666.7	0.016659	1.911764706	1.91
1705	PGAM1_MOUSE	7.24E+08	3.84E+08	0.000118	1.886707211	1.89
3320	Q8C253_MOUSE	1.57E+08	83933333	0.011204	1.875297855	1.88
5158	PIGT_MOUSE	6430000	3426667	0.02517	1.876459144	1.88
549	HS71B_MOUSE	24066667	12866667	0.000123	1.870466321	1.87
4906	FRIH_MOUSE	19133333	10233333	0.00448	1.86970684	1.87
4305	CFA36_MOUSE	6240000	3343333	0.01363	1.866400798	1.87
9322	ATG2B_MOUSE	568666.7	303333.3	0.021958	1.874725275	1.87
3955	BASI_MOUSE	15533333	8333333	0.029885	1.864	1.86
1202	PNPH_MOUSE	1.06E+08	57300000	0.000797	1.852239674	1.85
5277	ACAP2_MOUSE	3363333	1820000	0.000228	1.847985348	1.85
4490	NEMF_MOUSE	6303333	3416667	0.00502	1.844878049	1.84
8220	HMGA2_MOUSE	1.52E+08	82933333	0.00323	1.828376206	1.83
7642	E9PUE7_MOUSE	1750000	958000	0.011409	1.826722338	1.83
9111	TXNIP_MOUSE	4630000	2533333	0.036298	1.827631579	1.83
1804	IF4G3_MOUSE	5563333	3056667	0.023108	1.820065431	1.82
1553	RIPK3_MOUSE	20800000	11400000	0.011141	1.824561404	1.82

7946		83100000	45566667	0.016077	1.823701536	1.82
3514	F8VQK5_MOUSE	4550000	2496667	0.011277	1.822429907	1.82
133	KINH_MOUSE	41366667	22800000	0.001437	1.814327485	1.81
6730	CD81_MOUSE	46000000	25400000	6.18E-05	1.811023622	1.81
8778	E0CZ22_MOUSE	1012000	560333.3	0.045425	1.806067817	1.81
12967	SGMR1_MOUSE	3680000	2040000	0.000754	1.803921569	1.8
408	SPB6_MOUSE	1.31E+08	72933333	0.009083	1.791133455	1.79
1376	B1AZ46_MOUSE	8436667	4723333	0.015946	1.78616796	1.79
2746	DHRS9_MOUSE	17966667	10033333	0.007209	1.790697674	1.79
9745	Q8K094_MOUSE	5456667	3040000	0.000207	1.79495614	1.79
3776	3BP1_MOUSE	2786667	1566667	0.024943	1.778723404	1.78
9763	NECP1_MOUSE	5290000	2973333	0.005798	1.779147982	1.78
8200	MDC1_MOUSE	1723333	966000	0.011595	1.783988958	1.78
957	F7AA26_MOUSE	21200000	11976667	0.021711	1.770108544	1.77
6394	A0A0A0MQ90_MO	47133333	26666667	0.003926	1.7675	1.77
8440	G5E8C3_MOUSE	4960000	2810000	0.000698	1.765124555	1.77
8127	DYLT3_MOUSE	3753333	2130000	0.001588	1.762128326	1.76
270	GELS_MOUSE	2.17E+08	1.24E+08	0.000856	1.746112601	1.75
7680	PGPI_MOUSE	6410000	3663333	7.46E-05	1.74977252	1.75
388	INF2_MOUSE	16800000	9643333	0.043871	1.742136191	1.74
1435	SAM50_MOUSE	12633333	7256667	0.002165	1.740927882	1.74
13267	PTMA_MOUSE	53166667	30500000	0.000963	1.743169399	1.74
10101	F107B_MOUSE	4213333	2416667	0.006965	1.743448276	1.74
26	LDHA_MOUSE	1.32E+09	7.64E+08	0.01595	1.727256869	1.73
2416	PGP_MOUSE	23300000	13500000	0.006552	1.725925926	1.73
2594	PAK3_MOUSE	1860000	1076333	0.019449	1.728089192	1.73
6715	D3Z7F7_MOUSE	6740000	3886667	0.003949	1.734133791	1.73
7965	ENPP5_MOUSE	2420000	1400000	0.047084	1.728571429	1.73
3492	PAR12_MOUSE	4253333	2473333	0.000281	1.71967655	1.72
1011	CATB_MOUSE	1.45E+08	84800000	0.004423	1.706367925	1.71
1246	ESTD_MOUSE	2.37E+08	1.4E+08	0.001204	1.699045346	1.7
6168	CD151_MOUSE	9170000	5386667	0.014099	1.702351485	1.7
6438	OGA_MOUSE	2820000	1663333	0.019283	1.695390782	1.7
6182	MBOA5_MOUSE	6233333	3676667	0.003665	1.695376247	1.7
7338	CREL1_MOUSE	3696667	2173333	0.003484	1.700920245	1.7
171	G6PI_MOUSE	3.31E+08	1.96E+08	0.000393	1.692504259	1.69
6459	SAP3_MOUSE	13866667	8196667	0.029181	1.691744612	1.69
303	P4HA2_MOUSE	1.63E+08	97133333	0.003752	1.681194235	1.68
941	E9QAT4_MOUSE	12400000	7390000	0.023853	1.677943166	1.68
3864	Q3TBV5_MOUSE	1.8E+08	1.08E+08	0.000843	1.675550046	1.68
4294	Q8C845_MOUSE	10916667	6486667	0.00729	1.682939363	1.68
5799	COTL1_MOUSE	17666667	10526667	6.27E-05	1.678277391	1.68
2865	SAFB2_MOUSE	1620000	964666.7	0.02324	1.679336558	1.68
7477	STS_MOUSE	5100000	3030000	0.036058	1.683168317	1.68
1979	MPPA_MOUSE	11090000	6633333	0.038045	1.671859296	1.67
2061	GGYF2_MOUSE	8313333	4986667	0.008722	1.667112299	1.67
11089	TYB4_MOUSE	56933333	34000000	0.001367	1.674509804	1.67
4497	E9QPX1_MOUSE	4480000	2680000	0.035303	1.671641791	1.67
8190	M4K5_MOUSE	733333.3	438333.3	0.016267	1.673003802	1.67
6	ACTB_MOUSE	5523333	3326667	0.020192	1.660320641	1.66

420	6PGD_MOUSE	1.66E+08	99866667	0.011705	1.660213618	1.66
982	NAMPT_MOUSE	34400000	20766667	0.018223	1.656500803	1.66
1527	D3Z4N0_MOUSE	14966667	9013333	0.001297	1.660502959	1.66
5482	PKHO2_MOUSE	10273333	6173333	0.014151	1.664146868	1.66
2539	SIL1_MOUSE	15033333	9100000	0.041051	1.652014652	1.65
5783	NPTN_MOUSE	22633333	13733333	0.007357	1.648058252	1.65
3738	KAD4_MOUSE	40000000	24366667	0.012532	1.641586867	1.64
2961	Q91Z50_MOUSE	18233333	11133333	0.004092	1.637724551	1.64
347	PFKAL_MOUSE	1.23E+08	74966667	0.002632	1.634504224	1.63
4835	SODM_MOUSE	47000000	28833333	0.000704	1.630057803	1.63
7059	PEA15_MOUSE	22233333	13600000	0.013977	1.634803922	1.63
8489	TRM5_MOUSE	13933333	8550000	0.001457	1.62962963	1.63
696	ARHG1_MOUSE	15766667	9706667	0.013735	1.624313187	1.62
4414	CANB1_MOUSE	13100000	8096667	0.026062	1.617949774	1.62
201	G6PD1_MOUSE	66500000	41333333	0.011829	1.608870968	1.61
666	SYK_MOUSE	58833333	36466667	0.001753	1.613345521	1.61
1106	TOIP1_MOUSE	33200000	20566667	0.049873	1.614262561	1.61
2288	EPHA2_MOUSE	9370000	5806667	0.031449	1.613662457	1.61
7611	NUBP1_MOUSE	3410000	2123333	0.029043	1.605965463	1.61
1631	RBGPR_MOUSE	11400000	7140000	0.046203	1.596638655	1.6
3350	A0A0R4J1D0_MOU	17966667	11236667	0.008459	1.598932068	1.6
5365	Q3U5D9_MOUSE	5780000	3616667	0.001566	1.598156682	1.6
5116	CDC73_MOUSE	2350000	1466667	0.006879	1.602272727	1.6
3041	PNPT1_MOUSE	4376667	2753333	0.022507	1.589588378	1.59
4302	PGES2_MOUSE	6950000	4360000	0.016685	1.594036697	1.59
2725	OGFR_MOUSE	12833333	8090000	0.021728	1.58632056	1.59
4613	RABP1_MOUSE	12300000	7713333	0.004437	1.594641314	1.59
7944	GALM_MOUSE	5030000	3156667	0.001319	1.59345301	1.59
2514	GSTP1_MOUSE	2.84E+08	1.8E+08	0.002705	1.576481481	1.58
144	PFKAP_MOUSE	94433333	59833333	0.000832	1.578272981	1.58
2504	DEST_MOUSE	3.75E+08	2.37E+08	0.012463	1.581153305	1.58
638	DDX21_MOUSE	30066667	19033333	0.018158	1.579684764	1.58
1382	PPGB_MOUSE	82600000	52400000	0.002142	1.576335878	1.58
1604	SMC2_MOUSE	9000000	5703333	0.025841	1.578024547	1.58
2701	PPME1_MOUSE	18233333	11523333	0.035403	1.582296789	1.58
1832	MRP1_MOUSE	6836667	4333333	0.006637	1.577692308	1.58
1237	IMA1_MOUSE	26533333	16866667	0.006411	1.57312253	1.57
2659	BLVRB_MOUSE	56100000	35766667	0.02038	1.568499534	1.57
7942	S10AG_MOUSE	8610000	5500000	0.009401	1.565454545	1.57
7279	CSF1_MOUSE	2193333	1400000	0.026522	1.566666667	1.57
4710	MIF_MOUSE	1.48E+09	9.49E+08	0.049891	1.563061798	1.56
1652	LDHB_MOUSE	32500000	20800000	0.014119	1.5625	1.56
3201	IF6_MOUSE	49366667	31733333	0.011524	1.555672269	1.56
7949	PSD10_MOUSE	3530000	2260000	0.002932	1.561946903	1.56
775	CATD_MOUSE	2.74E+08	1.77E+08	0.006593	1.547834275	1.55
2171	PTK7_MOUSE	16166667	10460000	0.002338	1.545570427	1.55
3859	RALB_MOUSE	9036667	5813333	0.019285	1.554472477	1.55
8484	AT1B1_MOUSE	2940000	1893333	0.011294	1.552816901	1.55
363	AT1A1_MOUSE	82900000	53900000	0.02855	1.538033395	1.54
1226	F8VQJ3_MOUSE	6190000	4023333	0.021484	1.538525269	1.54

6259	CNPY3_MOUSE	11833333	7666667	0.03477	1.543478261	1.54
8149	D3Z6H3_MOUSE	5653333	3673333	0.041032	1.539019964	1.54
8615	CASP6_MOUSE	1666667	1082000	0.018572	1.540357363	1.54
9083	IPP2_MOUSE	8716667	5646667	0.032737	1.543683589	1.54
1356	A2AEX8_MOUSE	70666667	46300000	0.025357	1.526277898	1.53
1587	DDAH1_MOUSE	56466667	37233333	0.001489	1.51656222	1.52
1111	MOV10_MOUSE	41500000	27300000	0.014335	1.52014652	1.52
1357	SQOR_MOUSE	25333333	16666667	5.99E-05	1.52	1.52
3951	UBP24_MOUSE	4426667	2916667	0.00866	1.517714286	1.52
4268	A2AH75_MOUSE	659333.3	434333.3	0.044118	1.518035303	1.52
4289	SCRB2_MOUSE	11466667	7553333	0.017054	1.518093557	1.52
839	TPIS_MOUSE	9.53E+08	6.33E+08	0.004095	1.505637513	1.51
1936	GLCM_MOUSE	32333333	21400000	0.000751	1.510903427	1.51
6222	SDF2L_MOUSE	14233333	9440000	0.005901	1.507768362	1.51
8601	ACYP2_MOUSE	6870000	4550000	0.016837	1.50989011	1.51
945	MAOM_MOUSE	17500000	11666667	0.003481	1.5	1.5
2334	TM9S2_MOUSE	35066667	52733333	0.003901	0.664981037	-1.5
3579	ARPC3_MOUSE	40033333	60200000	0.031667	0.665005537	-1.5
5017	AN32E_MOUSE	24766667	37200000	0.018145	0.665770609	-1.5
2044	DHX29_MOUSE	3180000	4813333	0.046216	0.66066482	-1.51
2830	ERLN1_MOUSE	10666667	16100000	0.043948	0.66252588	-1.51
191	GRP75_MOUSE	2.54E+08	3.87E+08	0.004053	0.656551724	-1.52
1074	VDAC1_MOUSE	1.92E+08	2.92E+08	0.039048	0.656571429	-1.52
1995	CDC42_MOUSE	1.4E+08	2.14E+08	0.01283	0.656074766	-1.52
771	PPID_MOUSE	32033333	48666667	0.024086	0.658219178	-1.52
3137	MBNL1_MOUSE	10446667	15833333	0.017663	0.659789474	-1.52
3555	FBXW8_MOUSE	3206667	4870000	0.013125	0.658453114	-1.52
3469	A0A0B4J1L4_MOUS	3113333	4743333	0.032668	0.656359803	-1.52
6280	RBBP9_MOUSE	5863333	8883333	0.014948	0.660037523	-1.52
428	ATLA3_MOUSE	37433333	57333333	0.008216	0.652906977	-1.53
942	ETFA_MOUSE	91866667	1.4E+08	0.049824	0.654631829	-1.53
3301	IMPA3_MOUSE	14300000	21933333	0.036054	0.651975684	-1.53
3005	KAD3_MOUSE	11433333	17466667	0.013637	0.654580153	-1.53
4055	F91A1_MOUSE	2270000	3480000	0.039339	0.652298851	-1.53
80	NEDD4_MOUSE	96933333	1.49E+08	0.000636	0.649107143	-1.54
828	PICAL_MOUSE	13933333	21466667	0.006123	0.649068323	-1.54
4961	RSMB_MOUSE	28566667	44100000	0.000826	0.647770219	-1.54
345	TRAP1_MOUSE	24066667	37333333	0.006485	0.644642857	-1.55
5016	RAB9A_MOUSE	4903333	7586667	0.004892	0.646309315	-1.55
7103	FKB14_MOUSE	1973333	3063333	0.003365	0.644178455	-1.55
7675	DEOC_MOUSE	1606667	2496667	0.03359	0.6435247	-1.55
1633	Q99N15_MOUSE	57233333	89100000	0.036019	0.64234942	-1.56
1866	M2OM_MOUSE	15666667	24500000	0.034955	0.639455782	-1.56
5120	E9QK83_MOUSE	775333.3	1206667	0.038199	0.642541436	-1.56
7489	Q3TSS7_MOUSE	1673333	2603333	0.012486	0.642765685	-1.56
2325	DDX50_MOUSE	2800000	4383333	0.023789	0.63878327	-1.57
660	IF4A3_MOUSE	31766667	50300000	0.042995	0.631544069	-1.58
2868	D3YW19_MOUSE	2820000	4456667	0.035211	0.63275991	-1.58
6034	NT5D3_MOUSE	1386667	2196667	0.012768	0.631259484	-1.58
5676	E9QQ10_MOUSE	550333.3	868000	0.049621	0.634024578	-1.58

3315	FNTA_MOUSE	4270000	6813333	0.000238	0.626712329	-1.6
4690	SMAD3_MOUSE	1426667	2280000	0.02487	0.625730994	-1.6
7542	J3QK13_MOUSE	1093333	1753333	0.030774	0.623574144	-1.6
8568	A2AJG0_MOUSE	702666.7	1123333	0.024986	0.625519288	-1.6
5905	STAR5_MOUSE	3386667	5446667	0.010028	0.621787026	-1.61
2982	D3YU59_MOUSE	3476667	5590000	0.041312	0.621943948	-1.61
6094	TRUA_MOUSE	1256667	2023333	0.007083	0.621087315	-1.61
8089	A2A8E2_MOUSE	5466667	8783333	0.020165	0.622390892	-1.61
1160	PURA2_MOUSE	20833333	33800000	0.008392	0.616370809	-1.62
1943	PCCA_MOUSE	5383333	8710000	0.010124	0.618063529	-1.62
3844	MARC2_MOUSE	8933333	14500000	0.041924	0.616091954	-1.62
729	GLSK_MOUSE	26733333	43533333	0.003076	0.614088821	-1.63
4563	PZP_MOUSE	12633333	20566667	0.034343	0.614262561	-1.63
2824	IPO11_MOUSE	5026667	8193333	0.028938	0.613506916	-1.63
4518	MCCB_MOUSE	2833333	4626667	0.009019	0.612391931	-1.63
6932	COX15_MOUSE	2310000	3760000	0.008181	0.614361702	-1.63
3475	BAG5_MOUSE	5980000	9816667	0.018731	0.609168081	-1.64
6521	MPC2_MOUSE	7790000	12800000	0.002031	0.60859375	-1.64
2003	ITA1_MOUSE	7216667	11900000	0.008414	0.606442577	-1.65
4637	ASPC1_MOUSE	3906667	6446667	0.015276	0.605997932	-1.65
328	Q80YQ1_MOUSE	44700000	74400000	0.019619	0.600806452	-1.66
2724	Q9ESU7_MOUSE	16700000	27800000	0.000753	0.600719424	-1.66
5368	A0A0R4IZY9_MOUS	1516667	2516667	0.024241	0.602649007	-1.66
1096	PSB5_MOUSE	32833333	54833333	0.014494	0.598784195	-1.67
1311	STEA3_MOUSE	18833333	31366667	0.007052	0.60042508	-1.67
3017	DOPD_MOUSE	20066667	33433333	1.31E-05	0.600199402	-1.67
1752	AP3M1_MOUSE	9236667	15433333	0.026832	0.598488121	-1.67
996	HMCS1_MOUSE	13366667	22400000	0.040137	0.59672619	-1.68
2823	MMP14_MOUSE	7890000	13266667	0.000631	0.594723618	-1.68
6039	E9Q179_MOUSE	2136667	3583333	0.049208	0.59627907	-1.68
12863	G3X8S8_MOUSE	2170000	3640000	0.044704	0.596153846	-1.68
767	ACACA_MOUSE	6883333	11633333	0.000692	0.591690544	-1.69
2165	ECHM_MOUSE	30233333	51133333	0.00532	0.591264668	-1.69
2269	THUM1_MOUSE	5686667	9586667	0.001852	0.593184979	-1.69
6228	FKBP2_MOUSE	18633333	31566667	0.016029	0.590285111	-1.69
2423	DNMT1_MOUSE	3826667	6510000	0.043065	0.58781362	-1.7
2258	CTGF_MOUSE	8813333	14966667	0.007684	0.588864143	-1.7
6284	MTMR2_MOUSE	1403333	2380000	0.024417	0.589635854	-1.7
282	E9PWE8_MOUSE	18800000	32166667	0.017006	0.584455959	-1.71
8023	NOV_MOUSE	622666.7	1063333	0.009253	0.585579937	-1.71
8161	ARXS1_MOUSE	3596667	6153333	0.007027	0.584507042	-1.71
8318	CATK_MOUSE	1310000	2243333	0.043567	0.583952452	-1.71
7129	Q3TLQ0_MOUSE	1333333	2290000	0.009594	0.58224163	-1.72
456	E9QAH1_MOUSE	6980000	12066667	0.03641	0.578453039	-1.73
2799	A0A1L1SS90_MOUS	3626667	6256667	0.024007	0.579648375	-1.73
4552	STRN_MOUSE	2990000	5160000	0.019347	0.579457364	-1.73
4081	SRPK1_MOUSE	1836667	3173333	0.044933	0.578781513	-1.73
8160	NTPCR_MOUSE	1363333	2353333	0.017334	0.579320113	-1.73
4867	RAD21_MOUSE	3116667	5416667	0.0202	0.575384615	-1.74
5574	DPP9_MOUSE	1763333	3066667	0.005825	0.575	-1.74

7028	SFR1_MOUSE	1200000	2086667	0.005991	0.575079872	-1.74
3219	CUL7_MOUSE	3093333	5423333	0.027211	0.570374923	-1.75
4154	MEP50_MOUSE	5603333	9833333	0.007093	0.569830508	-1.75
8960	EPT1_MOUSE	3366667	5886667	0.028486	0.57191393	-1.75
567	IDHP_MOUSE	45200000	79366667	0.007101	0.56950861	-1.76
4899	IST1_MOUSE	5443333	9563333	0.005867	0.56918787	-1.76
7289	MD1L1_MOUSE	336000	592333.3	0.012498	0.567248171	-1.76
5305	RRFM_MOUSE	2803333	4963333	0.004843	0.564808596	-1.77
7836	CE051_MOUSE	2056667	3650000	0.01732	0.56347032	-1.77
13097	RRP15_MOUSE	1064000	1886667	0.024844	0.563957597	-1.77
8669	CRNL1_MOUSE	365000	647333.3	0.033356	0.563851699	-1.77
3107	A0A0A6YY47_MOU	5690000	10133333	0.01346	0.561513158	-1.78
7905		2170000	3873333	0.000324	0.560240964	-1.78
7578	RT07_MOUSE	2320000	4133333	0.013456	0.561290323	-1.78
1718	YBOX3_MOUSE	18933333	33800000	0.013014	0.560157791	-1.79
4676	GNA1_MOUSE	14933333	26700000	0.006108	0.559300874	-1.79
8215	USE1_MOUSE	1463333	2616667	0.016333	0.559235669	-1.79
504	LCAP_MOUSE	11800000	21200000	0.010619	0.556603774	-1.8
3938	Q8C6B0_MOUSE	9133333	16466667	5.44E-05	0.55465587	-1.8
740	HUWE1_MOUSE	6420000	11603333	0.023121	0.553289285	-1.81
2106	NASP_MOUSE	10233333	18566667	0.004049	0.551166966	-1.81
8143	IFRD1_MOUSE	656000	1190000	0.002492	0.551260504	-1.81
6295	A0A0U1RP62_MOU	3510000	6440000	0.038866	0.545031056	-1.83
6157	ILKAP_MOUSE	1646667	3036667	0.012833	0.542261251	-1.84
2787	ACSL3_MOUSE	4126667	7623333	0.005455	0.541320507	-1.85
5941	EBP2_MOUSE	1330000	2466667	0.016306	0.539189189	-1.85
500	E9PUM4_MOUSE	6443333	11966667	0.043145	0.538440111	-1.86
6223	B9EKS2_MOUSE	1116667	2080000	0.022255	0.536858974	-1.86
7481	RM04_MOUSE	1220000	2280000	0.030261	0.535087719	-1.87
2217	CAN6_MOUSE	6233333	11700000	0.006297	0.532763533	-1.88
2860	WDR11_MOUSE	2506667	4723333	0.002913	0.530698659	-1.88
5668	MCCA_MOUSE	2643333	4960000	0.029019	0.532930108	-1.88
179	SYAC_MOUSE	75500000	1.43E+08	0.000508	0.527972028	-1.89
6522	NDUA7_MOUSE	4700000	9033333	0.045327	0.520295203	-1.92
5249	RABE2_MOUSE	1940000	3750000	0.000272	0.517333333	-1.93
12465	ANKY2_MOUSE	1242667	2410000	0.023406	0.515629322	-1.94
2633	CHIP_MOUSE	6720000	13100000	0.010113	0.512977099	-1.95
267	MYH10_MOUSE	42533333	83300000	0.009236	0.510604242	-1.96
6252	FCHO2_MOUSE	857000	1683333	0.04262	0.509108911	-1.96
75	PCKGM_MOUSE	73633333	1.46E+08	0.02668	0.5043379	-1.98
5600	NDUS8_MOUSE	8066667	15933333	0.025868	0.506276151	-1.98
591	LMCD1_MOUSE	28866667	57466667	1.1E-08	0.502320186	-1.99
320	CO5A2_MOUSE	18066667	36066667	0.000739	0.500924214	-2
3804	CSAD_MOUSE	2470000	4930000	0.003326	0.501014199	-2
7533	CGRE1_MOUSE	4940000	9893333	0.017552	0.499326146	-2
2412	SERB_MOUSE	19933333	40100000	0.022998	0.497090607	-2.01
6352	UBE2H_MOUSE	5636667	11340000	0.020662	0.497060553	-2.01
851	4F2_MOUSE	38900000	78533333	0.010065	0.49533107	-2.02
5474	SYTM_MOUSE	1049000	2116667	0.008722	0.495590551	-2.02
3563	PPR1B_MOUSE	11033333	22366667	0.02377	0.493293592	-2.03

3358	E9PX53_MOUSE	1866667	3790000	0.028929	0.492524186	-2.03
2782	MYL9_MOUSE	5716667	11646667	0.030652	0.490841442	-2.04
6842	A2AQV2_MOUSE	1336667	2723333	0.000205	0.490820073	-2.04
6376	SYRM_MOUSE	815333.3	1660000	0.007303	0.491164659	-2.04
6029	RLA1_MOUSE	8650000	17700000	0.000839	0.488700565	-2.05
5106	E9PWY6_MOUSE	2176667	4470000	0.012126	0.486950037	-2.05
1795	GALE_MOUSE	6636667	13700000	0.007633	0.484428224	-2.06
2476	MYO1B_MOUSE	5290000	10893333	0.030274	0.485618115	-2.06
2699	PEPD_MOUSE	4160000	8580000	0.048476	0.484848485	-2.06
5203	A2BG76_MOUSE	1850000	3806667	0.003954	0.485989492	-2.06
7465	G3UZK1_MOUSE	2500000	5143333	0.006453	0.486066105	-2.06
1709	PCCB_MOUSE	5873333	12133333	0.011893	0.484065934	-2.07
8313	MSPD2_MOUSE	1896667	3916667	0.044804	0.484255319	-2.07
1674	NB5R1_MOUSE	18866667	39333333	0.001982	0.479661017	-2.08
385	CO5A1_MOUSE	37866667	79000000	0.000697	0.479324895	-2.09
335	NIBAN_MOUSE	36333333	75900000	0.008662	0.478700044	-2.09
2133	TGFI1_MOUSE	9956667	20766667	0.027042	0.479454254	-2.09
5999	NDUA4_MOUSE	26500000	55433333	0.02359	0.478051714	-2.09
5839	RM37_MOUSE	3240000	6786667	0.001613	0.47740668	-2.09
5814	RIOK1_MOUSE	634000	1323333	0.026355	0.479093199	-2.09
4045	Q9CPX4_MOUSE	8500000	17966667	0.015943	0.47309833	-2.11
3	TBA1A_MOUSE	34600000	73866667	0.03566	0.468411552	-2.13
5786	RO60_MOUSE	2883333	6146667	0.049482	0.469088937	-2.13
7165	GL8D1_MOUSE	1723333	3663333	0.028502	0.470427662	-2.13
12557	H3BJV9_MOUSE	1510000	3213333	0.035541	0.469917012	-2.13
168	AL1L2_MOUSE	36400000	78100000	0.000362	0.466069142	-2.15
3802	COBA2_MOUSE	1536667	3306667	0.026278	0.464717742	-2.15
5552	Q80ZM5_MOUSE	1260000	2713333	0.023595	0.464373464	-2.15
3328	MARCS_MOUSE	1.04E+08	2.24E+08	0.005467	0.462891207	-2.16
3993	DIP2B_MOUSE	1103000	2400000	0.038835	0.459583333	-2.18
6112	Q8C4U8_MOUSE	965333.3	2100000	0.030352	0.45968254	-2.18
3998	F7DEU6_MOUSE	2013333	4433333	0.02297	0.454135338	-2.2
1982	STK26_MOUSE	2450000	5433333	0.016062	0.450920245	-2.22
5480	EMIL1_MOUSE	952666.7	2120000	0.010317	0.449371069	-2.23
5898	IMPCT_MOUSE	4883333	10950000	0.04177	0.445966514	-2.24
1040	PHLB2_MOUSE	12590000	28266667	0.032196	0.445400943	-2.25
6484	Q99L96_MOUSE	3050000	6990000	0.035494	0.436337625	-2.29
6563	ITPR1_MOUSE	520000	1193333	0.009491	0.43575419	-2.29
1405	GNS_MOUSE	25533333	58833333	0.003719	0.433994334	-2.3
3632	BCAT1_MOUSE	5500000	12700000	0.017716	0.433070866	-2.31
1176	P5CR1_MOUSE	17433333	40566667	0.043011	0.429745275	-2.33
10099	Q60709_MOUSE	313000	729000	0.0087	0.429355281	-2.33
891	ACOT2_MOUSE	12366667	28933333	0.002983	0.427419355	-2.34
5967	RECK_MOUSE	2566667	6013333	0.035161	0.426829268	-2.34
8059	FBX4_MOUSE	648000	1530000	0.013312	0.423529412	-2.36
2590	A0A1L1STC6_MOUS	1020667	2466667	0.022068	0.413783784	-2.42
3436	PPBT_MOUSE	2266667	5486667	0.006844	0.413122722	-2.42
5470	MK67I_MOUSE	4630000	11256667	0.021708	0.411311815	-2.43
5312	H7BX23_MOUSE	1423333	3476667	0.006616	0.409395973	-2.44
8429	PXDN_MOUSE	371000	905000	0.000111	0.409944751	-2.44

3806	A0A0G2JE03_MOU!	3103333	7596667	1.38E-05	0.408512505	-2.45
5889	TM87A_MOUSE	4500000	11070000	0.033526	0.406504065	-2.46
4760	SNX17_MOUSE	981666.7	2423333	0.001737	0.405089409	-2.47
8677	NTAN1_MOUSE	159000	392333.3	0.042111	0.40526763	-2.47
7205	Q3URQ4_MOUSE	440000	1097667	0.027773	0.400850288	-2.49
1556	ALAT2_MOUSE	5790000	14466667	0.032052	0.400230415	-2.5
3122	ELP2_MOUSE	2106667	5273333	0.0092	0.399494311	-2.5
7301	WDR75_MOUSE	1056000	2640000	0.033896	0.4	-2.5
4397	GMPR2_MOUSE	3346667	8446667	0.002021	0.396211523	-2.52
4721	DJC11_MOUSE	2963333	7473333	0.041579	0.396520963	-2.52
6162	D39U1_MOUSE	792333.3	2010000	0.040161	0.394195688	-2.54
1762	A0A0R4J2B2_MOU!	13166667	34166667	0.019317	0.385365854	-2.59
5027	TSP2_MOUSE	320333.3	835000	0.027352	0.383632735	-2.61
1813	KDEL1_MOUSE	5840000	15733333	0.04398	0.371186441	-2.69
5467	G3UW90_MOUSE	1395333	3786667	0.014953	0.368485915	-2.71
1198	GRB10_MOUSE	8766667	23833333	0.003383	0.367832168	-2.72
4645	XPO4_MOUSE	728000	1996667	0.034898	0.364607679	-2.74
8492	GP180_MOUSE	322000	888333.3	0.032744	0.362476548	-2.76
1620	SUCB2_MOUSE	8810000	24500000	0.006868	0.359591837	-2.78
6970	S2551_MOUSE	679000	1916667	0.024149	0.35426087	-2.82
6035	IRF3_MOUSE	1033333	2946667	0.000499	0.350678733	-2.85
1466		4296667	12300000	0.00691	0.349322493	-2.86
1782	MAP2_MOUSE	2783333	8040000	0.011845	0.346185738	-2.89
5456	A0A1Y7VNM7_MO	1669333	4823333	0.020366	0.34609537	-2.89
256	COBA1_MOUSE	25300000	75133333	0.009373	0.336734694	-2.97
197	COCA1_MOUSE	75266667	2.27E+08	0.003404	0.332058824	-3.01
17	CO1A2_MOUSE	76133333	2.32E+08	0.003491	0.328633094	-3.04
5323	PAOX_MOUSE	1270000	3886667	0.008852	0.326758148	-3.06
2570	A2AJQ0_MOUSE	1406667	4336667	5.05E-05	0.324365872	-3.08
50	CO1A1_MOUSE	1.29E+08	3.99E+08	0.00328	0.324040067	-3.09
5767	G3X920_MOUSE	807333.3	2496667	0.01723	0.323364486	-3.09
10803	NOL9_MOUSE	633333.3	1966667	0.000527	0.322033898	-3.11
3100	ITB5_MOUSE	3260000	10456667	0.015362	0.311762831	-3.21
5182	A0A0R4J1B9_MOU!	2306667	7616667	0.009676	0.302844639	-3.3
10618	I2BP1_MOUSE	206666.7	682666.7	0.020647	0.302734375	-3.3
7801	PLRKT_MOUSE	1170000	3873333	0.000115	0.302065404	-3.31
2046	COG2_MOUSE	2270000	7796667	0.041763	0.291150064	-3.43
8428	LTV1_MOUSE	1016333	3483333	0.002413	0.291770335	-3.43
3829	Q8VCG1_MOUSE	6696667	24533333	0.002632	0.272961957	-3.66
1764	CP51A_MOUSE	5476667	20500000	0.019921	0.267154472	-3.74
7834	VP37A_MOUSE	284666.7	1090333	0.018388	0.261082238	-3.83
7358	PWP2_MOUSE	252666.7	973666.7	0.026358	0.259500171	-3.85
1882	Q543V3_MOUSE	1566667	6166667	0.020058	0.254054054	-3.94
1432	ACADS_MOUSE	8276667	32900000	0.003014	0.251570415	-3.98
8112	EYA4_MOUSE	495000	1990000	0.024057	0.248743719	-4.02
9137	Q9DC42_MOUSE	1087667	4376667	0.021025	0.248514851	-4.02
4535	P3H2_MOUSE	1042000	4260000	0.02835	0.244600939	-4.09
6425	B1B1A8_MOUSE	571666.7	2350000	0.00536	0.243262411	-4.11
7871	ITGBL_MOUSE	281333.3	1210667	0.015156	0.232378855	-4.3
43	CO2A1_MOUSE	95033333	4.17E+08	0.037305	0.22808	-4.38

3200	CTHR1_MOUSE	3126667	13933333	0.002274	0.224401914	-4.46
3687	SRGP2_MOUSE	584666.7	2670000	0.021396	0.21897628	-4.57
7545	E9Q7S4_MOUSE	1256667	5940000	0.000201	0.211560045	-4.73
9841	BCS1_MOUSE	231000	1124667	0.043312	0.205394191	-4.87
6165	POSTN_MOUSE	631333.3	3103333	0.004414	0.203437164	-4.92
6416	D3Z5M8_MOUSE	358333.3	1786667	0.046114	0.200559701	-4.99
5579	ADH7_MOUSE	599666.7	3033333	0.034012	0.197692308	-5.06
6323	Q91V35_MOUSE	525000	2683333	0.02876	0.195652174	-5.11
3648	MIME_MOUSE	1136667	5933333	0.004212	0.191573034	-5.22
3749	GSTA1_MOUSE	4960000	26200000	0.0386	0.189312977	-5.28
1618	G3X995_MOUSE	3466667	18300000	7.55E-06	0.189435337	-5.28
7957	LAT1_MOUSE	418000	2266667	0.003128	0.184411765	-5.42
4049	ITA2_MOUSE	556333.3	3150000	0.041182	0.176613757	-5.66
7191	RAD50_MOUSE	127333.3	840666.7	0.039041	0.15146709	-6.6
5721	KCC4_MOUSE	329666.7	2376667	0.049928	0.138709677	-7.21
4861	Q59IW9_MOUSE	450666.7	3590000	0.006639	0.12553389	-7.97
9624	A0A1L1STF4_MOUS	6066.667	403666.7	0.018453	0.015028902	-66.54
