# Supplementary material for: TGFβ attenuates cartilage extracellular matrix degradation via enhancing FBXO6-mediated MMP14 ubiquitination
Source: Ann Rheum Dis. 2020 May 14;79(8):1111–20. doi: 10.1136/annrheumdis-2019-216911 (PMC7392491; doi:10.1136/annrheumdis-2019-216911)
Supplement: Supplementary data [file annrheumdis-2019-216911supp013.pdf]

Origin	Gene	Strand	Primer Sequences
Mouse	ADAMTS4	forward	5’-CAGTCACCTCTAAGCCAAAGAAA-3’
		reverse	5’-CTTCCGGCGTAGGATGTGAG-3’
	ADAMTS5	forward	5’-CCTTTGCTTGCTTTCTTTCC-3’
		reverse	5’-CTCCGACCCACTTCCTTTCT-3
	MMP3	forward	5’-TTAAAGACAGGCACTTTTGGCG-3’
		reverse	5’-CCCTCGTATAGCCCAGAACT-3’
	MMP13	forward	5’-TGTTTGCAGAGCACTACTTGAA-3’
		reverse	5’-CAGTCACCTCTAAGCCAAAGAAA-3’
	Col2A1	forward	5’-GAGCAGCAAGAGCAAGGAAA-3’
		reverse	5’-CGGAGGAAAGTCATCTGGAC-3’
	Aggrecan	forward	5’-GGGACAGGGTGAGAAGTAAGG-3’
		reverse	5’-CTGGAGGCGAAGTAACCAAC-3’
	Sox9	forward	5’-GAGGAAGTCGGTGAAGAACG-3’
		reverse	5’-GAGGAGGAATGTGGGGAGT-3’
	18S	forward	5’-AGGGGAGAGCGGGTAAGAGA-3’
		reverse	5’-GGACAGGACTAGGCGGAACA-3’
		reverse	5’-AGAGCTCCGAGCTCACCATC-3’
	FBXO6	forward	5’- TTGTCACCTCTTTTGAGATGTGC -3’
		reverse	5’- CCAGTCCTTAACCACAATGTCAG -3’
Human	18S	forward	5’-CCTGCGGCTTAATTTGACTC-3’
		reverse	5’-AACTAAGAACGGCCATGCAC-3’
	FBXO2	forward	5’- GTAACCCGTGTGGGGAAGAG -3’
		reverse	5’- TCTTGACGCTCTCATCGTGG -3’
	FBXO17	forward	5’- ATCTGTGTGGCTGACTGGTG -3’
		reverse	5’- ATACGTAGCGGATGCCCTTG -3’
	FBXO27	forward	5’- CGTCCAACTTCTAGACGCCA -3’
		reverse	5’- TTGGAGAACACGTGGGTGAC -3’
	FBXO44	forward	5’- TGAATGGAGGCGATGAGTGG -3’
		reverse	5’- GAGGCAGGTGCTGGTCATTG -3’
	FBXO6	forward	5’-GAGACAGCTTCAGGACACGCA-3’
		reverse	5’-CGGGCAGCTCGTTAATGCTG-3’
